# Casein kinase 1α mediates estradiol secretion via CYP19A1 expression in mouse ovarian granulosa cells

**DOI:** 10.1186/s12915-024-01957-3

**Published:** 2024-08-26

**Authors:** Xuan Luo, Di Zhang, Jiaming Zheng, Hui Liu, Longjie Sun, Hongzhou Guo, Lei Wang, Sheng Cui

**Affiliations:** 1 College of Veterinary Medicine, Yangzhou University, Yangzhou, Jiangsu 225009 People’s Republic of China; 2 State Key Laboratory of Agrobiotechnology, College of Biological Sciences, China Agricultural University, 100193 Beijing, People’s Republic of China; 3 Jiangsu Co-Innovation Center for Prevention and Control of Important Animal Infectious Diseases and Zoonoses, Yangzhou University, Yangzhou, 225009 People’s Republic of China; 4 Academy of Animal Science and Veterinary Medicine, Qinghai University, Xining, 810016 China

**Keywords:** Casein kinase 1α, Ovarian granulosa cells, Estrogen synthetase, Estradiol, Cytochrome P450 subfamily 19 member 1

## Abstract

**Background:**

Casein kinase 1α (CK1α), expressed in both ovarian germ and somatic cells, is involved in the initial meiosis and primordial follicle formation of mouse oocytes. Using in vitro and in vivo experiments in this study, we explored the function and mechanism of CK1α in estrogen synthesis in mice ovarian granulosa cells.

**Methods:**

A CK1α knockout (cKO) mouse model, targeted specifically to ovarian granulosa cells (GCs), was employed to establish the influence of CK1α on in vivo estrogen synthesis*.* The influence of CK1α deficiency on GCs was determined in vivo and in vitro by immunofluorescence analysis and Western blot assay. Transcriptome profiling, differentially expressed genes and gene functional enrichment analyses, and computation protein–protein docking, were further employed to assess the CK1α pathway. Furthermore, wild-type female mice were treated with the CK1α antagonist D4476 to elucidate the CK1α's role in estrogen regulation.

**Results:**

Ovarian GCs CK1α deficiency impaired fertility and superovulation of female mice; also, the average litter size and the estradiol (E_2_) level in the serum of cKO female mice were decreased by 57.3% and 87.4% vs. control mice, respectively.

This deficiency disrupted the estrous cycle and enhanced the apoptosis in the GCs. We observed that CK1α mediated the secretion of estradiol in mouse ovarian GCs via the cytochrome P450 subfamily 19 member 1 (CYP19A1).

**Conclusions:**

These findings improve the existing understanding of the regulation mechanism of female reproduction and estrogen synthesis.

**Trial registration:**

Not applicable.

**Supplementary Information:**

The online version contains supplementary material available at 10.1186/s12915-024-01957-3.

## Background

Estrogens, a group of sex hormones, are essential for normal sexual and reproductive development in women. There are principally three types of estrogens: estrone (E_1_), estradiol (E_2_), and estriol (E_3_). Estradiol (E_2_) is the most pervasive and potent, whereas estriol (E_3_) has the lowest bioactivity, approximately 1% of that of E_2_ [1]. Estrogens are secreted by ovarian granulosa cells (GCs) [2] and are indispensable for the development of the reproductive system [3], the maintenance of secondary sexual characteristics [4], follicular development, and embryo implantation [5, 6]. Furthermore, estrogens are crucially important for the modulation of the estrous cycle and parturition [7, 8].

Estrogen is produced using a tightly regulated process, during which luteinizing hormone (LH) acts on thecal cells to produce androgen, which enters GCs, where it is converted to estrogens in follicle-stimulating hormone (FSH) exposed GC [9]. The main action of estrogens is regulated by the estrogen receptors (ERα or ERβ). In the classical estrogen regulatory pathway, estrogen enters the cells and binds to the compatible receptor, which leads to dimerization followed by nuclear localization. Once in the nucleus, it binds directly to the DNA response elements, such as the estrogen response element (ERE), and regulates the transcription of target genes through the two activation domains AF-1 and AF-2 [10]. Estrogen is also regulated by an alternate mechanism, *i.e.*, the "noncanonical" estrogen regulatory pathway (also known as the "membrane signaling" pathway), during which the G-protein-coupled receptor-ER1-coupled pathway (GPER, also known as GPR30) [11] activates epidermal growth factor receptor (EGFR) and mitogen-activated protein kinase (MAPK) kinases, and induces rapid phosphorylation of MEK1/2 and ERK1/2 by inducing the release of heparin-bound EGF [12, 13]. In addition, another estradiol signaling mechanism, also called nongenomic with rapid effects, The ligand activates a receptor (ER, ER isoform or a distinct receptor), or a signal activates a classical ER located in the cytoplasm, and the signaling cascades are initiated that affect ion channels or increase nitric oxide levels in the cytoplasm, and this ultimately leads to a rapid physiological response without gene regulation involvement [14].

Estrogen biosynthesis involves a series of reactions catalyzed by steroidogenic enzymes. Notably, cytochrome P450 subfamily 19 member 1 (CYP19A1), the enzyme responsible for the conversion of androgens to estrogens, plays a pivotal role in the final step of E_2_ biosynthesis in GCs [15].

Casein kinases (CK), including CK1 and CK2, are serine/threonine protein kinases [16, 17]. In mammals, seven members of the CK1 family have been found, including α, β, γ1, γ2, γ3, δ, and ε. Each of these members is encoded by a unique gene and has a different molecular weight [18]. For example, CK1α, encoded by the *Csnk1a1* gene, has an isoelectric point greater than 9, which enables binding to acidic amino acid substrates. CK1α is ubiquitously present in a number of organelles [19], such as the centrosomes, endoplasmic reticulum, Golgi apparatus [20], spindle apparatus [21], and neurons [22]. Studies have shown that CK1α has an important regulatory role in intracellular signal transduction and signaling pathways [16], such as Wnt/β-catenin [23], NF-κB [24], PTEN/AKT [25], and p53 [26]; cytoskeleton maintenance [17], cell cycle progression [27], biological circadian rhythm regulation, DNA replication, and stress damage response [28]. Moreover, recent studies have shown that CK1α is expressed in both germ cells and somatic cells, where it regulates murine spermatogenesis [29] and participates in primordial follicle formation [30]. However, whether CK1α is involved in the regulation of estrogen synthesis in ovarian GCs has not been elucidated.

This research investigated the role of CK1α in estrogen synthesis by employing the GCs-specific CK1α knockout (cKO) mouse model in in vitro and in vivo experiments. The results indicated that CK1α positively regulates estradiol secretion by modulating the expression of CYP19A1 in mouse ovarian granulosa cells.

## Results

### Establishment of an ovarian GCs-Specific CK1α Knockout (cKO) mouse model

**Fig. 1:**
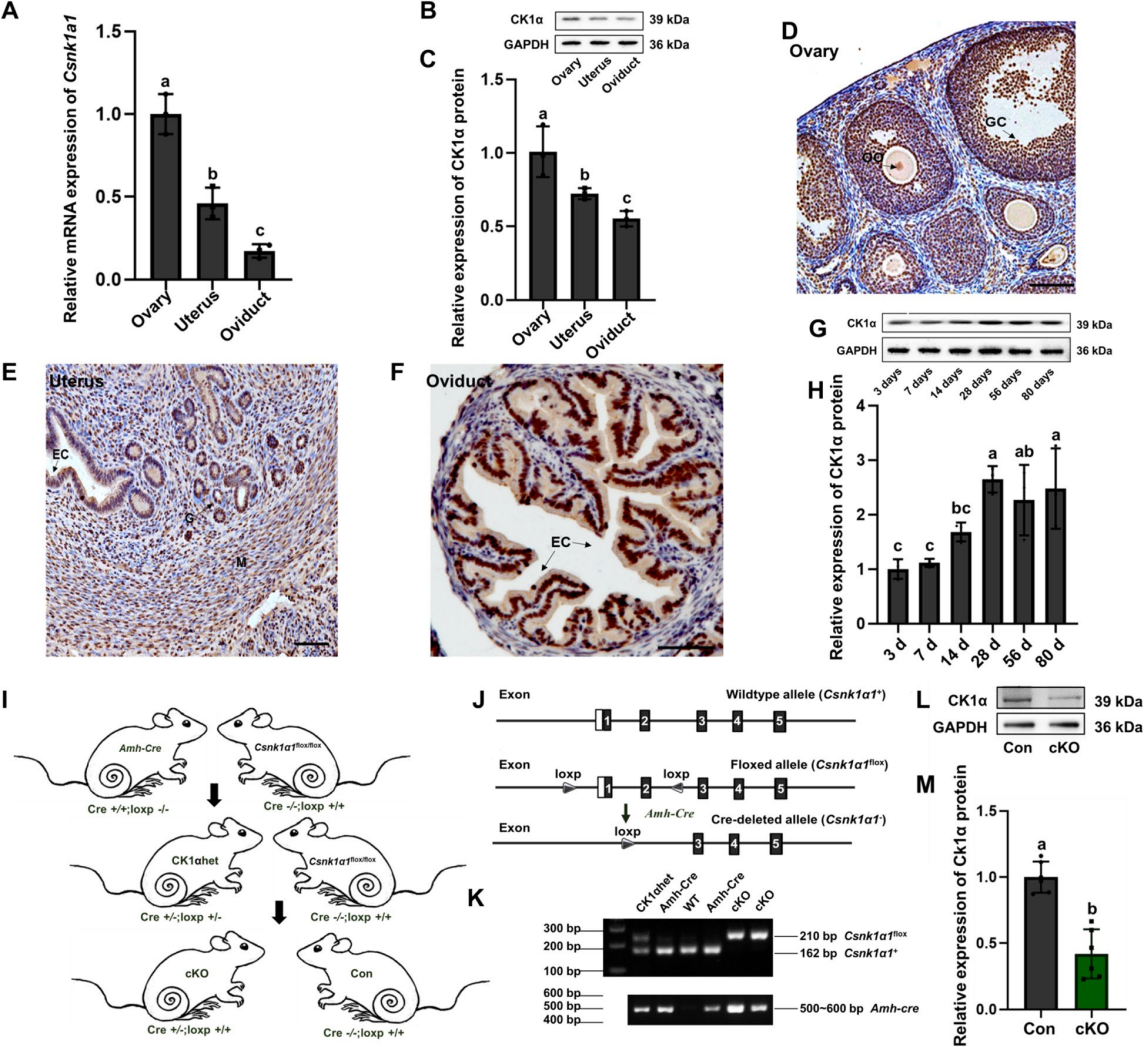
**A-I** CK1α expression in the mouse ovarian GCs. **A** CK1α mRNA levels in different mice tissues were analyzed by RT-qPCR and normalized to Gapdh. **B**, **C** CK1α protein levels in different mice tissues detected by WB. GAPDH was used as the loading control, and relative protein levels were analyzed by gray scanning. **D-F** The localization of CK1α in the ovary, uterus and oviduct of adult mouse detected by IHC. Paraffin slices of tissues were incubated with CK1α antibodies, and antibodies were replaced with anti-rabbit IgG for negative control. Selectively staining brown demonstrated CK1α-positive signals. Scale bar = 100 μm. Each tissue has three biological replicates. **G**, **H** CK1α protein abundances from ovary tissues at 3, 7, 14, 28, 56, and 80 days analyzed by WB; GAPDH was used as the internal reference, and relative protein levels were analyzed by gray scanning. Results are presented as means ± standard deviation (SD) of three independent biological experiments. The same letters indicate the difference is not significant, and the different letters (a, b and c) between the two bars show a significant difference (a versus b, b versus c: *P* < 0.05). **I-M** Establishment of ovarian GCs*-*specific cKO mouse model. **I** Schematic of the simplified strategy for creating the cKO (Cre ± ; loxp + / +). **J** Strategies for the construction of cKO mice, Csnk1α1^flox/flox^ mice with exon 1–2 of Csnk1α1 flanked with two loxP sites; the numbered black boxes represent exons while white boxes represent promoter. **K** Genotyping using PCR. The target band size of *Csnk1α1*.^flox/flox^ mice was 210 bp, and the wild band was 162 bp. The *Amh*-Cre target bands ranged from 500–600 bp. **L** Representative image of WB detecting the knockdown efficiency of CK1α protein inside ovarian GCs in vivo. **M** Relative protein levels were analyzed by gray scanning and normalized to GAPDH. Results are presented as means ± SD of three independent biological experiments. Different letters represent a significant difference (*P* < 0.05)

To determine the effects of CK1α on the female reproductive system, we assayed the CK1α expression in the ovary, uterus, and oviduct using RT-qPCR (Fig. 1A) and WB (Fig. 1B, C). CK1α was expressed in the female reproductive system, and the relative protein expression in the ovary was higher than in other tissues (all *P* < 0.05). The immunohistochemical (IHC) results further demonstrated that CK1α was located in ovarian GCs (Fig. 1D), uterine epithelial cells (Fig. 1E), and oviductal epithelial cells (Fig. 1F). Meanwhile, the CK1α antibodies were replaced with anti-rabbit IgG (negative control) (Additional file 1: Figure S1).

Next, we examined the expression of CK1α in 3-, 7-, 14-, 28-, 56-, and 80-day (d)-old mice ovaries by WB. CK1α was temporally expressed in the ovarian organization, and CK1α was much higher expressed in the ovaries of mature mice (Fig. 1G, H) (all *P* < 0.05). and when the mice reach sexual maturity (28 days of age), the expression level of CK1α protein in the ovaries tends to be stable. Results show that CK1α was expressed in ovarian GCs of the follicle (Additional file 2: Figure S2), and what role does CK1α play in ovarian GCs?

To explore the functions of CK1α in ovarian GCs, we generated cre-recombinase mouse model by specifically deleting the *Csnk1a1* gene in ovarian GCs. *Csnk1α1*^flox/flox^ (loxp + / +) mice [31] were crossed with *Amh*-Cre (Cre + / +) mice to produce *Amh-Cre* male breeders with a single recombined *Csnk1α1* allele (Cre ± ;loxp ±). Males with specific and robust Cre expression were then crossed with *Csnk1α1*^*flox/flox*^ females to generate conditional *Csnk1α1* knockout mice (Cre ± ; loxp + / + or cKO), and mice with loxp but no cre gene were the controls (Cre-/-; loxp + / + or Con) (Fig. 1I). The paternal line was later maintained by crossing with C57BL/6 females. Finally, *Csnk1α1*^*flox/flox*^ mice were maintained by breeding *Csnk1α1*^*flox/flox*^ male and female mice. Schematic of wild-type and *Csnk1α1*-mutant alleles with numbered black boxes represent exons while white boxes represent promoter. *Csnk1α1*^*flox/flox*^ mice with exon 1–2 of *Csnk1α1* flanked with two loxp sites were crossbred with *Amh*-Cre mice (Fig. 1J). Genotyping and gene identification were performed by PCR, and the band positions were analyzed by gel electrophoresis. The genotyping results contained *Csnk1α1*^*flox/flox*^ and *Amh*-Cre target bands (mice with Cre and loxp fragments, Cre ± ; loxp + / +), which were identified as cKO mice (Fig. 1K). To explore the specific period of CK1α knockdown in ovarian GCs, deletion efficiency of cKO mice ovaries at different developmental stages (three days, one weeks, four weeks, and eight weeks) were detected by WB and IF staining (Additional file 3: Figure S3), and results showed that CK1α protein levels decreased (Fig. 1L, M) (*P* < 0.05) in adult female cKO mice ovaries (eight weeks). To verify the knockdown specificity of CK1α in ovaries, the expression of CK1α in the uterus and oviduct of eight-week-old cKO mice were detected by immunohistochemistry, and the results showed that there were CK1α positive signals expressed in the uterus and fallopian tubes of cKO female mice (Additional file 4: Figure S4). These results demonstrated that CK1α was efficiently and specifically knockdown in adult cKO mice ovarian GCs.

### Ovarian GCs CK1α reduction disturbs mouse estrus cycle and reduces the ovulation rate and litter size

**Fig. 2:**
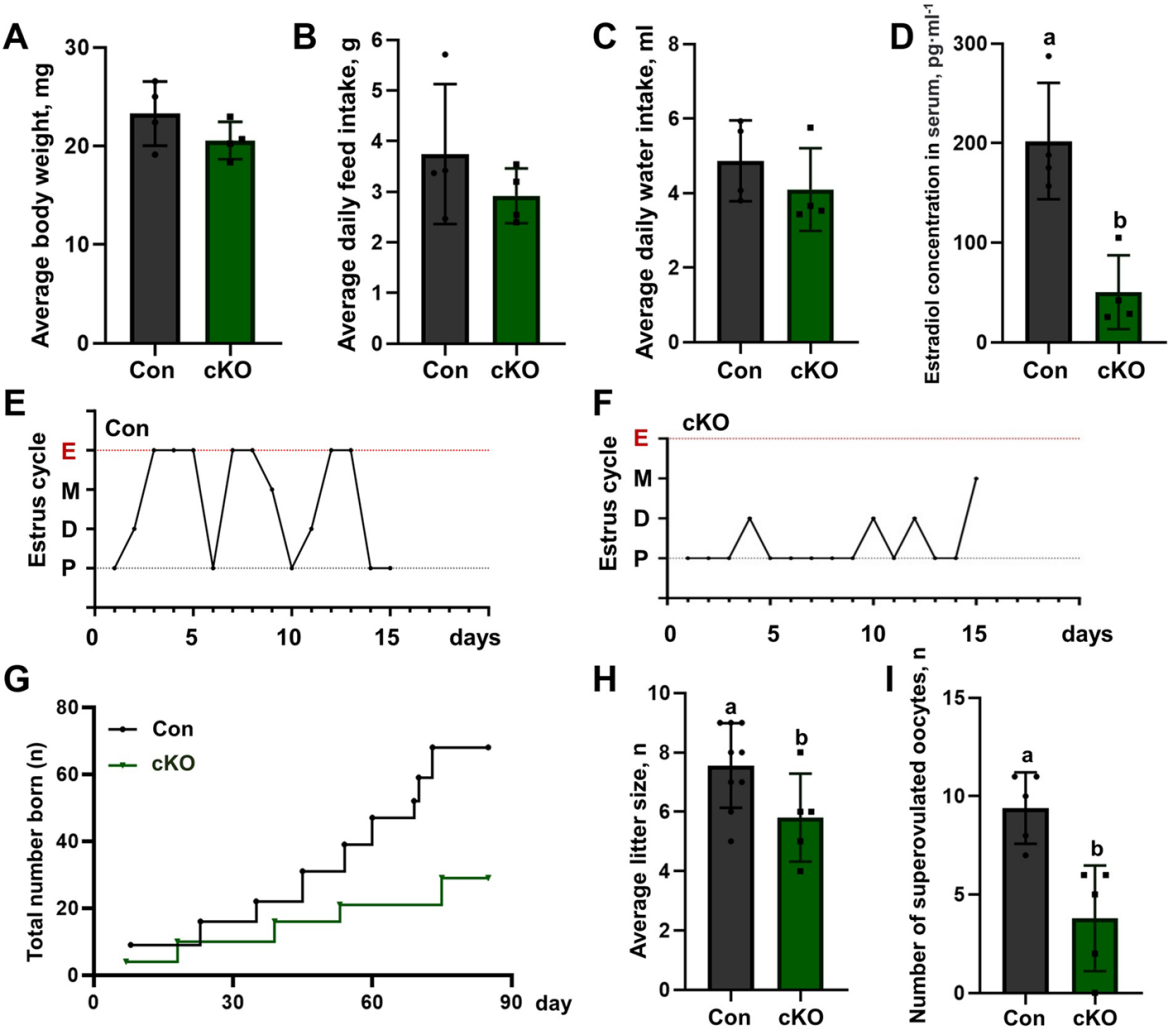
Ovarian GCs CK1α deletion disturbs the estrus cycle and reduces the ovulation rate and litter size. **A-C** Statistics of body weight, feed and water intake of control and cKO mice.13 control and 14 cKO mice were calculated. **D** Serum estrogen levels of mature mice in proestrus determined by ELISA. Each group has five biological replicates. **E**, **F** Representative estrous cycle patterns of control and cKO mice. E, estrus; M, metestrus; D, diestrus; and P, proestrus, each group has four biological replicates. **G** Total litter size of four control and four cKO mice during the three months are displayed by using line charts. **H** The number of neonatal mice per litter.** I** An average number of oocytes obtained from a single mouse with the superovulation method. Each group has five biological replicates. Results are presented as means ± SD, different letters represent the significant difference (*P* < 0.05)

To determine the effect of ovarian GCs CK1α reduction on fertility, we conducted an animal breeding assay. Adult cKO and littermate control female mice (age of 10–12 weeks) were paired with wild-type C57BL/6 males (16 weeks) of known fertility and mating behaviors. Four mice with similar body weights were selected for the research object in each group, feed intake, water intake, and litter number were recorded (Additional files 5 and 6: Table S1, S2). The results showed that there was no difference in the body weight, feed and water intake of cKO mice control mice (Fig. 2A-C). The serum estradiol (E_2_) of proestrus was detected by ELISA (enzyme-linked immunosorbent assay). The results showed that the serum E_2_ levels declined by 75.1% as compared with the control groups (*P* < 0.05) (Fig. 2D).

Besides, the estrous cycles of the female mice in the control group regularly alternated (Fig. 2E), while the estrous cycles of the cKO mice were disordered (Fig. 2F). Sixty-eight offspring were produced in the control group; however, this number was lower (*P* < 0.05) (*i.e.*, one-third of the control group) during the recording period in the cKO group (Fig. 2G); the average litter size of control groups was 7.6 ± 0.47, and that of cKO was 5.8 ± 0.66 (Fig. 2H). After intraperitoneal injection of PMSG for 48 h, the oocytes from the ampulla of the oviduct were collected. The number of superovulation oocytes in female cKO mice was reduced (*P* < 0.05) (Fig. 2I). Besides, the calculated results showed that the plugged rate and pregnancy rate were declining in the cKO group (Additional file 7: Table S3). These results revealed that ovarian GCs CK1α reduction disturbs the estrus cycle and reduces serum E_2_ levels and fertility.

### CK1α reduction in mouse ovarian GCs leads to follicular atresia and GCs apoptosis

**Fig. 3:**
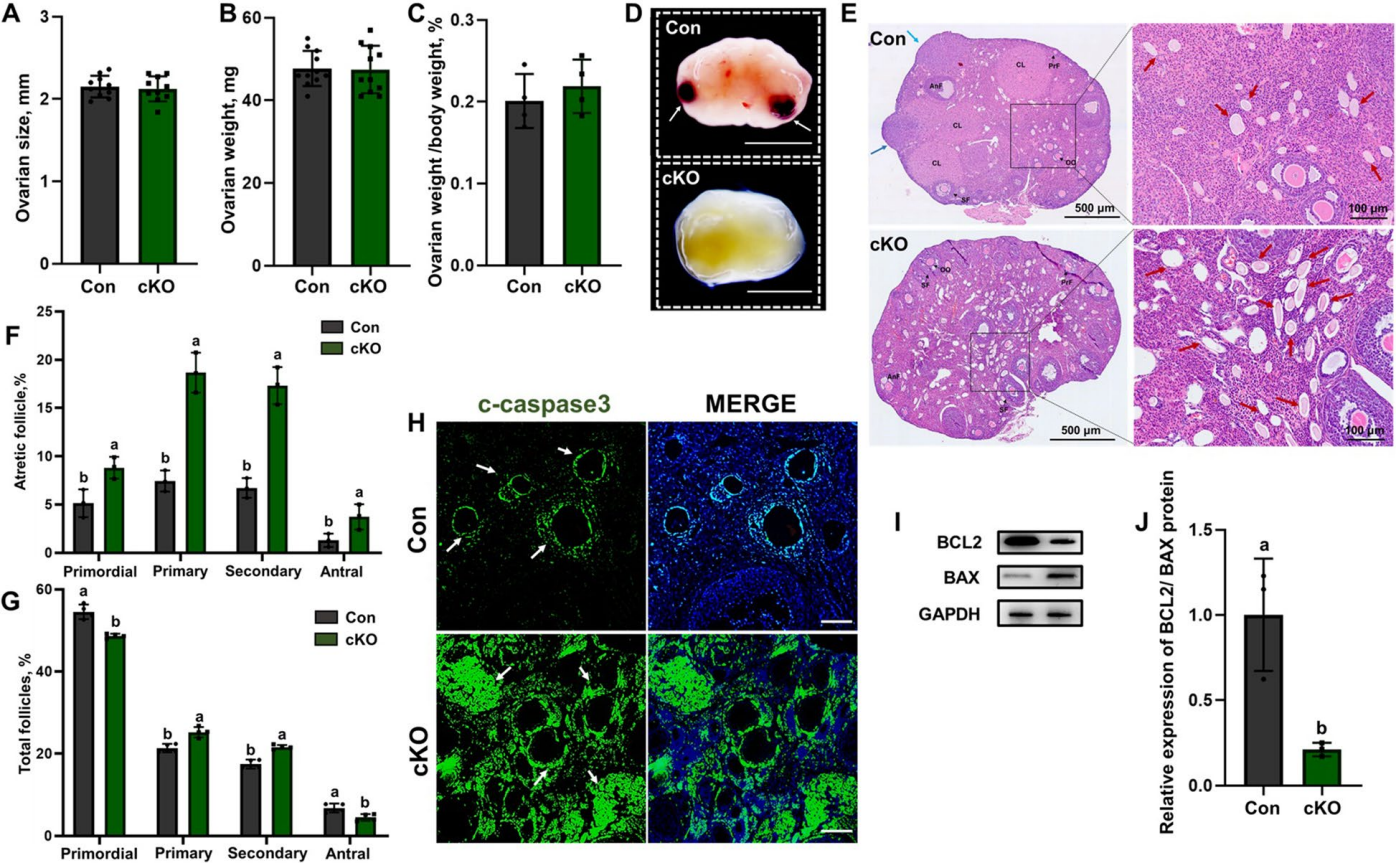
Ovarian GCs CK1α deletion causes follicular atresia and increases GCs apoptosis. **A**, **B** The size and weight of eight-week-old control and cKO mice ovarian tissues. At least 10 biological replicates were performed. **C** Organ coefficients of ovarian tissues, each group has four biological replicates. **D** The morphology of ovarian tissues in estrus was observed under a magnification microscope; white arrows represent mature follicles. Scale bar = 1 mm. **E** HE staining of ovary sections from control and cKO mice; red arrow represents atretic follicle. Scale bar = 500 µm. **F**, **G** Statistical analysis showed the number and follicular atresia of different types of follicles in the control mice and cKO mice ovaries. Each group has three biological replicates. **H** Representative images of ovary sections IF staining. C-caspase 3 is shown in green; DAPI-stained nuclei are shown in blue. Scale bar: 100 µm. **I** Western blot analysis of relative BCL2 and BAX protein levels of mouse ovaries. The gray values of BCL2 and BAX acquired by ImageJ software were normalized to GAPDH. Each group has three biological replicates, results are presented as means ± SD and different letters (a and b) indicate significant differences (*P* < 0.05)

Some studies have suggested a positive correlation between ovulation rate and litter size [32, 33] and that GCs influence the follicular atresia of the ovary [34, 35]. We isolated the ovarian tissues from mouse and there are no other abnormal changes about ovarian size, weight and organ coefficient (Fig. 3A-C). In the control mice, follicles protruded from the surface of the ovary and formed into erythema (indicated by the white arrow). In cKO mice, however, ovaries were white and had no prominent follicles on the ovarian surface (Fig. 3D). After observing the morphology of ovaries, HE staining was performed to determine the physiology changes that had occurred. A large number of non-maturating follicles developed into the atretic follicle in the ovarian follicles of cKO mice (Fig. 3E). Then, the primordial follicle, primary follicle, secondary follicle, antral follicle, and atretic follicle of eight-week-old mice were counted, the atresia rate of follicles at different stages was increased and the proportion of antral follicles was decreased in cKO mice (all *P* < 0.05) (Fig. 3F, G). These demonstrated that ovarian GCs CK1α reduction might lead to granulosa cell apoptosis and oocyte atresia, thus affecting ovulation, estrus cycle, implantation, and E_2_ hormone secretion. Next, we analyzed the apoptosis-related factors in the cKO mice ovary by IF staining and WB. The results showed that the apoptotic signal caspase-3 of cKO mice ovary was higher than that of the control group (Fig. 3H), the anti-apoptotic protein BCL2 was reduced, while the expression of proapoptotic protein BAX was increased in ovarian tissue (all *P* < 0.05) (Fig. 3I, J).

### CK1α mediates estradiol synthesis by inhibiting estradiol-synthesizing enzyme CYP19A1 through MAPK signaling pathway in the mouse ovary

**Fig. 4:**
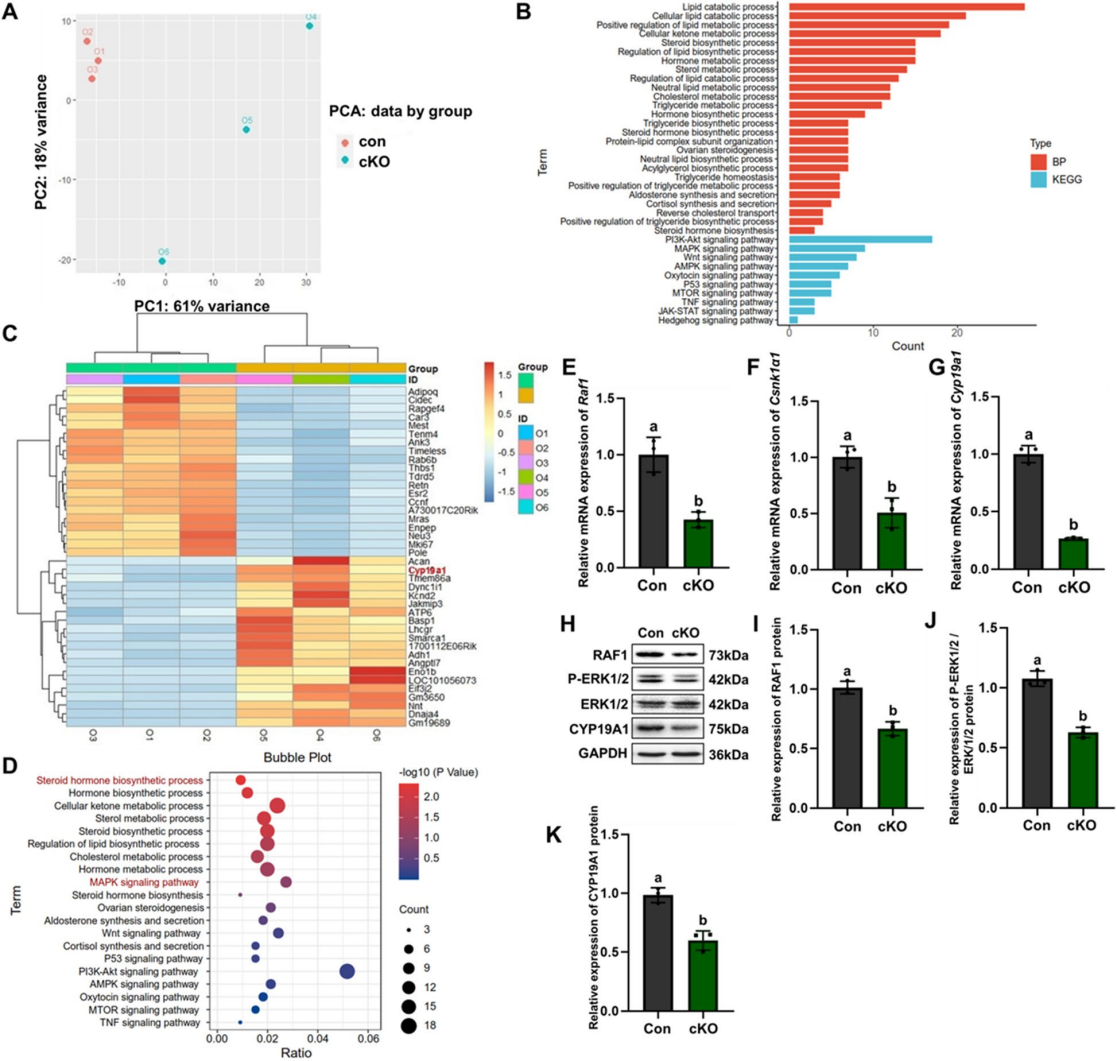
Ovarian GCs CK1α deletion decreased gene expression in Cyp19a1 and altered the functional pathways in steroid hormone synthesis and MAPK signaling pathway. **A** PCA is used in control and cKO female mice ovaries RNA-seq data statistical features extracting. Red dot, control mice data. Cyan dot, cKO mice data. **B** The GO and KEGG Pathway enrichment analysis were carried in control and cKO ovarian tissues. **C** Heat map showing differentially expressed genes in control and cKO groups; the top 20 genes with *P* < 0.05. **D** Functional enrichment using GO and KEGG Pathway analysis with the genes in differential expression in control and cKO ovaries. **E–G** Real time RT-PCR validation of three important genes in ovarian tissues (*Raf1*, *Csnk1α1*, *Cyp19a1*). **H** Relative expression of RAF1, P-ERK1/2, ERK1/2, and CYP19A1 detected by WB. GAPDH was used as an internal control. **I-K** Protein expression data were normalized to GAPDH and quantified using ImageJ software. Different letters indicate that the difference is significant (*P* < 0.05). Results are presented as means ± SD and each group has three biological replicates

To explore the effect of CK1α granulosis-specific knockdown in estrogen secretion, RNA sequencing (RNA-seq) was performed in ovaries to screen out the pathways related to hormone production by kyoto encyclopedia of genes and genomes (KEGG)) and gene ontology (GO) enrichment analysis. Principal component analysis (PCA) scatter plots of transcriptome samples were drawn by the dimensionality reduction method. The control group is concentrated in the upper left region with small intra-group differences, while the knockout group is concentrated in the lower right region with a discrete distribution. The results showed that the cKO group was separated from the control group (Fig. 4A). Gene-set enrichment analysis results showed the differentially expressed genes were in metabolic pathways related to hormone biosynthesis (Fig. 4B). Specifically, the heatmap results showed that the *Cyp19a1* gene (estrogen synthetase) was downregulated (Fig. 4C). Furthermore, GO and KEGG pathway analyses revealed that the steroid biosynthetic process, sterol metabolic process, and MAPK signaling pathways were largely affected in the cKO ovarian tissue (Fig. 4D). These results suggested that the CK1α granulosis-specific knockdown could decrease *Cyp19a1* gene expression and the E_2_ synthesis associated with MAPK signaling pathways. QRT-PCR was used to verify the RNA seq results, and the results showed that the mRNA expression of *Raf1*, *Csnk1α1* and *Cyp19a1* genes in the ovaries of cKO mice were decreased (all *P* < 0.05) (Fig. 4E-G). CK1α was an important regulatory factor in the canonical WNT signaling pathway. The transcriptomic results showed that *Wnt10b*, *Sfrp2*, and other WNT signaling pathway-related genes increased in the cKO mice ovary. Western blot assays of the protein expression levels of FSHR, CK1α, RAF1, P-ERK, ERK, CYP19A1, Frizzled4, LPR6, and β-catenin were performed to find out whether CK1α granule-cell knockout affected the WNT signaling pathway. The reduction of CK1α blocked the RAF1, P-ERK1/2, and CYP19A1 protein expression (Fig. 4H-K) but did not affect the protein expression of Frizzled4, LPR6, and β-Catenin (Additional file 8: Figure S5). These in vivo results suggested that CK1α mediates estradiol secretion by CYP19A1 through the MAPK signaling pathway rather than the WNT signaling pathway.

### CK1α regulates the synthesis of estrogen through the MAPK-CYP19A1 signaling pathway in primary GCs of cKO mice

**Fig. 5:**
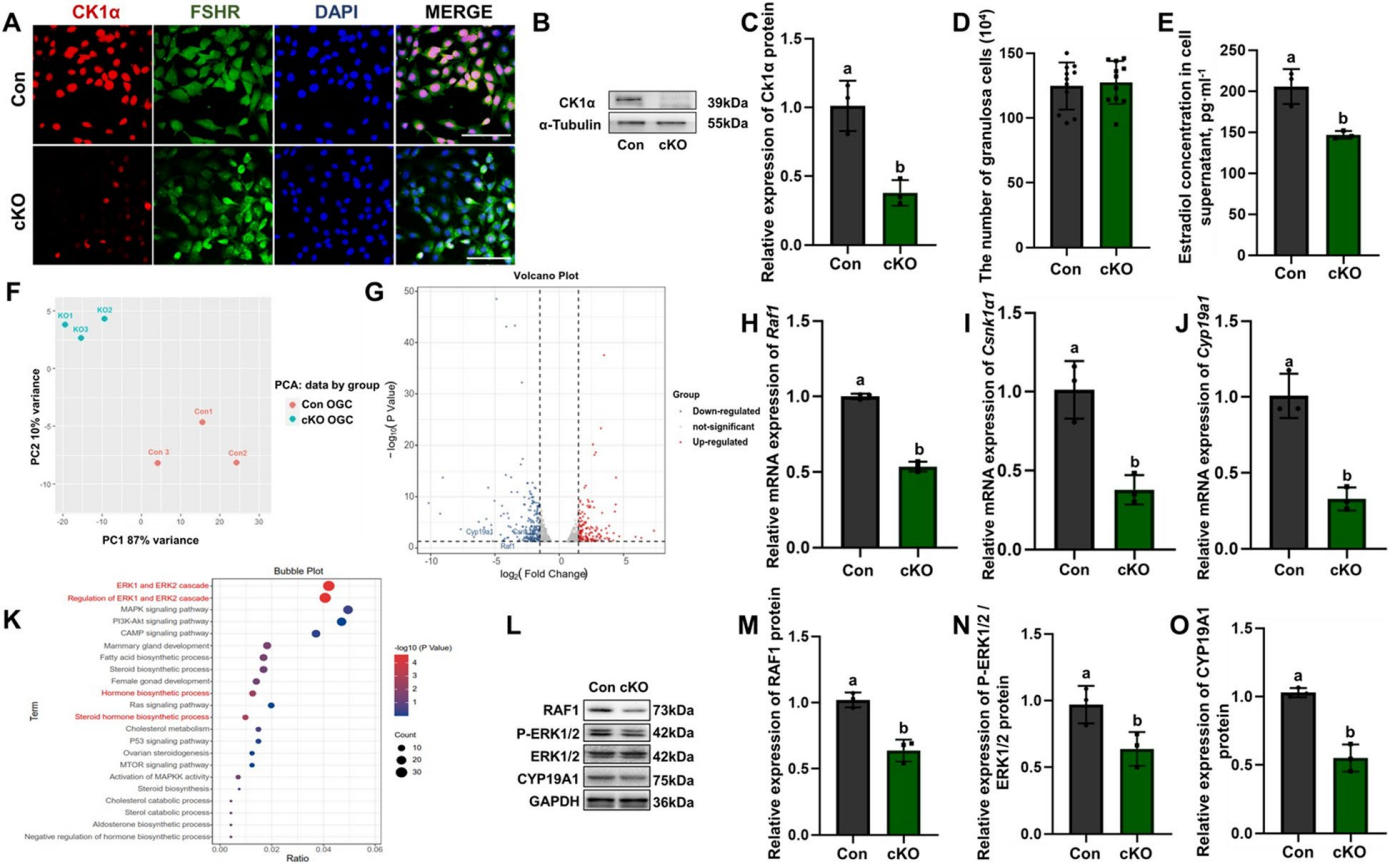
CK1α regulates estrogen synthesis through the MAPK signaling pathway in primary GCs of cKO mice. **A** Immunofluorescent double staining for the expression of FSHR (ovarian GCs marker; green) and CK1α (red) in primary GCs from control and cKO mice ovaries. DAPI (blue) was used to stain the nucleus. Scale bar = 30 µm. **B**, **C** Representative image of WB detecting the knockdown efficiency of CK1α protein inside primary GCs. Relative protein levels were normalized to α-Tubulin and quantified by using ImageJ software. **D** The quantity of primary ovarian granulosa cells in cell culture dishes were counted. **E** E_2_ content in culture media was measured by ELISA. **F** PCA is used in control and cKO female mice primary ovarian granulosa cells RNA-seq data statistical features extracting. Red dot, control group data. Cyan dot, cKO group data. **G** Differentially expressed genes were analysis by using volcano map. Blue dots represent genes that are down-regulated, red dots represent genes that are up-regulated, and gray dots represent genes that are not significantly different (*P* < 0.05, | log2 fold change |> 1.5). **H-J** Real time RT-PCR validation of three important genes in primary ovarian granulosa cells (*Raf1*, *Csnk1α1*, *Cyp19a1*). **K** Functional enrichment using GO and KEGG Pathway analysis with the genes in differential expression in primary ovarian granulosa cells. **L** Relative expression of RAF1, P-ERK1/2, ERK1/2, and CYP19A1 detected by WB. GAPDH was used as an internal control. **M–O** Protein expression data were normalized to GAPDH and quantified by using ImageJ software. Results are presented as means ± SD and each group has three biological replicates. Different letters (a and b) represent the significant difference (*P* < 0.05)

The primary GCs were isolated from the cKO mice, and the *Csnk1a1* deletion efficiency in primary GCs was assessed using IF staining and WB. The results showed that the CK1α positive signals were decreased in the cKO mice ovarian GCs (Fig. 5A), and the CK1α protein levels were lower than those of the control group (*P* < 0.05) (Fig. 5B, C). Cell numbers were counted before estradiol measurement (Fig. 5D). Bright field of cells was provided in supplementary materials. Next, ELISA was performed. Compared with the control group, the E_2_ level in the primary GCs supernatant of the cKO was reduced.

RNA-seq was performed in primary GCs to verify the results in vivo. The results showed that the two groups were separated (Fig. 5E), and the *Raf1*, *Csnk1α1* and *Cyp19a1* genes were diminished (all *P* < 0.05) in primary GCs of cKO mice (Fig. 5G). The results of mRNA expression of *Raf1*, *Csnk1α1* and *Cyp19a1* were verified by qRT-PCR (Fig. 5H-J). KEGG and GO enrichment analysis results showed that the regulation of ERK1 and ERK2 cascade, steroid hormone biosynthetic process metabolic pathways were largely affected in the cKO primary GCs (Fig. 5K). Meanwhile, the changes in the MAPK-CYP19A1 signaling pathway were verified using WB. The expression levels of RAF1, P-ERK/1/2, and CYP19A1 protein in primary GCs of cKO mice decreased (all *P* < 0.05) (Fig. 5L-O). These results are consistent with in vivo data and further suggested that ovarian GCs CK1α reduction influences MAPK-CYP19A1 regulated signaling pathway.

### CK1α is involved in the signal transduction pathways by binding to RAF1

**Fig. 6:**
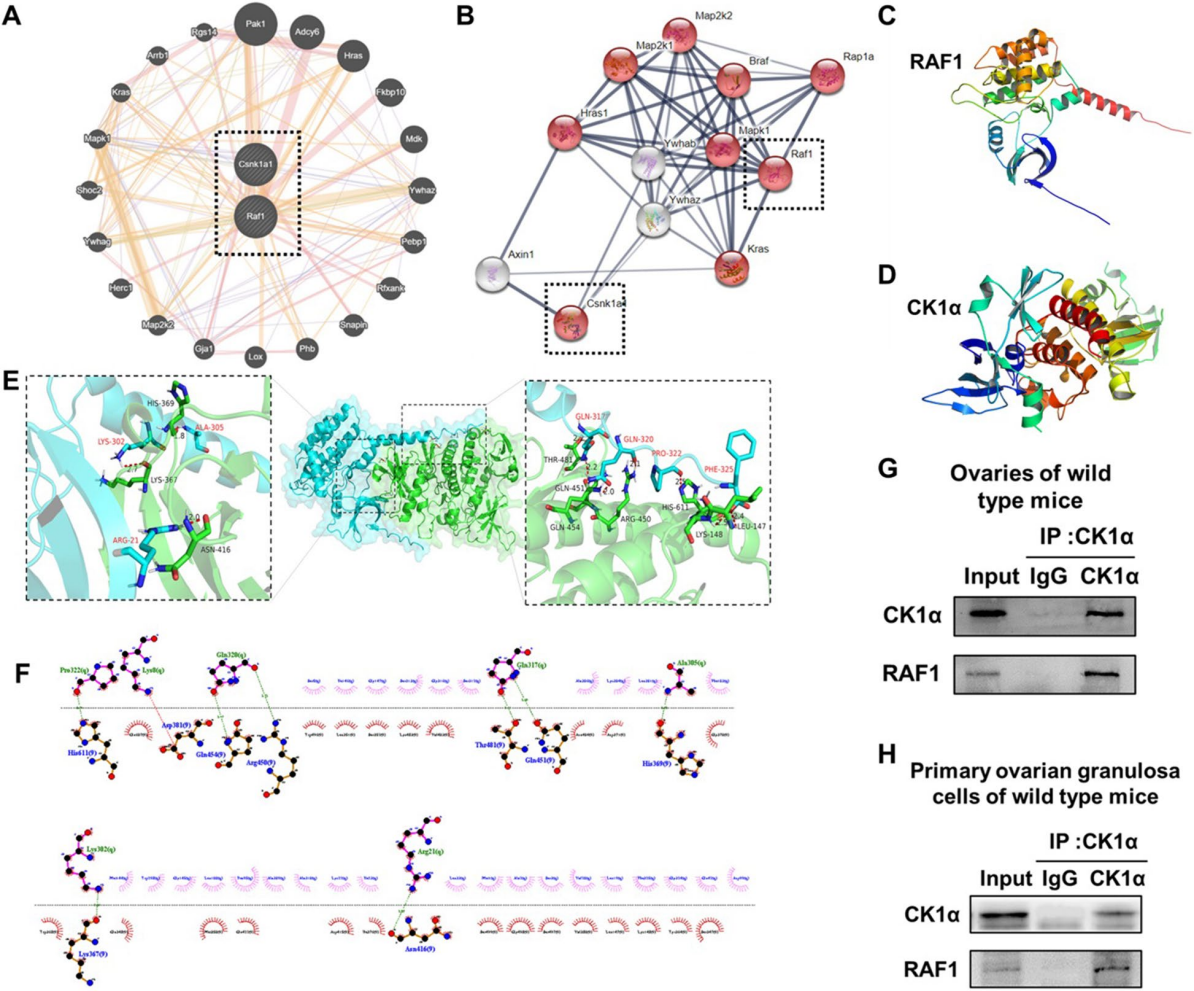
(A-D) CK1α involved in the signal transduction pathways by binding with RAF1. **A**, **B** Interlinkage between CK1α and RAF1 encoding genes were predicted by using Stitch and Genemania websites. **C**, **D** Structure of RAF1 and CK1α proteins in PyMOL. **E** Structure of CK1α bound to RAF1. RAF1 as ligand–protein is indicated in cyan, and CK1α as receptor protein was shown in green. **F** CK1α- RAF1 interaction plot. CK1α residues are presented in pink above the dashed line, and RAF1 residues are shown in red beneath the dashed line. Hydrogen bonds are shown as green dashed lines, ion pairs as red dashed lines, and the value above the dotted line represents the distance. The images were constructed using Discovery Studio (DS) 2016 and Ligplot + v.2.2. **G-H** CK1α is involved in the signal transduction pathways by binding with RAF1. **G**, **H** The interactions between CK1α and RAF1 were detected with immune co-precipitation and in vivo and in vitro. Results are presented as means ± SD; each group has three biological replicates

Protein–protein interactions within the cell profoundly influence protein function, and we investigated the relation between CK1α and RAF1 in ovarian GCs. The transcription genes of proteins were predicted and analyzed using Stitch and Genemania websites. The *Ywhaz* genes which encoded products belong to 14–3-3 proteins adjacent to *Csnk1α1* (Fig. 6A, B). RAF1 was phosphorylated on both 14–3-3 binding sites pS259 and pS621, and 14–3-3 maintained the closed inactive conformation, upon membrane recruitment by activated RAS, pS259 is dephosphorylated by the corecruited phosphatases and cause the activation of RAF1. The predicted results showed that there might be a strong correlation between CK1α and RAF1. CK1α and RAF1 protein structure prediction was preprocessed by the Swissmode website (https://swissmodel.expasy.org) and Discovery Studio software; water molecules, hydrogenation, and charge were deleted to extract the original structural ligand. The structure of the CK1α and RAF1 proteins was visualized with PyMol software (Fig. 6C, D). Using the Discovery Studio (DS) 2016 ZDOCK program, every possible CK1α-RAF1 binding pose in 3D space was calculated and evaluated, and each pose was scored using an energy-based scoring function. The ZDOCK docking results indicated that CK1α-RAF1 exhibited surface complementarity in the interface area (Fig. 6E). The hydrophobic effect and H-bond interaction of CK1α-RAF1 were analyzed by DS2016 and Ligplot + v.2.2. RAF1 Pro322, Lys8, Gln320, Gln317, Ala305, Lys302, and Arg21 formed H-bonds with CK1α (Fig. 6F). These data suggested that CK1α was involved in the signal transduction pathways by binding with RAF1.

Coimmunoprecipitation was performed to investigate whether CK1α interacts with RAF1, the results indicated that CK1α could bound to RAF1 in ovarian tissues and primary ovarian GCs (Figs. 6G, 7H). Therefore, we speculate that CK1α may be involved in the regulation by binding to RAF1 protein in the ovarian GCs.

## Discussion

CK1α protein with a high homology structure is conservative among different species and is widely expressed in various organelles [19, 20]. CK1α can actively phosphorylate and has a multi-site phosphorylation domain [18, 32]. In addition, CK1α have an important regulatory role in gonadal and embryonic development [33, 34] (Additional file 9: Figure S6A), and we demonstrated that CK1α was localized in the germ cells and expressed in the ovaries during early gonadal development (Additional file 9: Figure S6B,C).

Yet, the exact role of CK1α in germ and somatic cells is still not fully understood. We recently demonstrated that specific knockout of CK1α in spermatogonia leads to male infertility and that CK1α regulates spermatogenesis through the p53-Sox3 signaling pathway [29]. Also, the specific knockout of CK1α in oocytes affects the formation of primordial follicles leading to female infertility. We also found that CK1α has a crucial role in the meiosis of reduced oocytes [30]. In this study, we established a GCs-specific CK1α deficient mouse model to further investigate the function of CK1α in E_2_ synthesis in adult female mice ovaries. Our results confirmed the expression of CK1α in somatic cells in the ovary, which is consistent with the previous study [30]. We also found that the CK1α protein level in the ovaries of 28 dpp (days post-partum) mice was higher than that of other stages (3,7 dpp and 14 dpp), and CK1α expression tended to become stable with the sexual maturity. We then crossed *Amh*-cre mice [35] with *Csnk1a1*-floxp mice [31] to obtain ovarian GCs-specific CK1α cKO mice. In order to explore the specific period of CK1α knockdown in ovarian GCs, deletion efficiency of cKO mice ovaries at different developmental stages (three days, one week, four weeks, and eight weeks) were detected, and found that CK1α was efficiently and specifically knockdown in adult female cKO mice ovaries (eight weeks). The estrous cycles of the cKO mice were disordered. Besides, the fertility of female cKO mice and the average litter size was reduced compared with the control group. Previous studies have suggested a positive correlation between ovulation rate and litter size [32, 33]. Moreover, we found that CK1α reduction decreased ovulation in cKO mice.

Ovarian follicles atresia is a degenerative process of germ cells and somatic cells involving autophagy, apoptosis, autophagy, and heterophagy. During this process, the follicle separates from the zona pellucida, and the connections between the GCs become loose [36]. We discovered that CK1α reduction led to a large number of non-maturating follicles developed into the atretic follicle (AF). Also, compared to control mice, ovarian GCs were loosely arranged in cKO mice (Additional file 10: Figure S7).

E_2_ has an important role in follicular development. The FSH can bind to the FSHR on GCs and stimulate cell proliferation, promoting E_2_ secretion to inhibit cell apoptosis and maintain oocyte development [37]. Compared to control mice, the serum E_2_ levels of proestrus was decreased sharply in cKO mice. A previous study has found that many abnormal phenotypes (including estrus cycle distribution, ovulation rate, and litter size were all sharply reduced) are caused by estrogen deficiency [38–42]. Thus, we hypothesized that CK1α deletion in ovarian GCs may lead to these events. Thus, we further explore the main cause of estrogen reduction. First, the differentially expressed genes in cKO mice ovaries were assessed by transcription sequencing. We found that CK1α reduction increases GCs apoptosis, which is inconsistent with previous studies that reported different results, *i.e.*, GCs apoptosis decreases estrogen secretion [43, 44].

Furthermore, RNA-seq sequencing results showed that cell apoptosis was not the only factor causing estrogen reduction [43, 44]. In this study, the estradiol-synthesizing enzyme was equally critical for estrogen synthesis. Previous studies reported that Raf-ERK signaling mediates steroid hormone synthesis in GCs [45, 46]. Combined with transcriptome results, MAPK signaling pathway-related proteins were detected, and the results showed the expression levels of CK1α, RAF1, and CYP19A1 ovary decreased in cKO mice. Moreover, another study reported that CK1α has a regulatory role in the Wnt/β-catenin signaling pathway [23, 47]; however, our results showed that CK1α GCs knockdown did not influence the Wnt signaling pathway-related proteins.

Next, we by in vitro experiments, we confirmed that estrogen synthase in GCs was regulated through CK1α-RAF1. Our RNA-seq results revealed that the *Raf1*, *Csnk1α1* and *Cyp19a1* genes were decreased, and regulation of ERK1 and ERK2 cascade, steroid hormone biosynthetic process metabolic pathways were largely affected in primary GCs of cKO mice. We found that the reduction in the E_2_ level in the cell supernatant led to a decrease in the protein levels of RAF1 and CYP19A1 in the primary ovarian GCs of the cKO mice, which further confirmed the validity of our in vivo data.

A previous study showed that knocking out ERK1 and 2 in GCs elevated the concentration of estradiol in the serum [48]. However, our results revealed that the reduction in the P-ERK/ ERK expression decreased the estrogen secretion in GCs. Therefore, the estrogen synthesis in GCs might be affected by development period of the ovaries or other processes. Besides, abnormal apoptosis was present in cKO ovarian GCs (Additional file 11: Figure S8), and it was unclear whether the experiments ruled out other apoptosis factors in estrogen synthesis reduction. Thus, we replicated experiments in vitro by isolating the wild-type mouse ovarian GCs. The regulatory function of CK1α was validated again by adding CK1α inhibitor D4476. However, the results showed that the effect of apoptosis remains, the inhibition of CK1α might mainly by means of inducing the apoptosis of ovarian GCs (Additional file 12: Figure S9).

Finally, prediction results suggested that CK1α coexists with various biochemical activities related to RAF1 signal transduction and that the proteins exist through intermolecular binding. Furthermore, the ZDOCK docking results indicated that CK1α-RAF1 exhibited surface complementarity in the interface area. The hydrophobic effect and H-bond interaction of CK1α-RAF1 were analyzed by DS2016 and Ligplot + v.2.2. The site of RAF1, including Pro322, Lys8, Gln320, Gln317, Ala305, Lys302, and Arg21 formed H-bonds with CK1α. These data suggested that CK1α is involved in the signal transduction pathways by binding with RAF1. To verify relationships in the regulatory function signaling pathway, we detected CK1α by IF double-staining and coimmunoprecipitation in vitro and in vivo, and results showed that CK1α could bound to RAF1 and co-localized in ovarian tissues and primary ovarian GCs, as previously reported [45]. In vivo and in vitro experimental results revealed that CK1α may be involved in the regulation of estradiol synthesis by binding to RAF1 protein in ovarian tissues and primary ovarian GCs. ER knockouts (ERalphaKO, ERbetaKO, and ERalphabetaKO) share the same reproductive phenotype with CK1⍺cKO mice that infertile or exhibit variable degrees of subfertility and the vast majority females do not ovulate [49]. Although they have similar phenotype, they work on estrogen synthesis through different mechanisms. ER plays an important role in mediating the estrogens by cell quantity (cell proliferation), while CK1α regulates the estrogen synthase by cell function (Estrogen synthesis). This study has some limitations. CK1α knockout in pregranulosa cells whether led to oocyte atresia, or affect primordial follicle formation need to be further studied by *Foxl2*-Cre tool mice, and the oocyte-granulosa cell signaling communication needs to be further explored.

## Conclusions

CK1α regulates the expression of estrogen synthase CYP19A1 and estradiol synthesis by binding to RAF1 protein. Our present findings provide novel insights into the regulation mechanism of female reproduction and estrogen synthesis.

## Methods

### Animals and treatments

A total number of 36 C57BL/6 female wild-type mice were purchased from Beijing HFK Bioscience Co. Ltd (Beijing, China). In addition, four *Csnk1α1* floxed mice (*Csnk1a1*^*flox/flox*^) (Stock #025398)[31] and two *Amh*-*Cre* transgenic mice (Stock #033376) were purchased from the Jackson Laboratory (Bar Harbor, ME, USA) and bred in the laboratory. All the animals were housed in an environment with a temperature of 22 ± 2 ºC, relative humidity of 60%–65%, and a light/dark cycle of 12/12 h and were given basal diet and pure water.

The mice were anesthetized using pentobarbital sodium and subsequently euthanized by cervical vertebral fracture, and the uterus, ovaries, and oviducts were collected and stored at -80 ^○^C for protein detection or fixed in 4% paraformaldehyde (PFA) diluted in phosphate-buffered saline (PBS, pH7.4) for immunohistochemistry (IHC). In addition, the blood serum was collected to measure estrogen levels.

### Fertility test

Sexually mature females of *Csnk1α1* knockout mice (Cre ± ; loxp + / + or cKO) and controls (Cre-/-; loxp + / + or Con) (10–12 weeks) were used for the fertility test. Female mice were mated with wild-type C57BL/6 males (16 weeks) for three months. Female vaginal plugs were checked every morning before pregnancy, and the number of pups in each cage was recorded for each group.

### Determination of each phase of the estrous cycle

The phase of each estrous cycle was determined by the examination of vaginal smears stained with Wright's dye (Solarbio Life Sciences, Beijing, China). Estrous cycle phases were defined as follows [50, 51]: proestrus (100% intact epithelial cells), estrus (100% cornified epithelial cells), metestrus (∼50% cornified epithelial cells or exfoliated epithelial cells and 50% leukocytes), and diestrus (cell debris, some cornified epithelial cells or leukocytes).

### Isolation and culture of primary ovarian GCs

Mice were given pregnant mare serum gonadotropin (PMSG,10 IU). Ovarian primary granulosa cells (GCs) were isolated and collected by follicle puncture as previously described [52]. Primary ovarian GCs were incubated in Dulbecco’s Modified Eagle Medium/Nutrient Mixture F-12 (DMEM/F12) containing 10% fetal bovine serum (FBS) supplemented by 100 U/mL penicillin and 100 mg/mL streptomycin, in a 5% CO_2_ incubator with saturated humidity and a constant temperature of 37 ^○^C.

### In vitro experiments

In vitro experiments were divided into three stages. E_2_ content and protein detection were measured after each step.

1. Primary ovarian granulosa cells were isolated from mice (Fig. 5), and inoculated in 6-well plates (1 × 10^7^/well) for 12 h. Then, the cells were trypsinized, and hormone levels in the supernatant were analyzed.
2. Twenty adult WT were selected for this experiment. Primary ovarian GCs from the WT mice were collected and seeded in a 6-well plate at a density of 1 × 10^6^ cells in a standard culture medium. After attaching to the bottom of the dish, the culture medium was replaced with a medium without FBS, and cells were cultured 6 h. D4476 was dissolved in DMSO and further diluted in complete medium and cells were then treated with D4476 (50 μM) for an additional 6 h; the equivalent volume of DMSO was used as D4476 vehicle control.
3. Ten adult WT were selected for this experiment. Primary ovarian GCs from the WT mice were collected and seeded in a 6-well plate at a density of 1 × 10^6^ cells in a standard culture medium. After attaching to the bottom of the dish, the culture medium was replaced with a medium without FBS, and cells were cultured 6 h. Cells were then treated with RAF1 inhibitor RAF709 (5 nM) for 6 h as previously reported [45].

### Real-time quantitative PCR (RT-qPCR)

The tissue and cell samples were dissected and extracted by grinding under liquid nitrogen. Total RNA was extracted from the tissue and Trizol reagent (TaKaRa Biotechnology, Dalian, China) following the manufacturer’s instructions. Purified RNA (1 µg) was used as a template for cDNA synthesis. Samples were mixed with Oligo (dT) at 72 ^○^C for 5 min, cooled to 0–4 ^○^C for 5 min, and then mixed with MLV reverse transcriptase, dNTP, and RNA safe (Promega, Madison, WI, USA) for 1 h at 42 ^○^C. RT-qPCR was performed using a standard Takara SYBR Premix Ex Taq protocol (Vazyme Biotech Co., Ltd, Nanjing, China) on an Applied Biosystems 7500 Real-Time PCR system (Applied Biosystems; Thermo Fisher Scientific Corp., Waltham, MA, USA). The mixture was heated to 95 ^○^C for 10 min, followed by 40 cycles of 95 ^○^C for 15 s and 60 ^○^C for 1 min. Glyceraldehyde-3-phosphate dehydrogenase (GAPDH) expression levels were used for data normalization, and the relative abundance of genes was determined using ABI PRISM 7500 equipped software (©2009–2017, Analytik, Jena AG). The relative product levels were quantified using the 2^–∆∆Ct^ method; the primers for RT-qPCR analyses are presented in Table 1 (Additional file 13). Genotyping identification of mice was performed by PCR using primer sets published on the Jackson Online website at https://www.jax.org/strain/025398 and https://www.jax.org/strain/033376.

### Western Blotting (WB)

Ovaries and GCs (stage 1, 2, and 3; see in vitro experiments) were lysed with RIPA buffer (C1053, Applygen, Beijing, China). The protein concentration of each sample was quantitated by the BCA assay reagent (HX18651, Huaxingbio, Beijing, China). Samples were electrophoresed on SDS-PAGE and transferred to polyvinylidene fluoride (PVDF) membrane (IPVH00010, Millipore, Billerica, MA, USA). The membrane was soaked in methanol, and the target proteins were transferred to a PVDF membrane and incubated with CK1α antibody (1:2000, ab64939, Abcam), RAF1 antibody (1:2000, ab137435, Abcam), CYP19A1 antibody (1:1000, BA3704, Boster), P-ERK1/2 antibody (1:1000, 4370T, Cell Signaling Technologies), ERK1/2 (1:1,000, A4782, ABclonal), Frizzled (1:1000, M003762, Abmart), LPR6 (1:1000, M026228, Abmart), β-catenin (1:1000, 8480, Cell Signaling Technologies), GAPDH (1:2000, PA5-85074, Ambion) and α-tubulin antibody (1:1000, T40103, Abmart) at 4 ^○^C overnight. The membrane was then washed in TBST (0.1% Tween-20 in TBS, Sigma-Aldrich, P1379) and incubated with horseradish peroxidase (HRP)-conjugated goat anti-mouse IgG (1:4000,115–035-062, Jackson Immuno Research)or HRP conjugated goat anti-rabbit IgG (1:4000, 111–035-003, Jackson Immuno Research) for 2 h at room temperature. After 30 min in TBST, the membrane was treated with ECL Western blotting substrate (32209; Thermo Scientific, Waltham, MA, USA) at room temperature and assessed using the ImageJ software (1.4.3, ©1993–2006, Wayne Rasband), GAPDH and α-tubulin was used as the endogenous control.

### Immunohistochemical analysis

Tissue sections in paraffin were dewaxed in ethanol and soaked in 3% H_2_O_2_ (vol/vol) for 20 min to eliminate endogenous peroxidase activity. Samples were microwaved in 0.01 M sodium citrate buffer on high power for 15 min, washed with PBS, and then incubated with normal goat serum (10%) for 1 h at room temperature to eliminate background nonspecific coloring. The sections were then incubated with CK1α antibody (1:200, ab64939, Abcam) overnight at 4^○^C and biotinylated goat anti-rabbit IgG (1:200; 11–065-14, Jackson) was incubated for 2 h at 37 ^○^C. HRP-conjugated streptavidin (1:200; 016–030-084, Jackson) was used for incubating at room temperature, and diaminobenzidine was added for color rendering, with the color development degree controlled under the microscope. The appearance of brown staining was considered a positive reaction, which was analyzed using VENTANA Image Viewer (©2017, Ventana Medical Systems, Inc).

### Immunofluorescence (IF) assay

After reaching 85% confluence, GCs grown on glass slides were fixed in 4% paraformaldehyde (PFA) for 15 min and then treated with cold methanol for 15 min. GCs and tissue sections were then washed with PBS for 10 min, permeabilized with 0.1% Triton X-100 (Sigma-Aldrich, T8787), then blocked using 10% normal goat serum in TBS for 1 h at room temperature. The slides were incubated with RAF1 antibody (1:150; HY-100510, Abcam), FSH receptor (FSHR) antibody (1:100, sc-13935, Santa Cruz), DDX4 antibody (1:100, ab13840, Abcam), cleaved caspase-3 (1:200, 9664S, Cell Signaling Technologies), FOXL2 antibody (1:200, 19,672–1-AP, Proteintech) overnight at 4 ^○^C. Then, the slides were washed with PBS and incubated for 2 h at room temperature with the FITC-labeled goat anti-rabbit IgG (GAR-FITC, ZF-0311, Zhongshan) for 50 min. Then the nuclei were dyed with 4',6-diamidino-2-phenylindole (DAPI, 1:1,000, D8417; Sigma) for 10 min. Nonimmune rabbit IgG was used as a negative control. The signals were collected by using a fluorescence microscope photograph system (Leica Microsystems, Buffalo Grove, USA).

### ELISA

E_2_ content from blood serum mice in proestrus and culture liquid supernatant from GCs were determined (n = 3 per experimental group) using an ELISA kit (Quanzhou Ruixin Biological Technology Co., Ltd, Quanzhou, China), following the manufacturer’s instructions. Briefly, blood was collected from mouse eyeballs, after which the serum was separated by centrifugation. For primary cell supernatant, 1 × 10^6^ cells were grown in a 6-well plate, after which the cell supernatant was collected when the cell density was approximately 70% (24 h after cell walling).

### Oocyte collection

Four Control mice and four cKO mice aged 10–12 weeks (four mice with similar body weight were selected for the research object in each group) were superovulated with 10 IU (intraperitoneal) pregnant mare serum gonadotropin (PMSG; Ningbo Hormone Products Co., Ltd, Ningbo, China). After 48 h, 10 IU of human chorionic gonadotropin (hCG; Ningbo Hormone Products Co., Ltd, Ningbo, China) was injected. Thirteen h after the hCG injection, cumulus-oocyte complexes were recovered in an M2 medium supplemented with 4 mg/mL bovine serum albumin from the ampulla of the oviducts. Cumulus cells were removed from oocytes with hyaluronidase (300 IU/mL) for 5 min in the M2 medium. Only oocytes with normal morphology were used for statistics and IF assay.

### Flow cytometry

For apoptosis detection, primary granulosa GCs (adherent and floating) were harvested and analyzed according to the manufacturer’s protocol (Beijing Biosea, Biotechnology, Co., Ltd, Beijing, China). Briefly, 1–5 × 10^5^ GCs were resuspended in 500 µL of 1X Annexin V binding buffer and then in annexin V-FITC (5 µL) and propidium iodide (5 µL) for 5 min in the dark. Early and late cell apoptosis were analyzed by flow cytometer (BD, Biosciences, San Jose, CA, USA).

The maximum absorption and emission wavelengths of FITC were 490 and 525 nm, while those of PI-DNA complexes were 535 and 615 nm. Two control groups (PI staining and Annexin V-FITC staining) were set up. Data analysis was performed using the FlowJo software (14.0.0, ©2012, Flexera Software LLC), following the manufacturer’s instructions. FITC on the abscissa and PI on the ordinate. Live cells (Annexin V-/PI-), early apoptotic cells (Annexin V + /PI-), late apoptotic cells (Annexin V + /PI +).

### Coimmunoprecipitation assay

Ovarian tissues and primary ovarian GCs were collected in precooled NP40 buffer. Coimmunoprecipitation assay was performed using protein A/G agarose beads (Santa Cruz Biotechnology, Santa Cruz, CA, USA) according to the manufacturer's instructions. Briefly, 500 μg sample was incubated with 2 μg of anti-CK1α antibody or anti-IgG antibody for 2 h, followed by incubation with 20 μL of protein A/G agarose beads overnight at 4 ^○^C. The samples were washed 5 times with precooled NP40 buffer, boiled for 5 min and analyzed via WB.

### Transcriptome profiling

A total number of six ovarian tissue samples (three ovaries for each group) were randomly selected from the control and cKO groups. Total RNA was isolated and purified using TRIzol reagent (Invitrogen, Carlsbad, CA, USA) following the manufacturer's procedure. The RNA amount and purity of each sample were quantified using NanoDrop (ND-1000, Wilmington, DE, USA). The mRNA was reverse transcribed into first-strand cDNA using the Super Script III reverse transcriptase kit (Invitrogen, 18,080–085), followed by second-strand cDNA synthesis using the Second Strand cDNA synthesis kit (Bio Wavelet, BWR001). The cDNA library preparation was performed as described previously [53]. The average insert size for the final cDNA library was 300 bp (± 50 bp), and all libraries were sequenced using the Illumina HiSeq platform [54].

### Identification of differentially expressed genes and gene functional enrichment analyses

Genes with an adjusted *P*-value (Q-value) < 0.01 and an absolute value of fold change > 1.5 were considered differentially expressed genes. The threshold for the significantly enriched gene sets was here set as *P*-value < 0.05. The gene expression signatures and relevant biological information were performed by online tools such as https://www.omicstudio.cn/tool and https://hiplot.com.cn.

### Computation protein–protein docking

Docking of CK1α (PDB ID: 5fqd) with RAF1 (PDB ID: 6kkn) was performed using ZDOCK and RDOCK from Discovery Studio 2016 [55]. The ZRANK algorithm ranked the docking poses, and the best-docked pose was selected based on the evaluation of binding interface residue. The top-ranked structures were further visualized by Discovery Studio (DS) and Ligplot + v.2.2.

### Statistical analysis

All experimental data were processed and analyzed by GraphPad Prism software (8.0.2, ©1992–2019, GraphPad Software Inc., San Diego, CA, USA) and SAS software (©2001, Indigo Rose, Corporation). All quantitative data are presented as means ± standard deviation. One-way analysis of variance (ANOVA) and Duncan’s tests were used to analyze the main effects of treatments, and a *P-*value < 0.05 was considered to indicate statistically significant differences. WB grayscale images were converted to peak and their size was measured by ImageJ software (1.4.3, ©1993–2006, National Institutes of Health, Rockville Pike, Bethesda, MA, USA). Each experiment was repeated at least three times.

### Supplementary Information

Additional file 1: Figure S1. Negative control of CK1α in different tissues. The localization of CK1α in the ovaries, uterus and oviduct of an adult mouse detected by IHC. Paraffin slices of tissues were incubated with CK1α antibodies, and antibodies were replaced with anti-rabbit IgG for negative control. Granular cell, Epithelial cells, Glands, Myometrium. Each tissue was analyzed in three biological replicates. Scale bar = 100 μm

Additional file 2: Figure S2. Expression of CK1α in follicles at different developmental stages. The localization of CK1α proteins of adult mouse ovary was performed by IHC.Primordial follicle: consists of a single oocyte and a single layer of flattened granulosa cells.Primary follicle, the granulosa cells proliferate from a single layer to multiple layers, and a transparent zone appears between the granulosa cells and the oocyte.Secondary follicle with multiple layers of granulosa cells around the oocyte, and the follicular antrum appears in the follicle.Antral follicle, the volume of the follicle is the largest at this time. Selective brown staining revealed CK1α-positive signals. Scale bar = 30 μm

Additional file 3: Figure S3. Deletion efficiency of cKO mice ovaries at different developmental stages. The expression of CK1α was investigated in ovarian tissue of cKO mice from postnatal three days, one week, four weeks, and eight weeks by immunofluorescence staining and WB method.Representative images of ovary sections IF staining. CK1α in red; DAPI stained nuclei in blue. Scale bar = 50 µm.Representative image of WB detecting the knockdown efficiency of CK1α protein inside ovarian GCs in vivo. Relative protein levels were analyzed by gray scanning and normalized to GAPDH. Each tissue was analyzed in three biological replicates

Additional file 4: Figure S4. Expression of CK1α in the uterus and oviduct of adult female mice. The localization of CK1α in the uterusand oviductof an adult mouse detected by IHC. Paraffin slices of uterus and oviduct tissues were incubated with CK1α antibodies. Selectively staining brown demonstrated CK1α-positive signals. Scale bar = 50 μm. Epithelial cells, glands. Each tissue was analyzed in three biological replicates

Additional file 5: Table S1. Records of the birthdates and litter sizes of the mice

Additional file 6: Table S2. Records of the water and feed intake of the mice

Additional file 7: Table S3. Reproductive fertility performance of the mice

Additional file 8: Figure S5. Expression of Frizzled4, LPR6, and β-Catenin in the ovaries of adult female mice. Western blot analysis of relative Frizzled4, LPR6, and β-Catenin protein levels of mouse ovaries. The gray values of Frizzled4, LPR6, and β-Catenin acquired by ImageJ software were normalized to GAPDH. The values are expressed as means ± SD of three biological replicates

Additional file 9: Figure S6. Expression of the *Csnk1a1* gene and CK1α protein during early embryonic and gonadal development in mice.CK1α protein-coding genes *Csnk1a1 *were expressed during early gonadal development in mice. Heat mapping data are summarized in the published literature.Immunofluorescent double staining for the expression of DDX4and CK1αin control and cKO mice ovaries. DAPIwas used to stain the nucleus. Scale bar = 100 µm.Western blot was used to detect the protein expression of CK1α in the ovaries of 16.5 dpc and 18.5 dpc female mice. Protein expression data were normalized to GAPDH and quantified by using ImageJ software. The data are expressed means ± SD of three biological replicates.

Additional file 10. Figure S7. Histological analysis of ovarian GCs from the eight-week control and cKO mice. Paraffin slices of ovaries tissues were stained with hematoxylin, the binding of GCs in cKO mice was loose and irregular, the boundary of the zona pellucida between granulosa cells and oocytes disappeared, and oocytes were shrinking and formed atretic follicles. Scale bar = 100 μm. Each tissue was analyzed in three biological replicates

Additional file 11. Figure S8. Primary ovarian GCs activity and apoptosis in cKO mice.CCK-F assay was used to detect cell activity: calcein, PI, and DAPI. Scale bar = 25 μm.Results of flow cytometry analysis.The statistical analysis results of the total percentage of early and late apoptotic cells. Each experiment was performed in three biological replicates

Additional file 12. Figure S9. The inhibitor D4476 induces apoptosis in primary GCs from wild-type female ovaries.IF double staining for the expression of CK1αand FSHRin primary GCs from wild-type mice ovaries. DAPIwas used to stain the nucleus. Scale bar = 20 µm.Representative image of WB detecting the inhibitor efficiency of CK1α protein inside primary GCs. Relative protein levels were normalized to α-tubulin and quantified by using ImageJ software.E_2_ content in culture media measured by ELISA.The bright field image of primary GCs.Representative images of flow cytometry.The statistical analysis results of the total percentage of early and late apoptotic cells. Each experiment has three biological replicates.

Additional file 13. Table 1. Primers used for RT-PCR

Additional file 14. The raw data

Additional file 15. The individual data values for figures

## Data Availability

All data generated or analysed during this study are included in this published article, its supplementary information files and publicly available repositories. The raw data are provided in Additional file 14. The individual data values for figures are provided in Additional file 15. The RNA-seq data were submited to the SRA database (https://www.ncbi.nlm.nih.gov/sra/) under accession number PRJNA1050635.

## Abbreviations

AF: Atretic follicle
CK1α: Casein kinase 1α
cKO: Conditional knockout
CYP19A1: Cytochrome P450 subfamily 19 member 1
dpp: Days post-partum
DS: Discovery Studio
E_2_: Estradiol
E_1_: Estrone
E_3_: Estriol
EGFR: Epidermal growth factor receptor
ELISA: Enzyme-linked immunosorbent assay
ER: Estrogen receptors
ERE: Estrogen response element
FBS: Fetal bovine serum
FSH: Follicle-stimulating hormone
FSHR: FSH receptor
GAPDH: Glyceraldehyde-3-phosphate dehydrogenase
GCs: Granulosa cells
GO: Gene Ontology
hCG: Human chorionic gonadotropin
HRP: Horseradish peroxidase
IHC: Immunohistochemistry
KEGG: Kyoto encyclopedia of genes and genomes
LH: Luteinizing hormone
MAPK: Mitogen-activated protein kinase
PCA: Principal component analysis
PFA: Paraformaldehyde
PMSG: Pregnant mare serum gonadotropin
PVDF: Polyvinylidene fluoride
