# Supplementary figures and images for: Casein kinase 1α mediates estradiol secretion via CYP19A1 expression in mouse ovarian granulosa cells
Source: BMC Biol. 2024 Aug 26;22:176. doi: 10.1186/s12915-024-01957-3 (PMC11346181; doi:10.1186/s12915-024-01957-3)

**A**  
Ovary

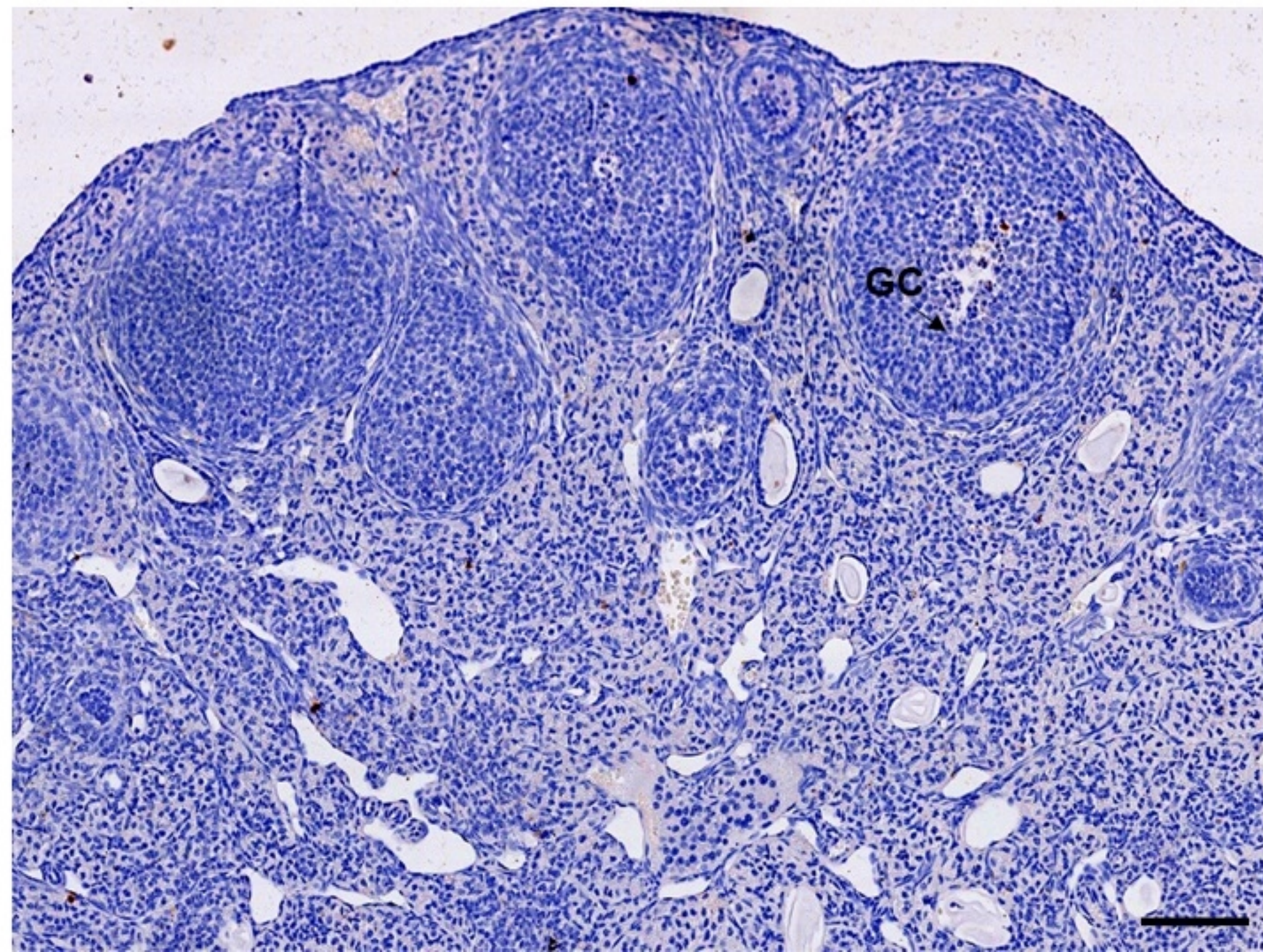

**B**  
Uterus

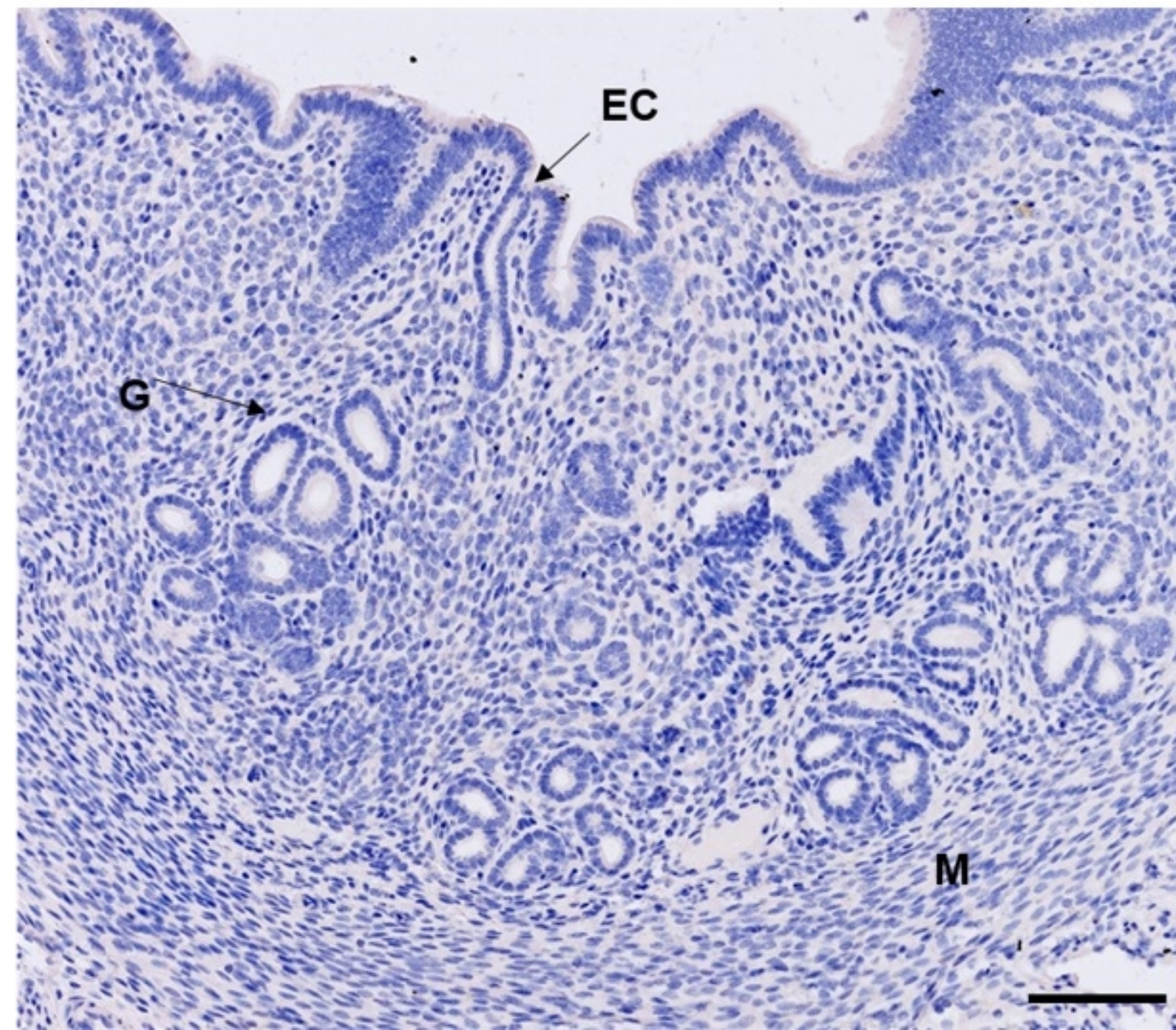

**C**  
Oviduct

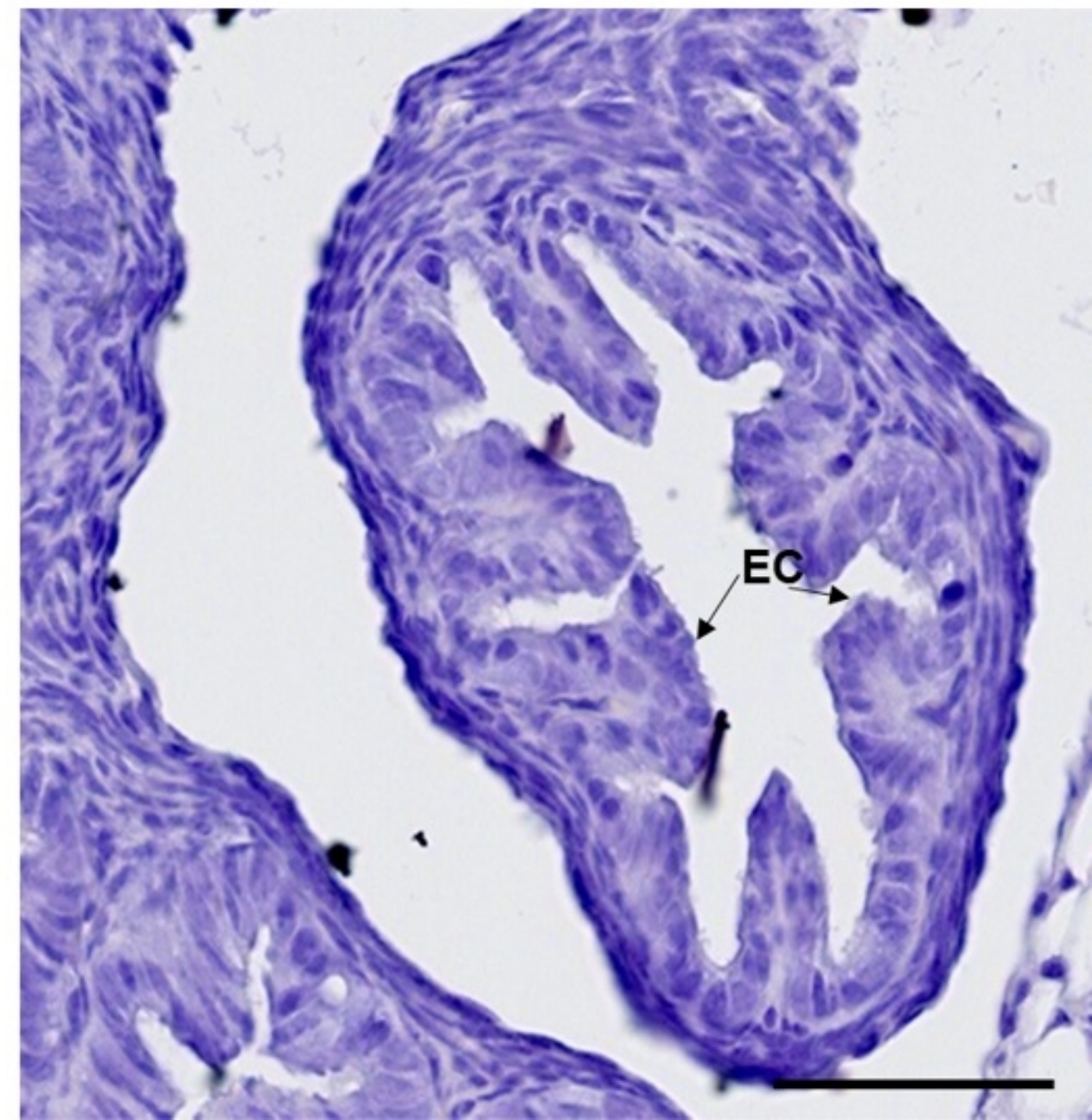

Supplement: Supplementary file 1 — Additional file 1: Figure S1. Negative control of CK1α in different tissues. The localization of CK1α in the ovaries, uterus and oviduct of an adult mouse detected by IHC. Paraffin slices of tissues were incubated with CK1α antibodies, and antibodies were replaced with anti-rabbit IgG for negative control. Granular cell, Epithelial cells, Glands, Myometrium. Each tissue was analyzed in three biological replicates. Scale bar = 100 μm [file 12915_2024_1957_MOESM1_ESM.pdf]

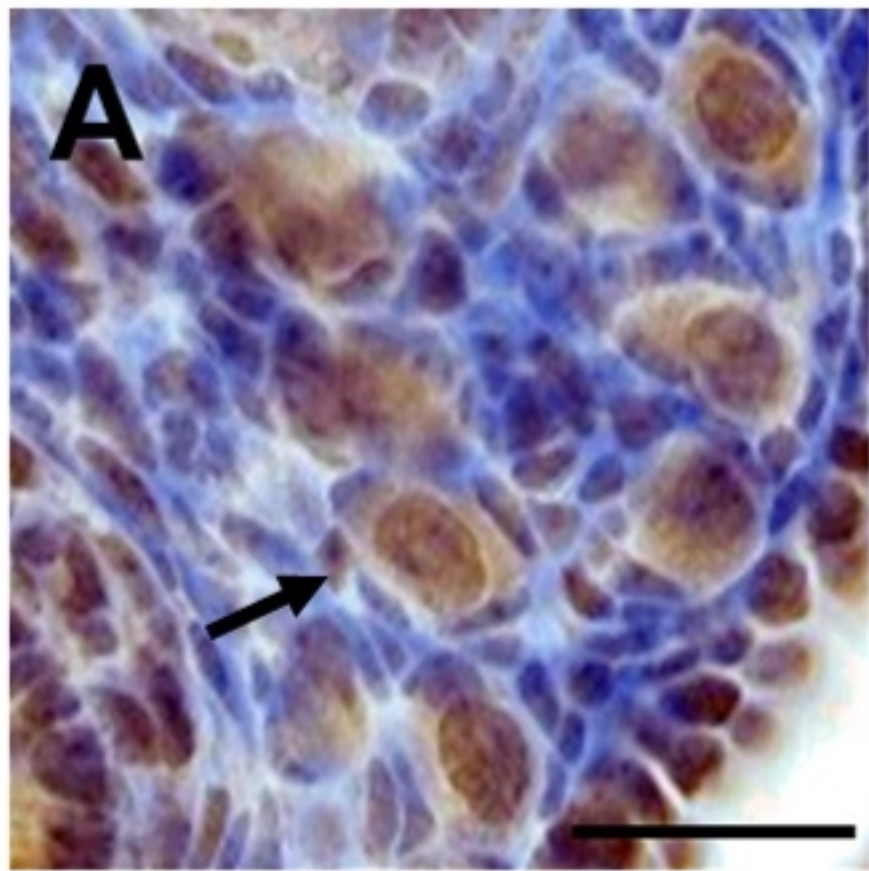

**primordial follicles**

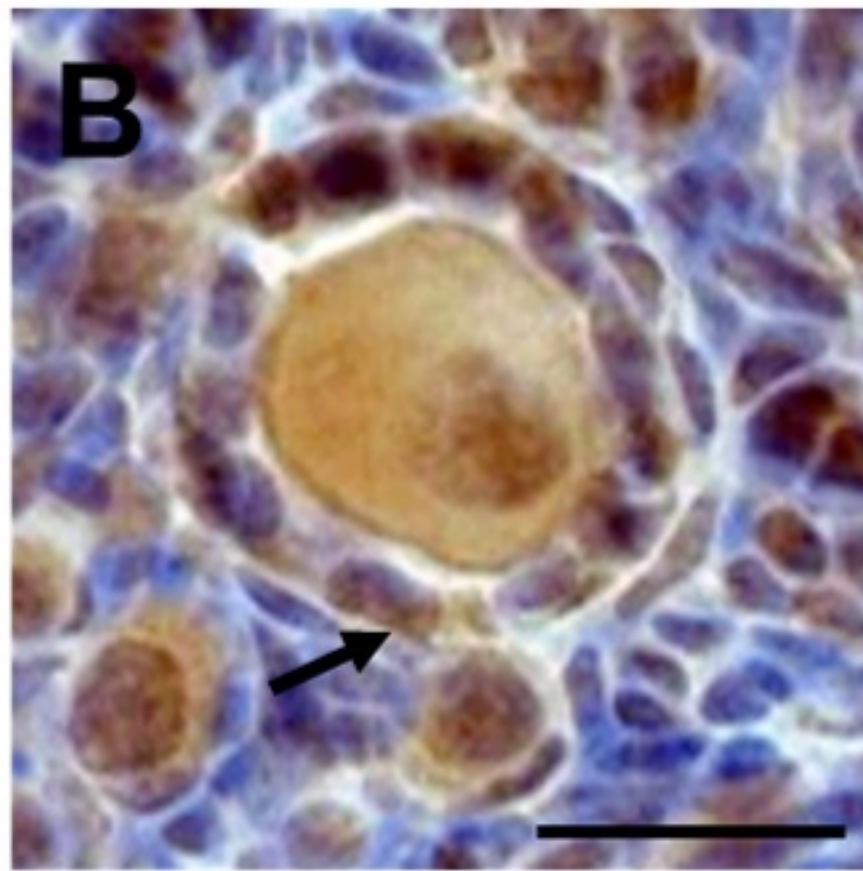

**primary follicle**

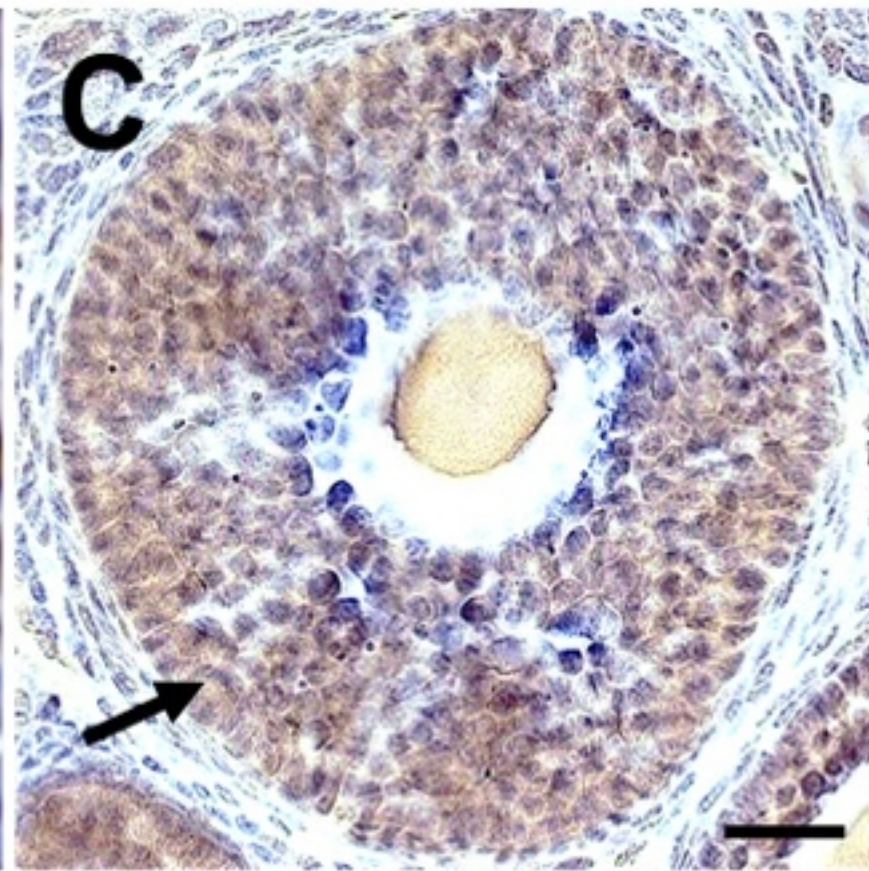

**secondary follicle**

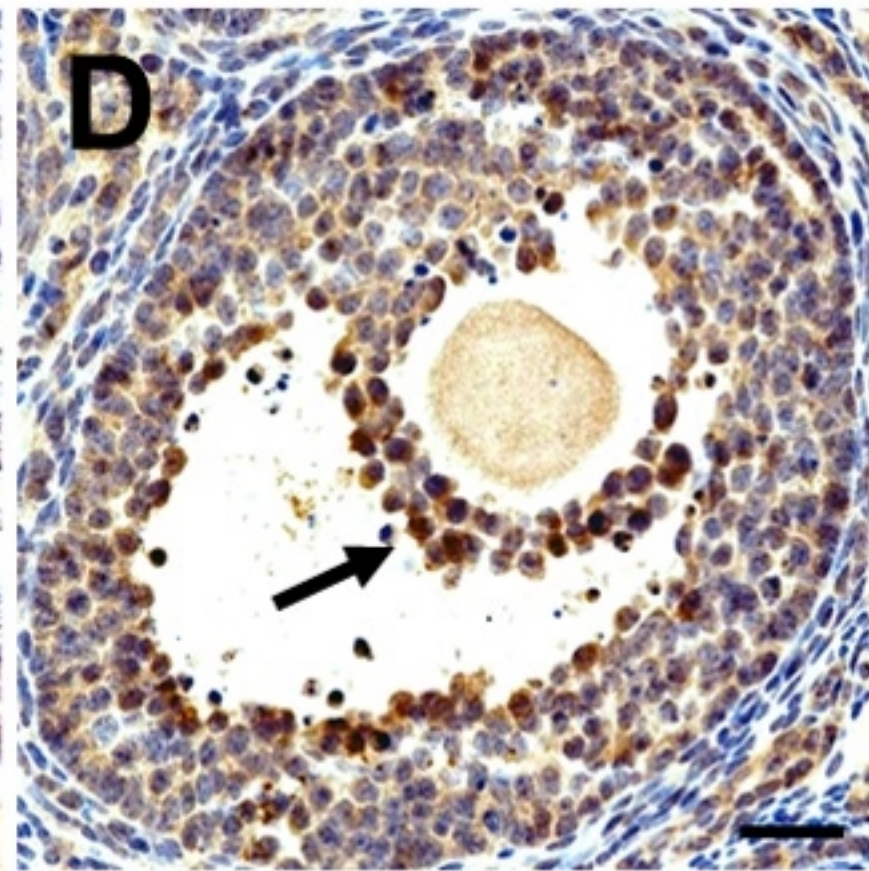

**antral follicle**

Supplement: Supplementary file 2 — Additional file 2: Figure S2. Expression of CK1α in follicles at different developmental stages. The localization of CK1α proteins of adult mouse ovary was performed by IHC.Primordial follicle: consists of a single oocyte and a single layer of flattened granulosa cells.Primary follicle, the granulosa cells proliferate from a single layer to multiple layers, and a transparent zone appears between the granulosa cells and the oocyte.Secondary follicle with multiple layers of granulosa cells around the oocyte, and the follicular antrum appears in the follicle.Antral follicle, the volume of the follicle is the largest at this time. Selective brown staining revealed CK1α-positive signals. Scale bar = 30 μm [file 12915_2024_1957_MOESM2_ESM.pdf]

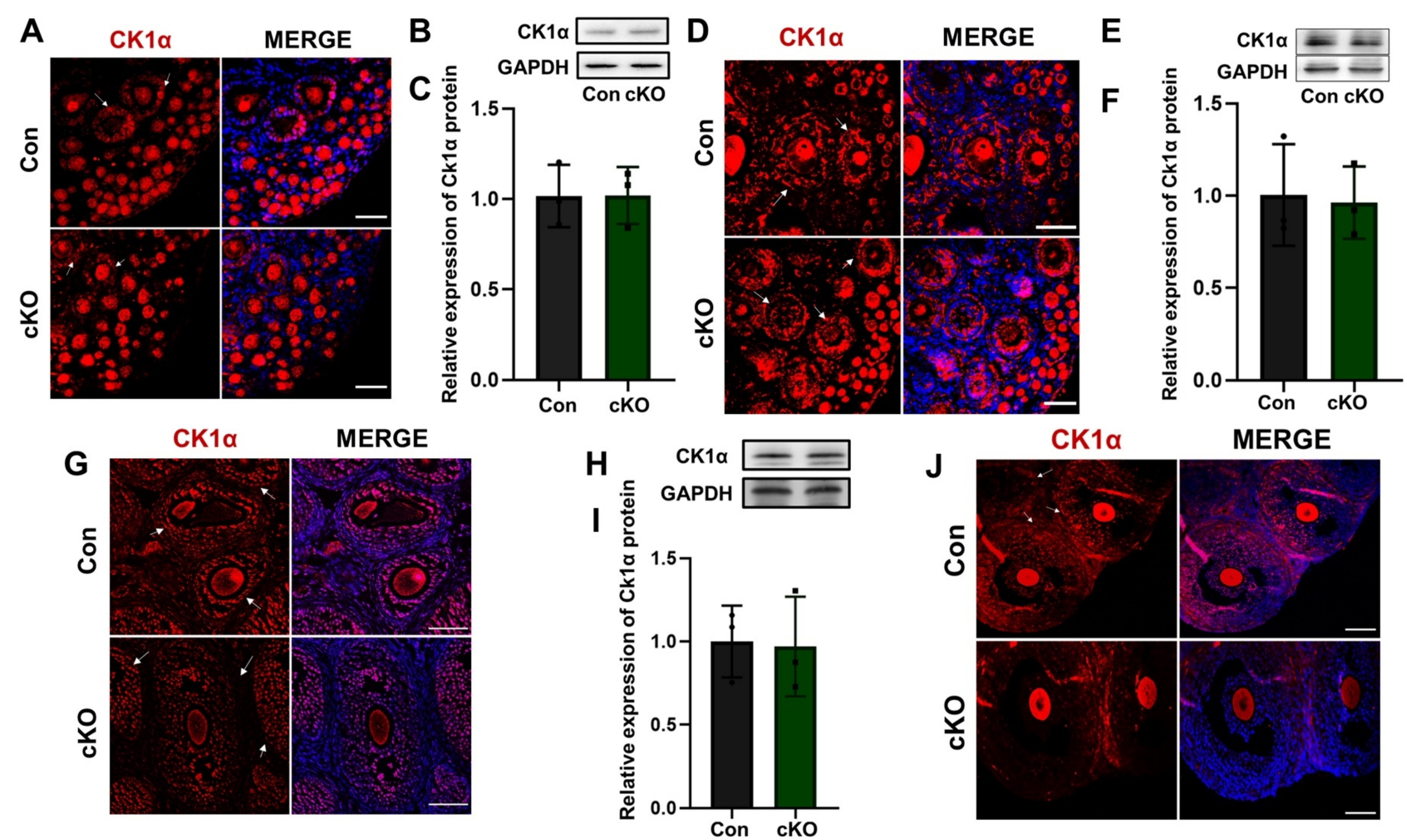

Supplement: Supplementary file 3 — Additional file 3: Figure S3. Deletion efficiency of cKO mice ovaries at different developmental stages. The expression of CK1α was investigated in ovarian tissue of cKO mice from postnatal three days, one week, four weeks, and eight weeks by immunofluorescence staining and WB method.Representative images of ovary sections IF staining. CK1α in red; DAPI stained nuclei in blue. Scale bar = 50 µm.Representative image of WB detecting the knockdown efficiency of CK1α protein inside ovarian GCs in vivo. Relative protein levels were analyzed by gray scanning and normalized to GAPDH. Each tissue was analyzed in three biological replicates [file 12915_2024_1957_MOESM3_ESM.pdf]

**A**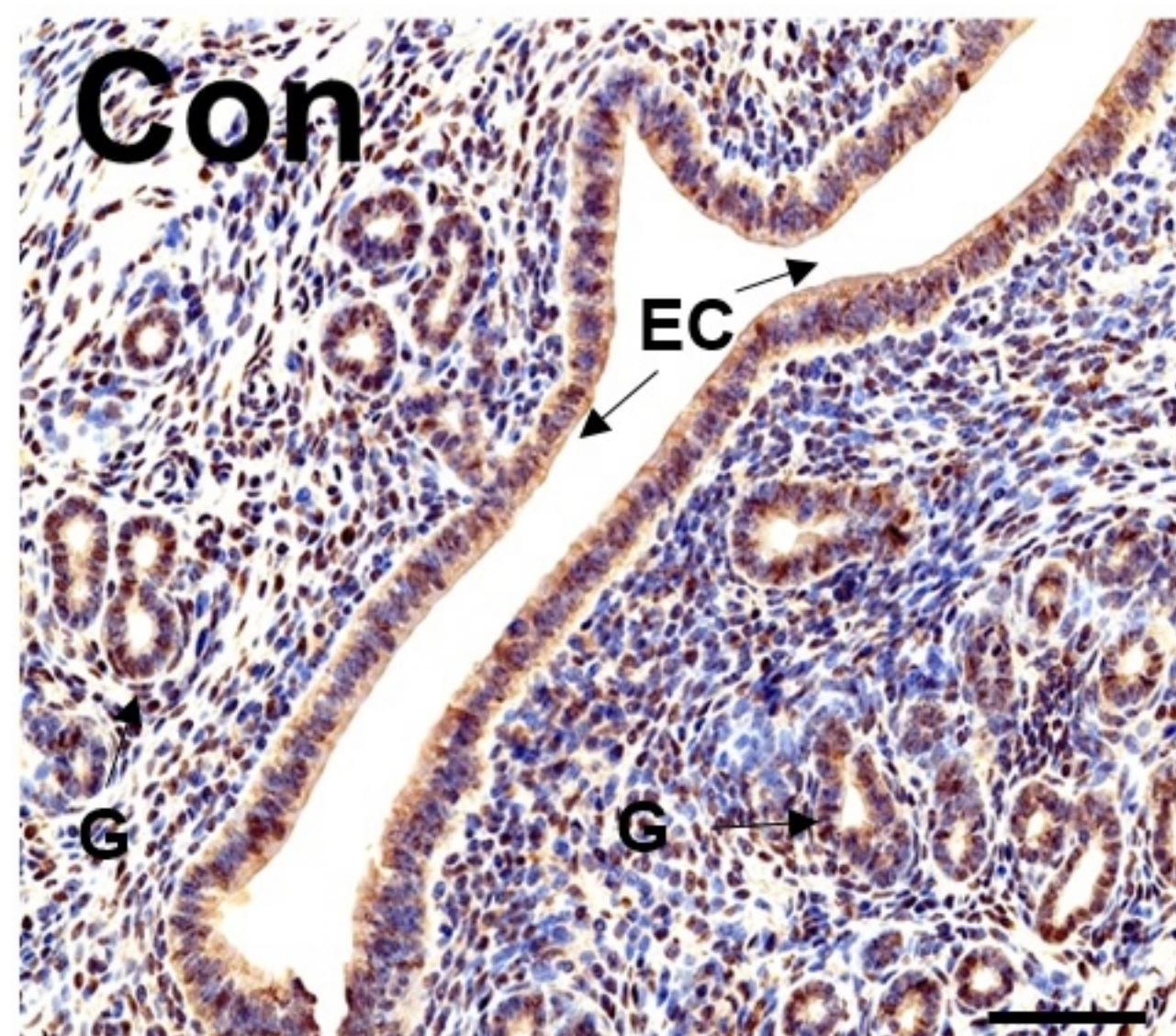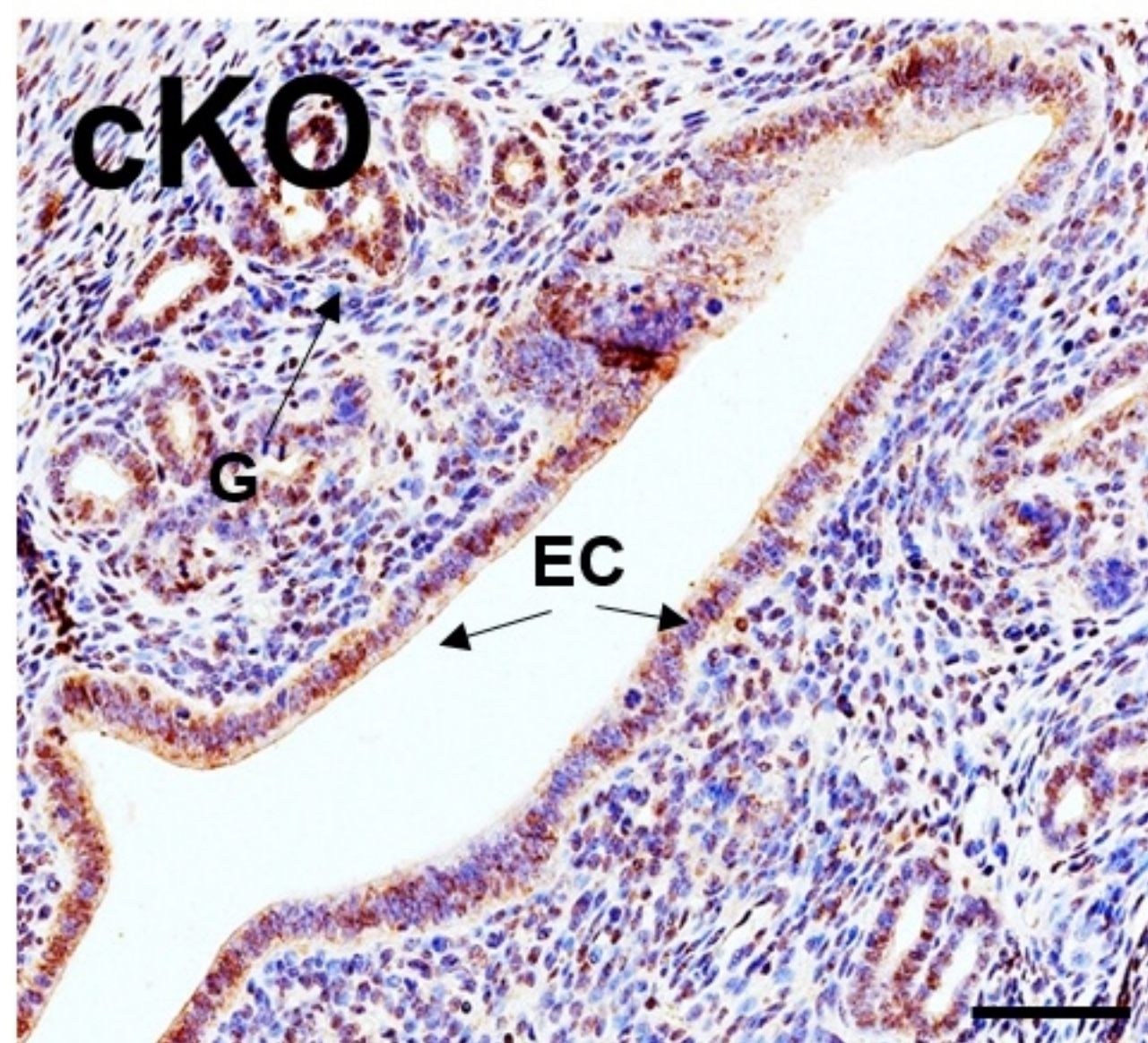**B****Con****cKO****Isthmus**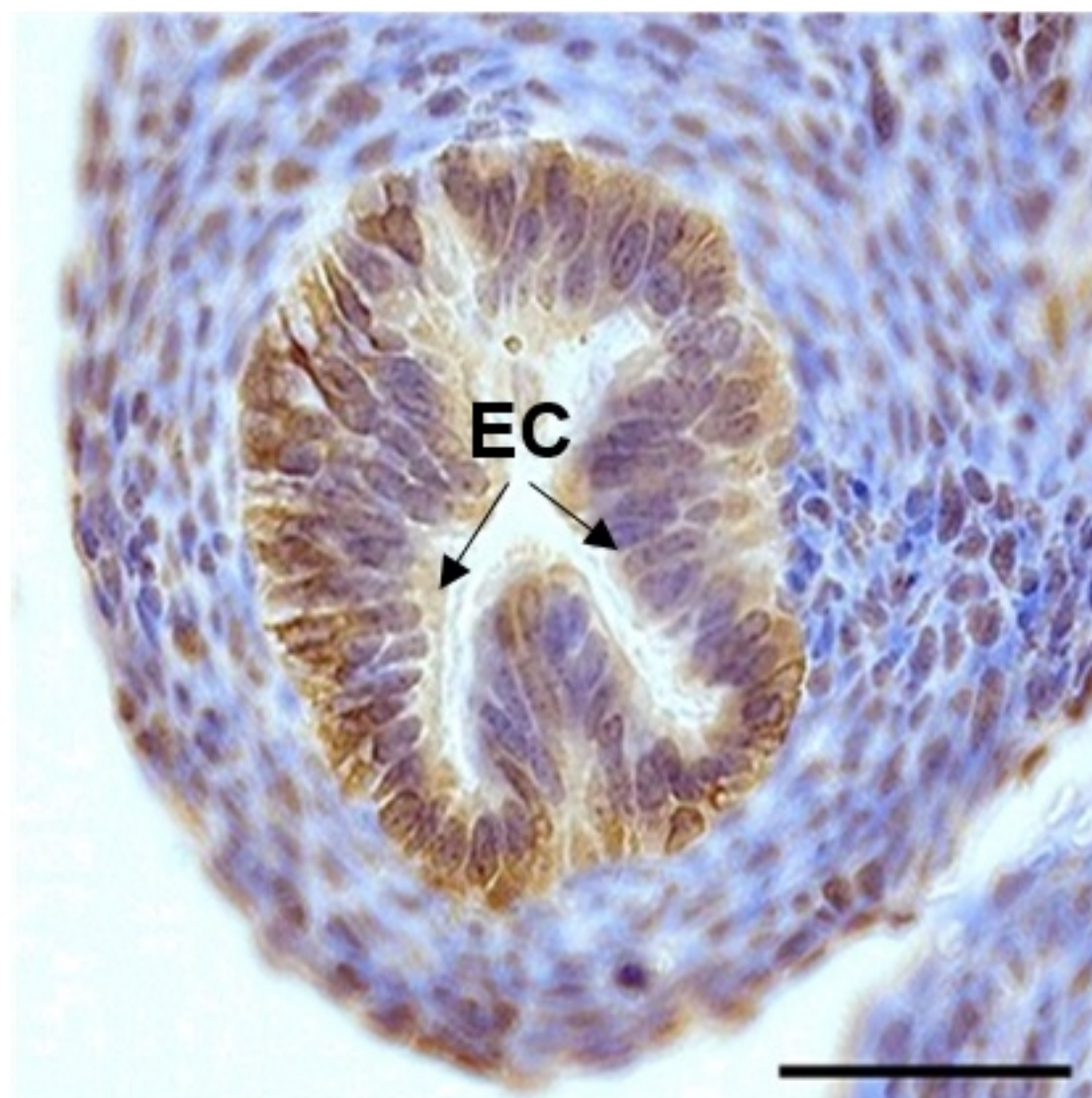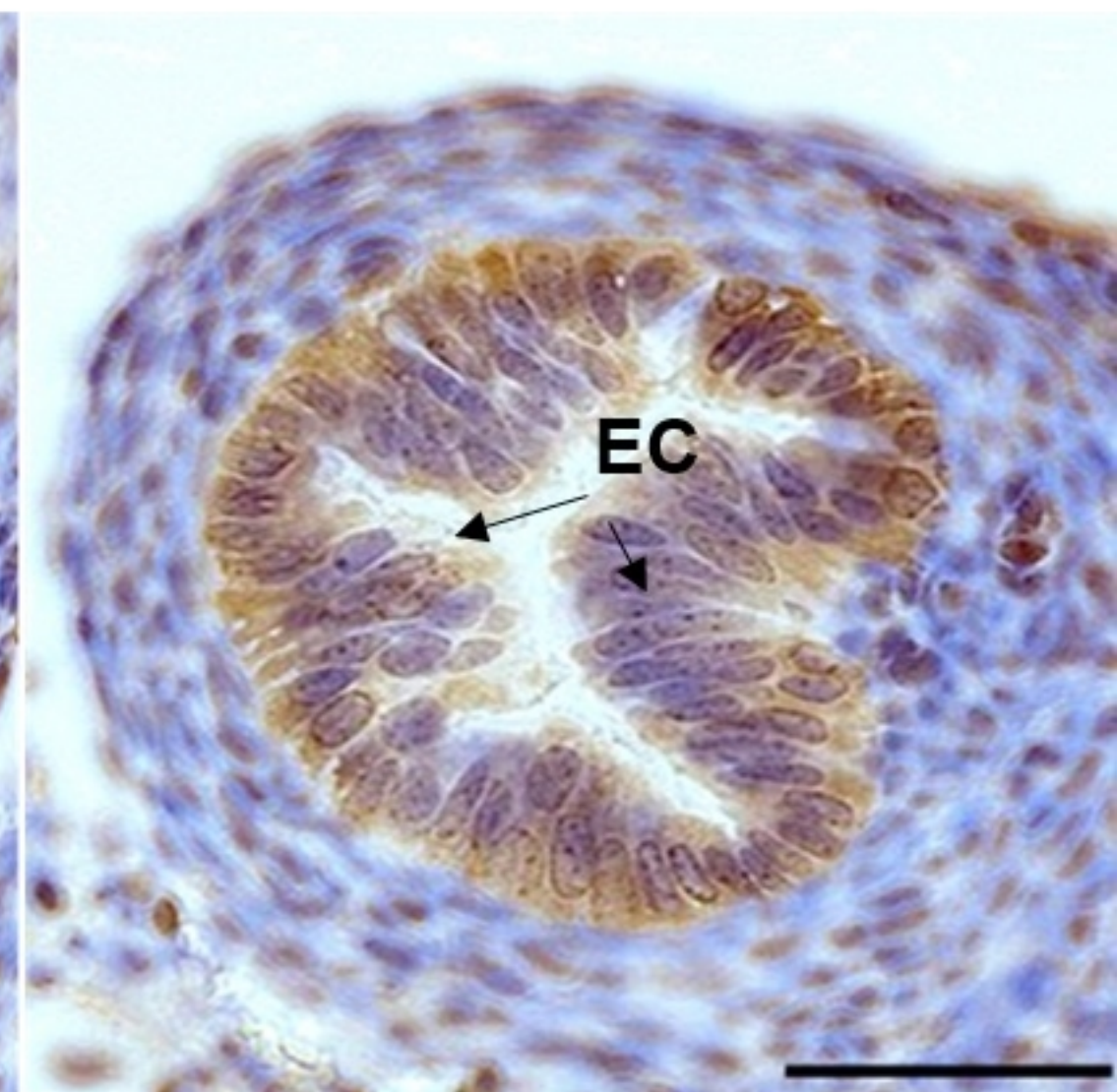**Ampulla**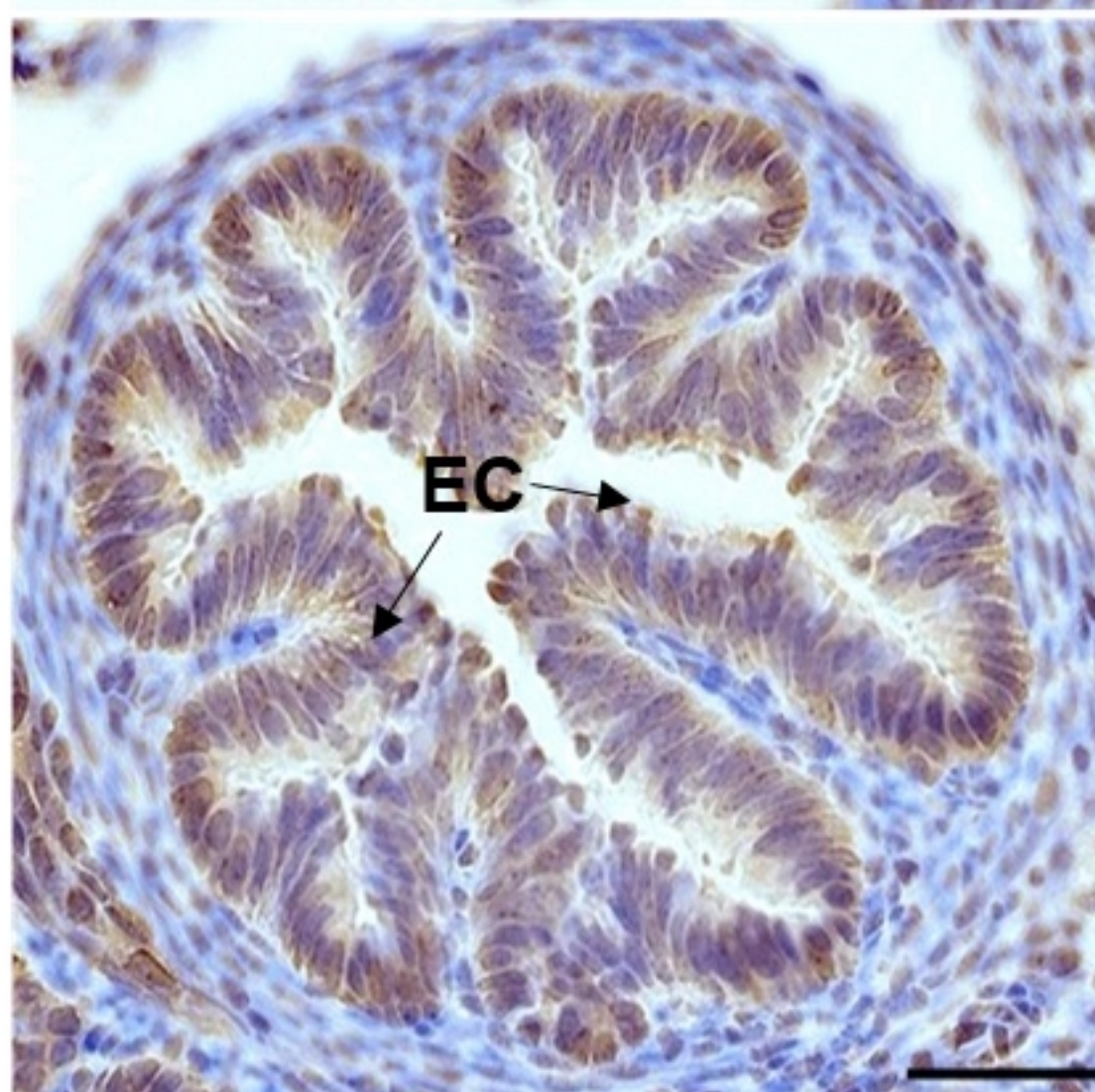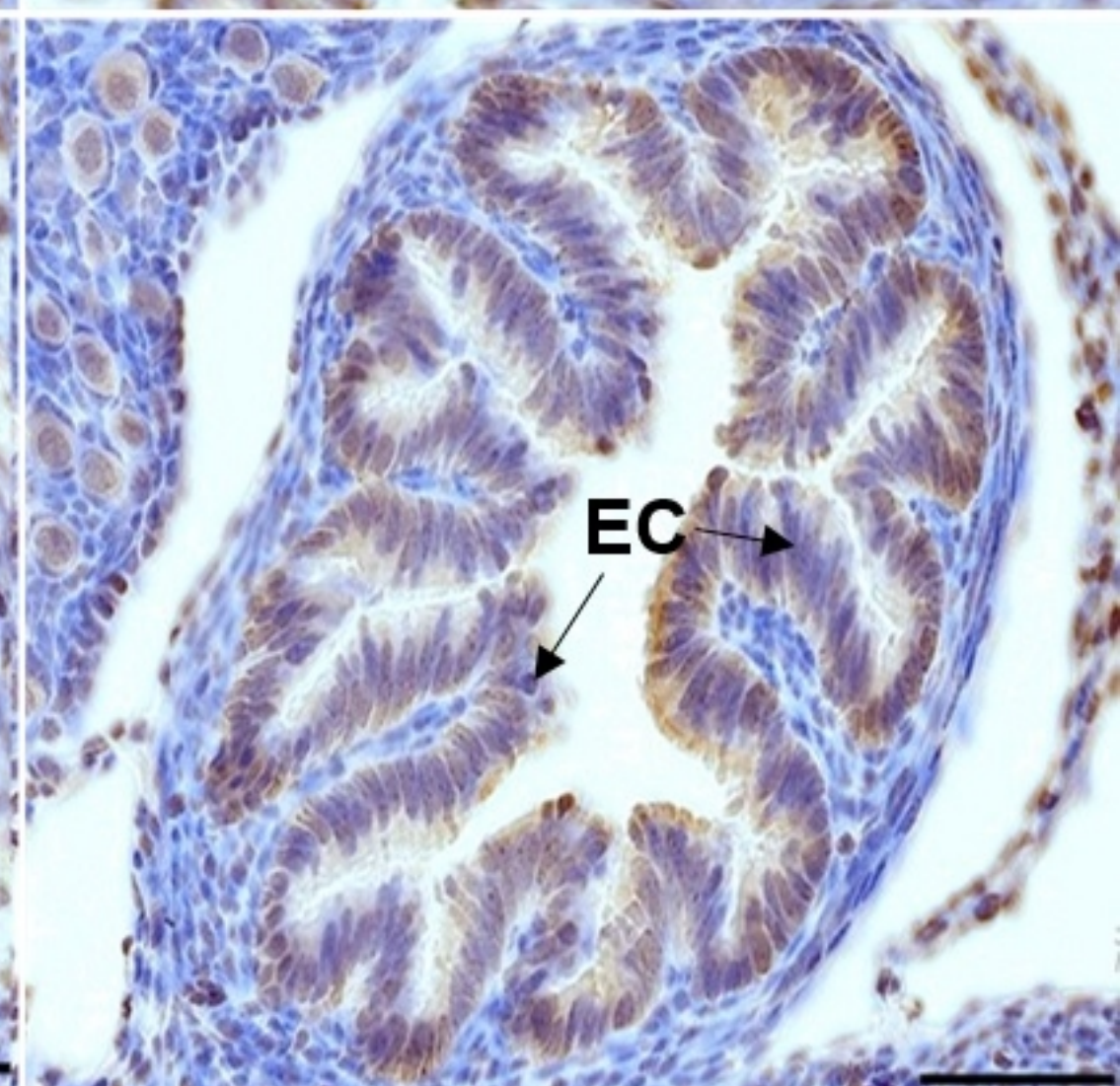

Supplement: Supplementary file 4 — Additional file 4: Figure S4. Expression of CK1α in the uterus and oviduct of adult female mice. The localization of CK1α in the uterusand oviductof an adult mouse detected by IHC. Paraffin slices of uterus and oviduct tissues were incubated with CK1α antibodies. Selectively staining brown demonstrated CK1α-positive signals. Scale bar = 50 μm. Epithelial cells, glands. Each tissue was analyzed in three biological replicates [file 12915_2024_1957_MOESM4_ESM.pdf]

**A**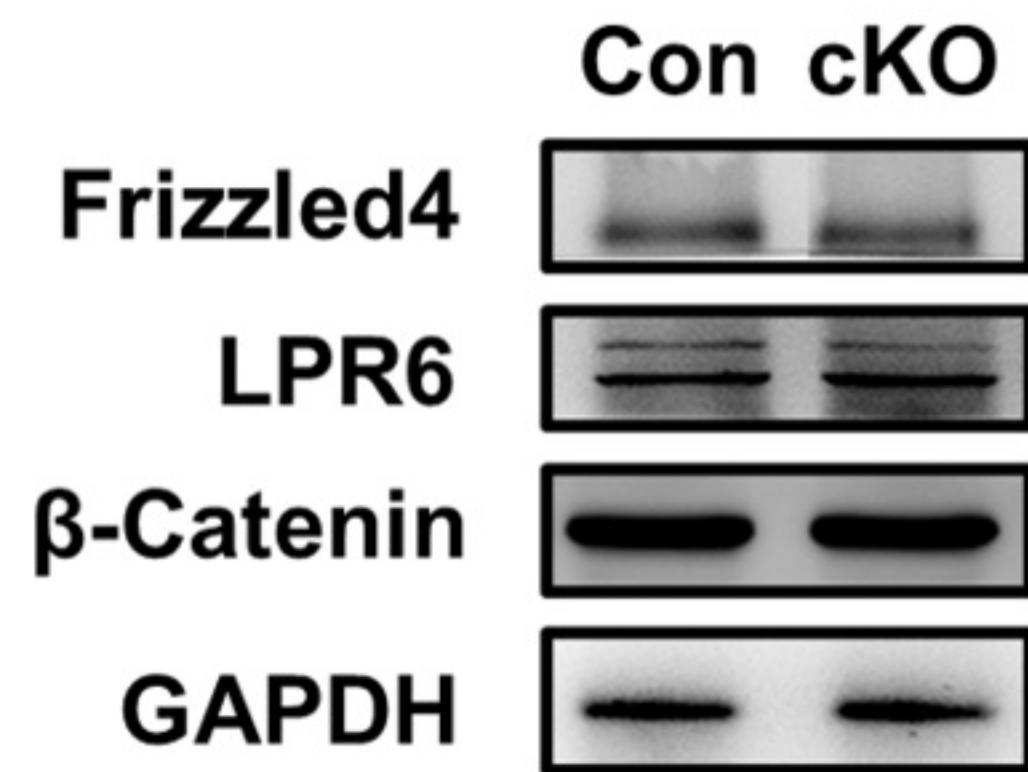**B**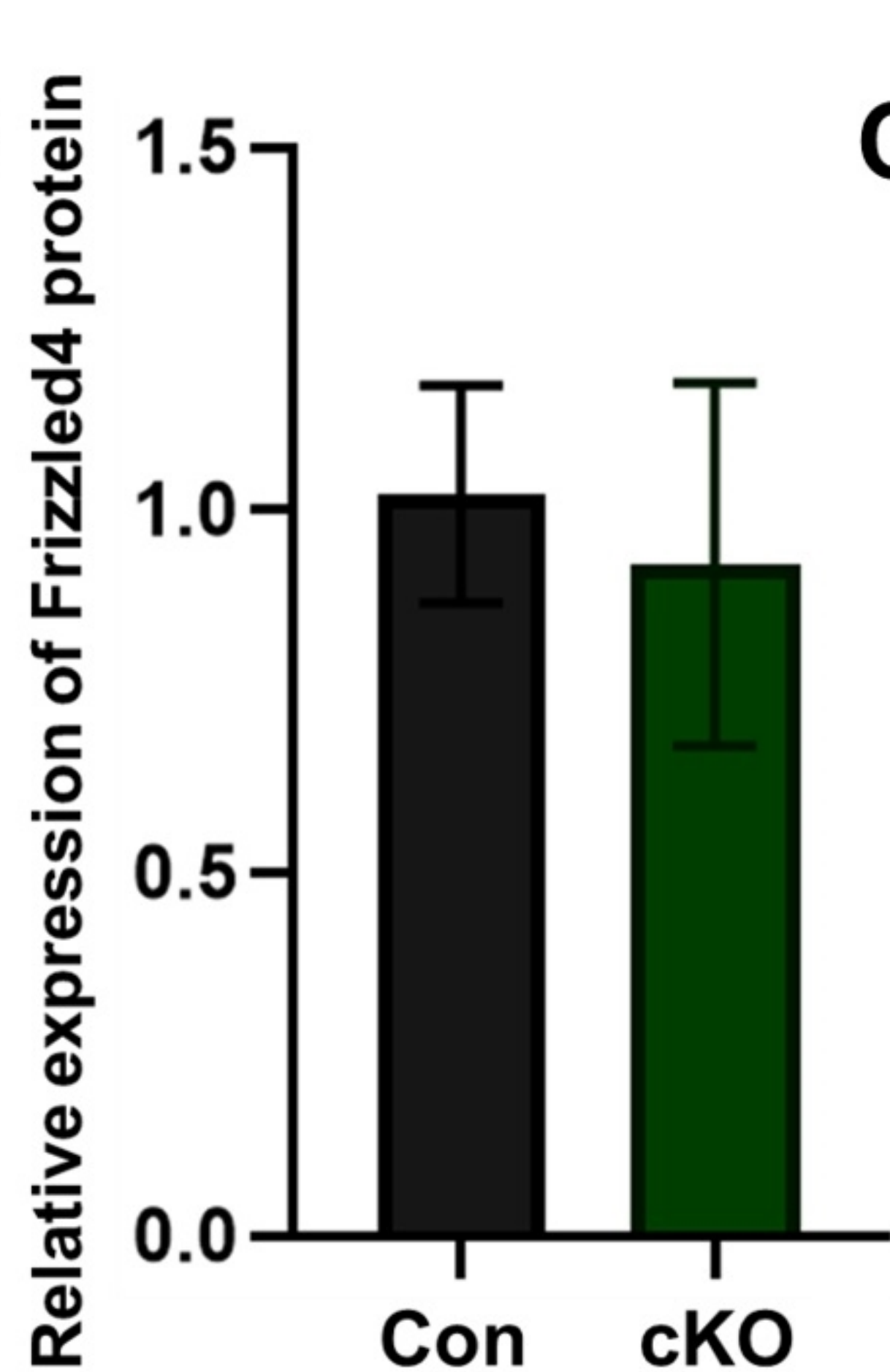**C**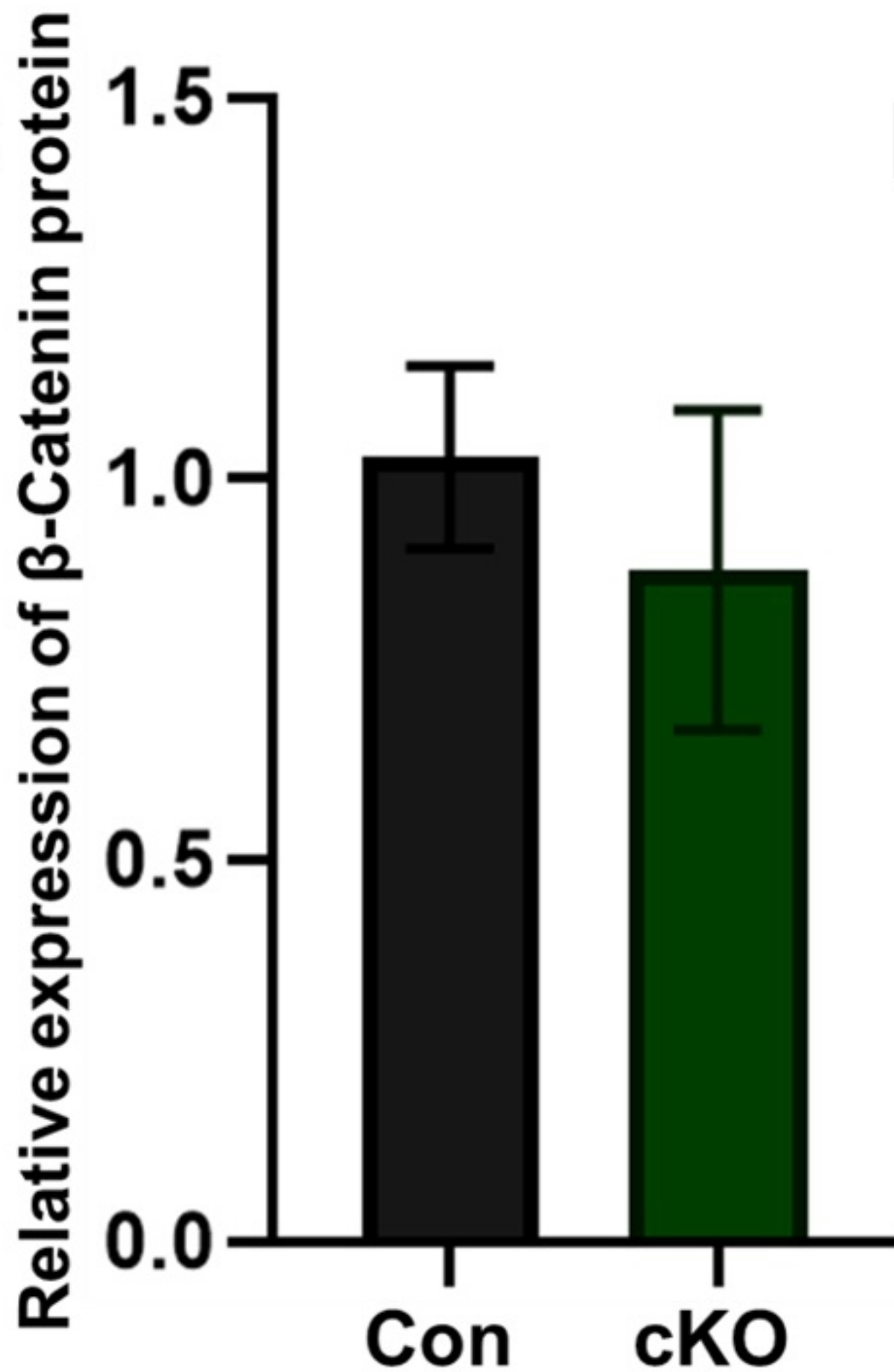**D**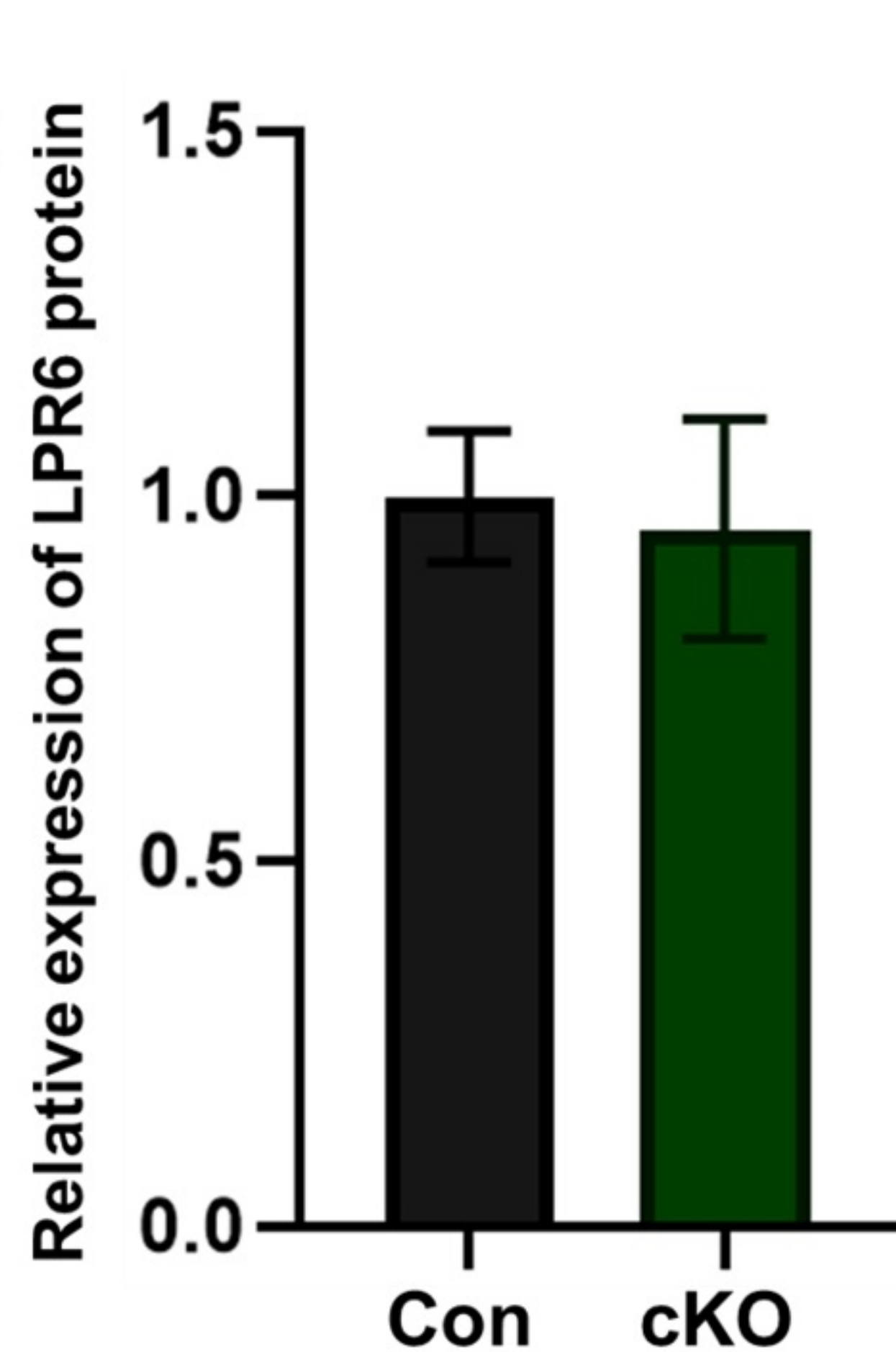

Supplement: Supplementary file 8 — Additional file 8: Figure S5. Expression of Frizzled4, LPR6, and β-Catenin in the ovaries of adult female mice. Western blot analysis of relative Frizzled4, LPR6, and β-Catenin protein levels of mouse ovaries. The gray values of Frizzled4, LPR6, and β-Catenin acquired by ImageJ software were normalized to GAPDH. The values are expressed as means ± SD of three biological replicates [file 12915_2024_1957_MOESM8_ESM.pdf]

**A**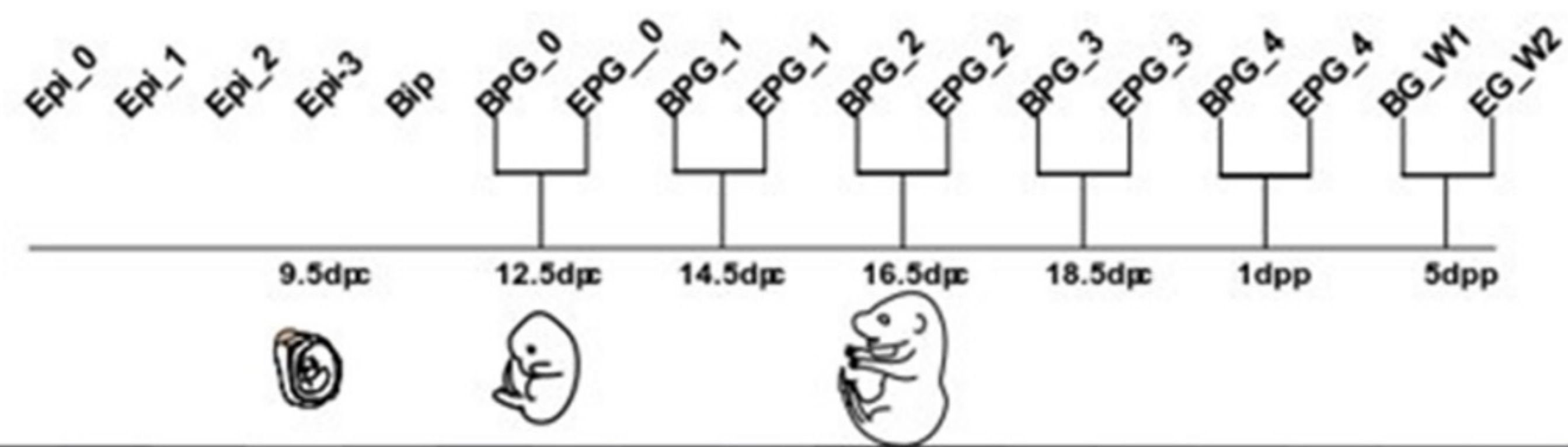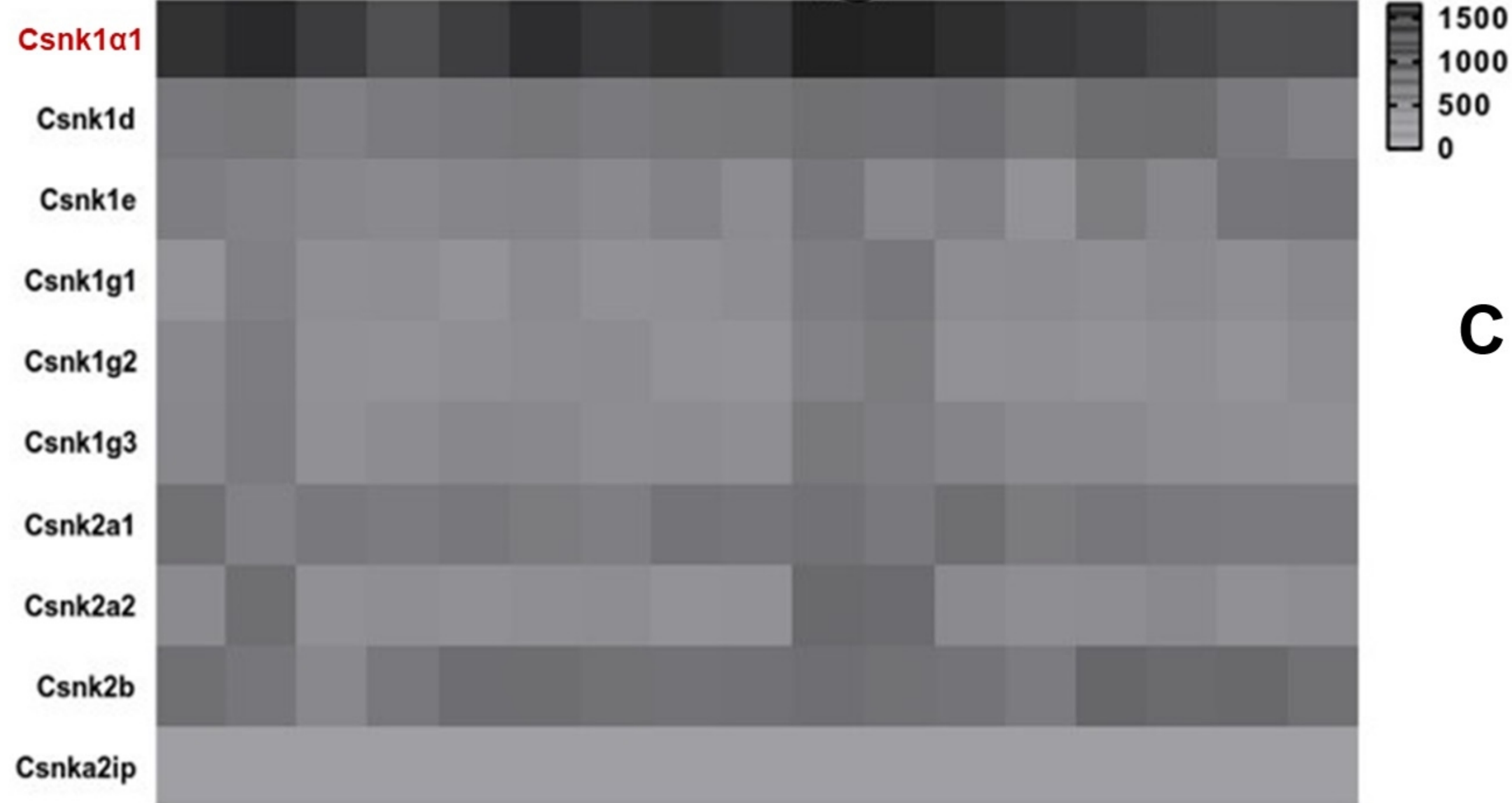**B**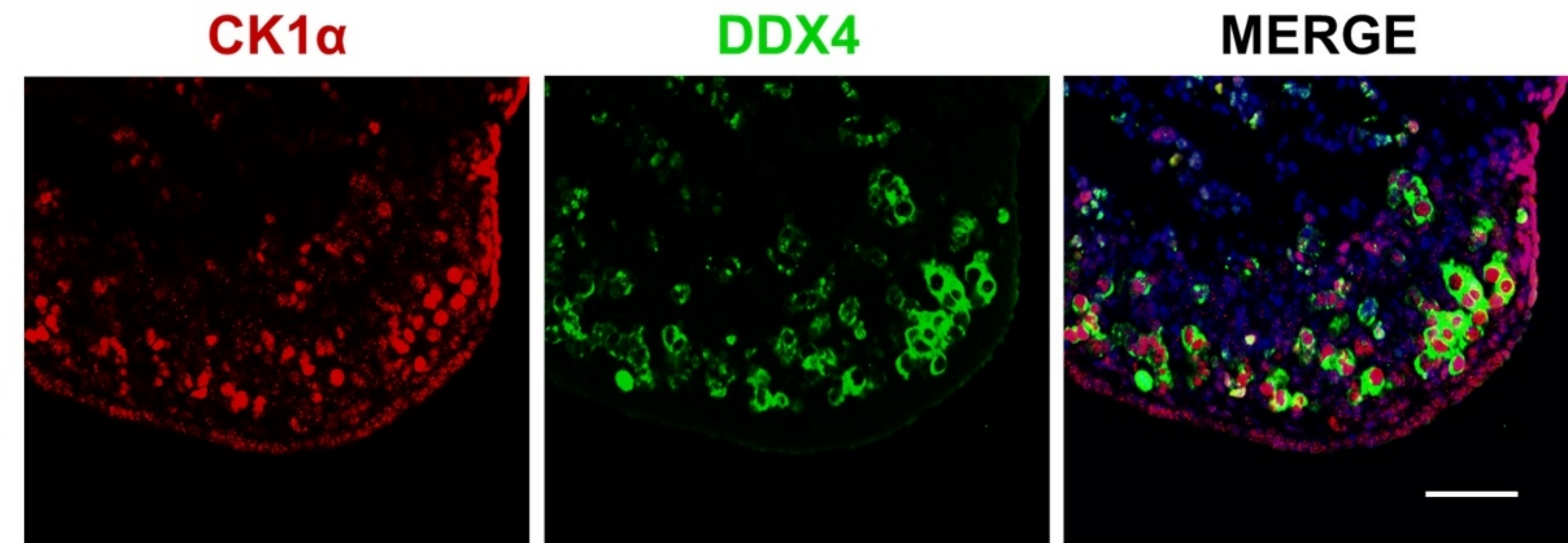**C**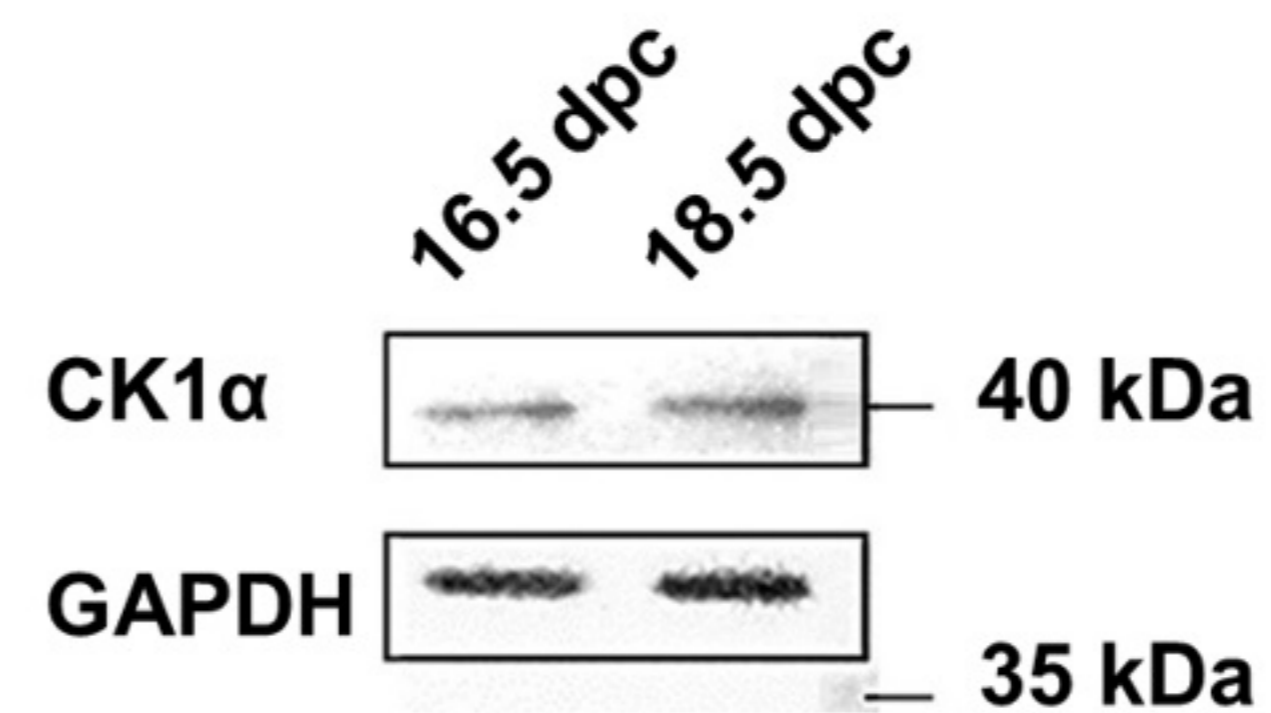**D**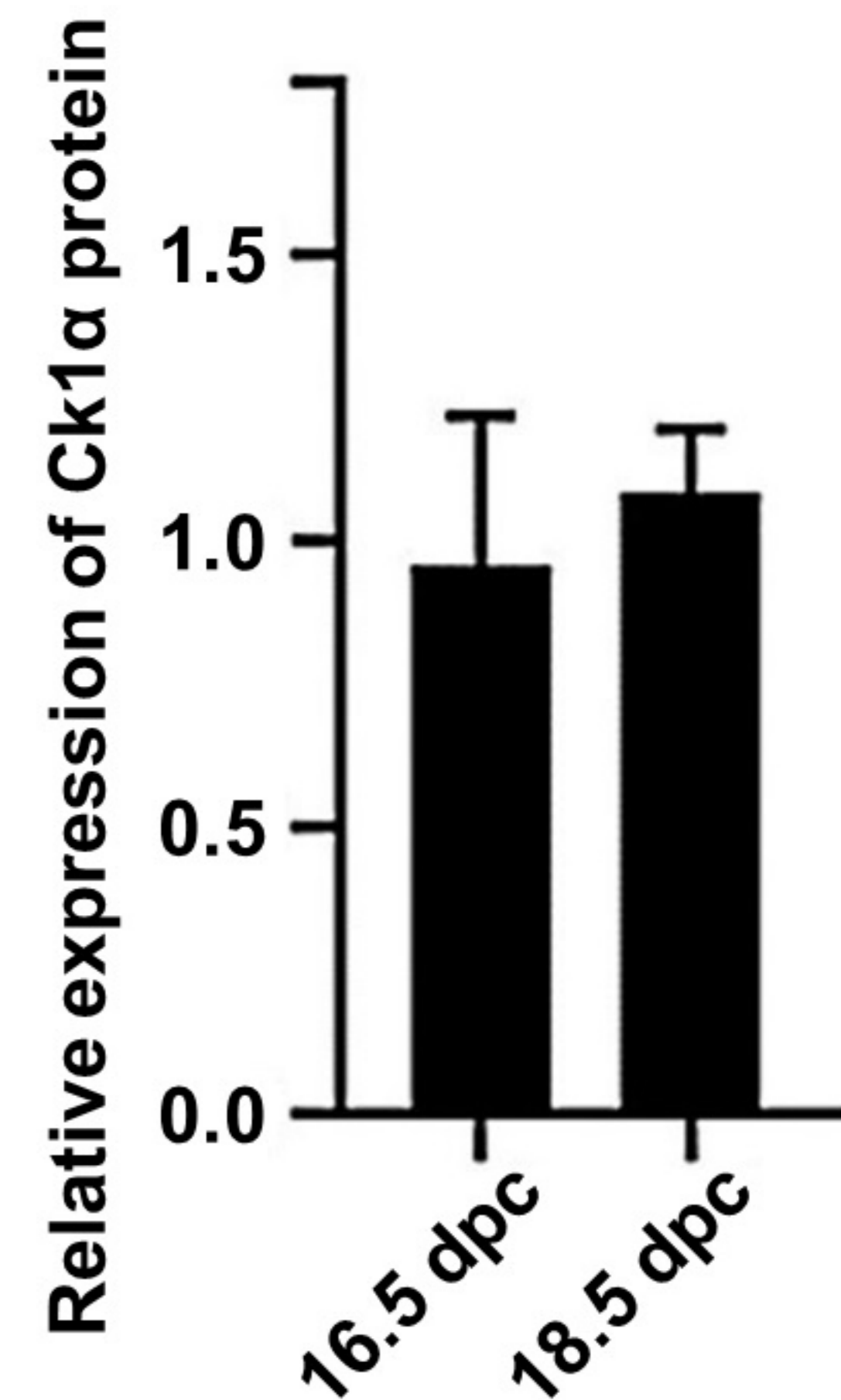

Supplement: Supplementary file 9 — Additional file 9: Figure S6. Expression of the Csnk1a1 gene and CK1α protein during early embryonic and gonadal development in mice.CK1α protein-coding genes Csnk1a1 were expressed during early gonadal development in mice. Heat mapping data are summarized in the published literature.Immunofluorescent double staining for the expression of DDX4and CK1αin control and cKO mice ovaries. DAPIwas used to stain the nucleus. Scale bar = 100 µm.Western blot was used to detect the protein expression of CK1α in the ovaries of 16.5 dpc and 18.5 dpc female mice. Protein expression data were normalized to GAPDH and quantified by using ImageJ software. The data are expressed means ± SD of three biological replicates. [file 12915_2024_1957_MOESM9_ESM.pdf]

**Con**

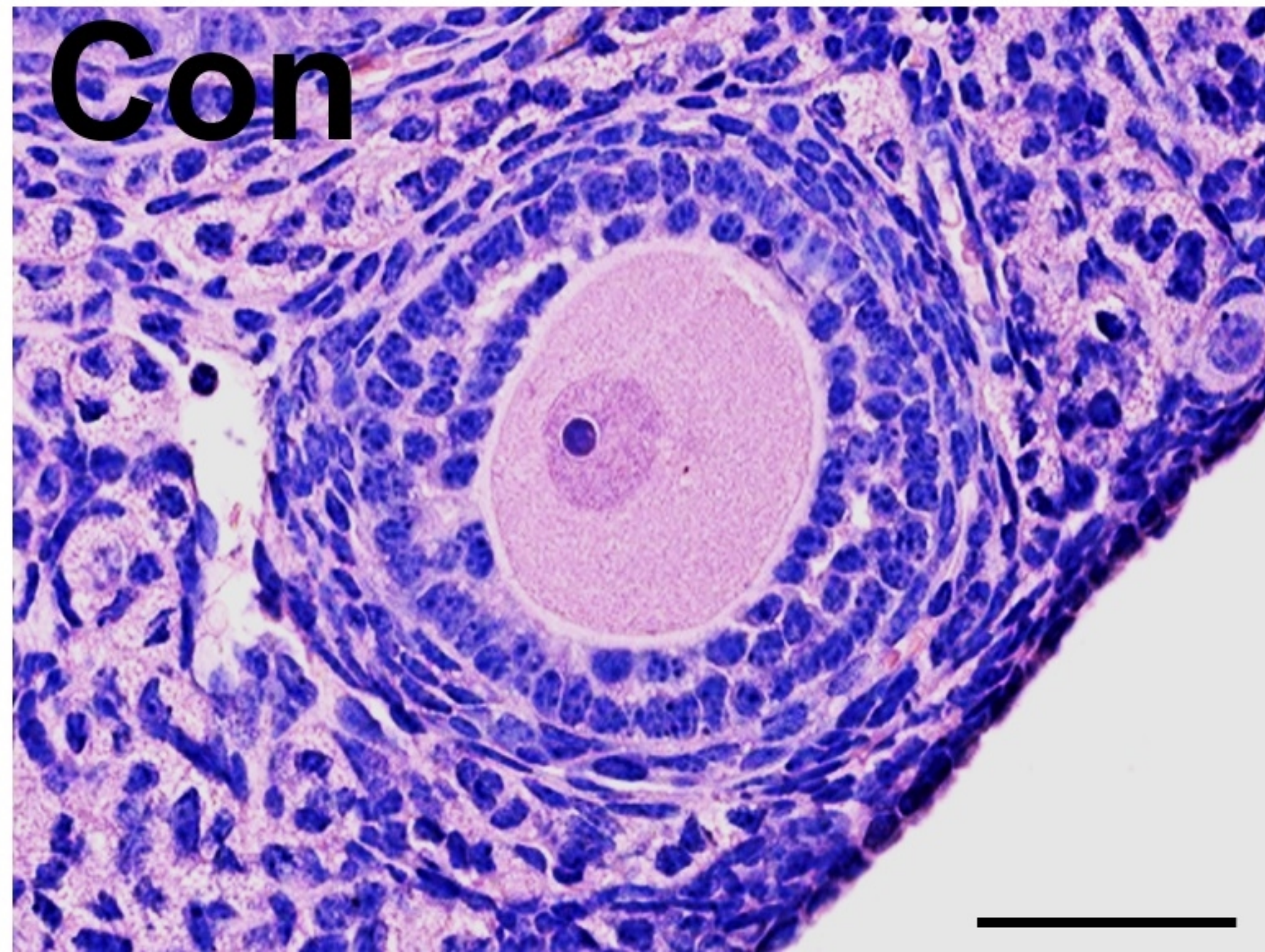

**ckO**

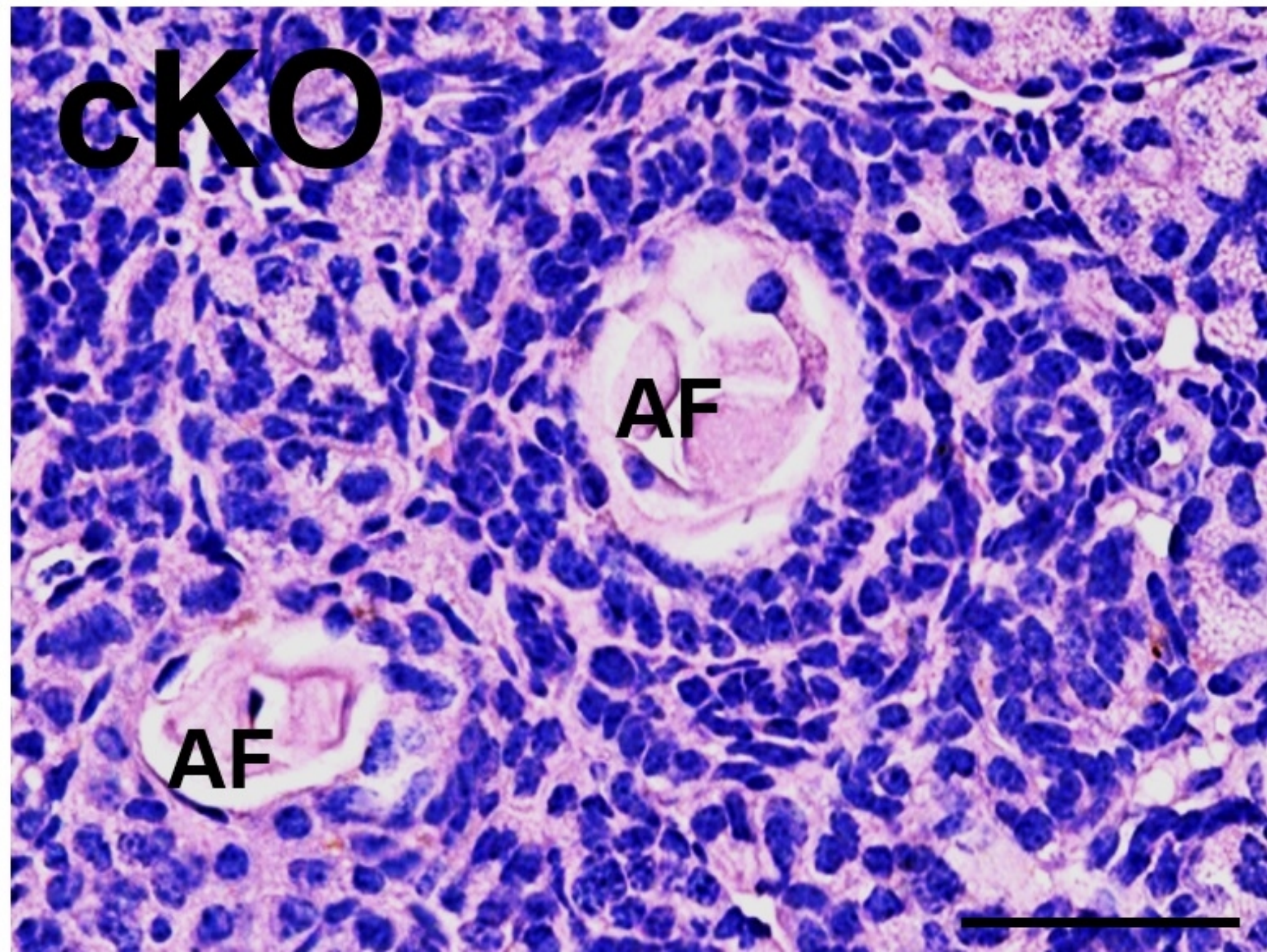

Supplement: Supplementary file 10 — Additional file 10. Figure S7. Histological analysis of ovarian GCs from the eight-week control and cKO mice. Paraffin slices of ovaries tissues were stained with hematoxylin, the binding of GCs in cKO mice was loose and irregular, the boundary of the zona pellucida between granulosa cells and oocytes disappeared, and oocytes were shrinking and formed atretic follicles. Scale bar = 100 μm. Each tissue was analyzed in three biological replicates [file 12915_2024_1957_MOESM10_ESM.pdf]

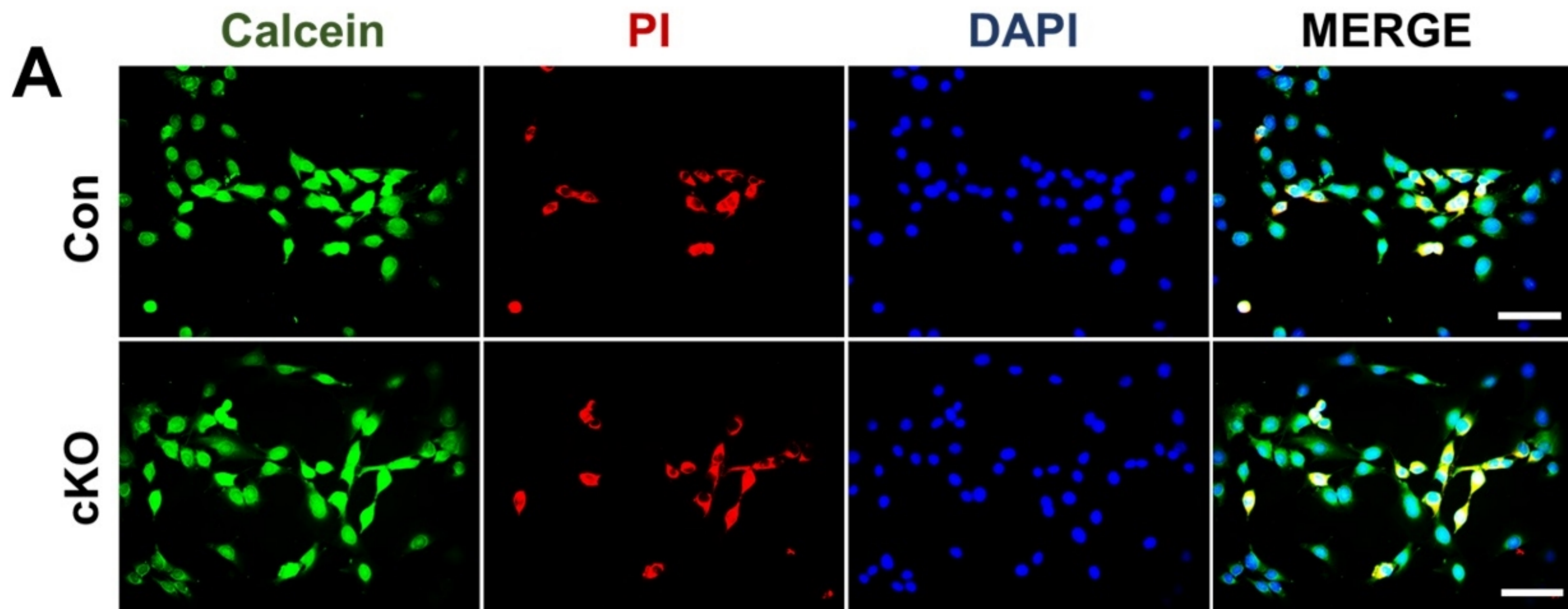

**B**

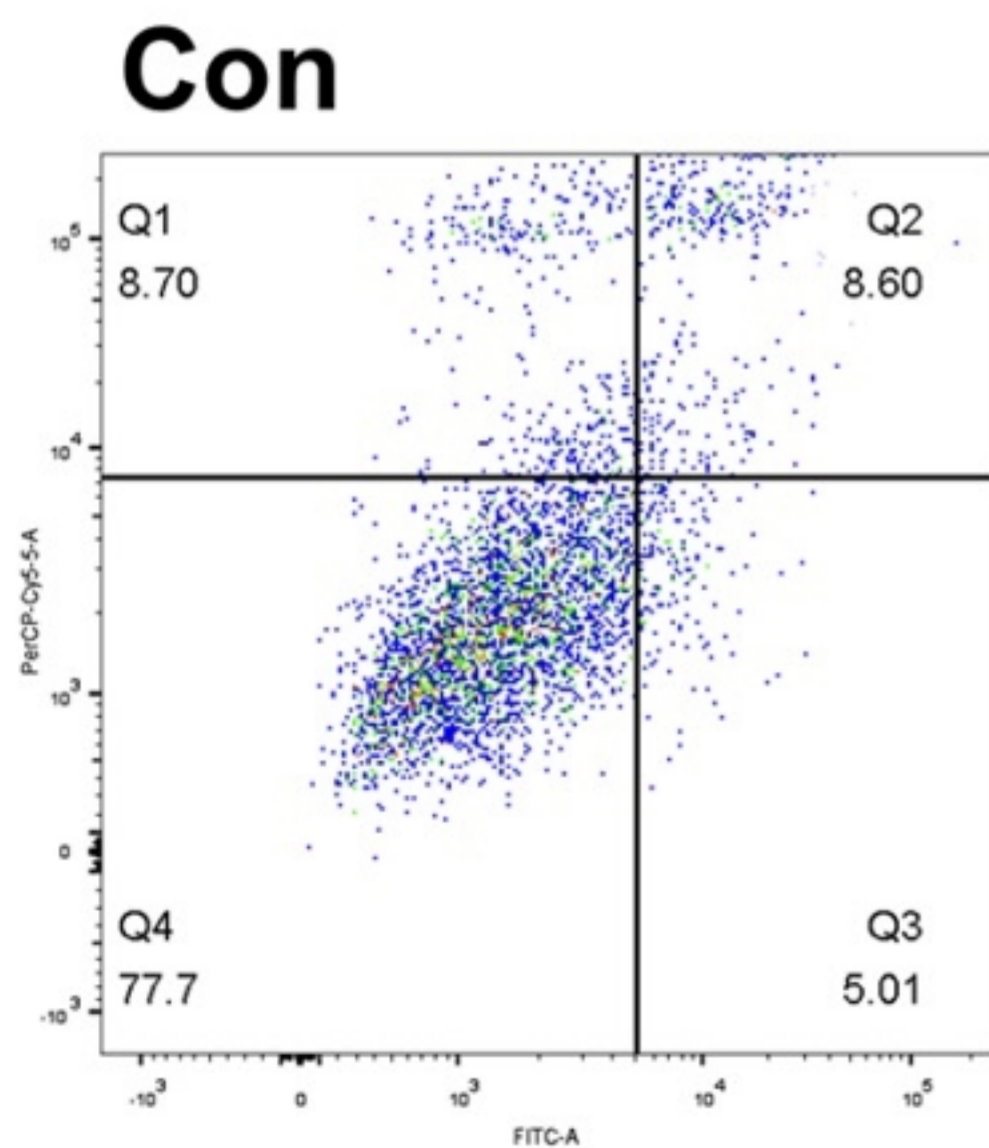

**cKO**

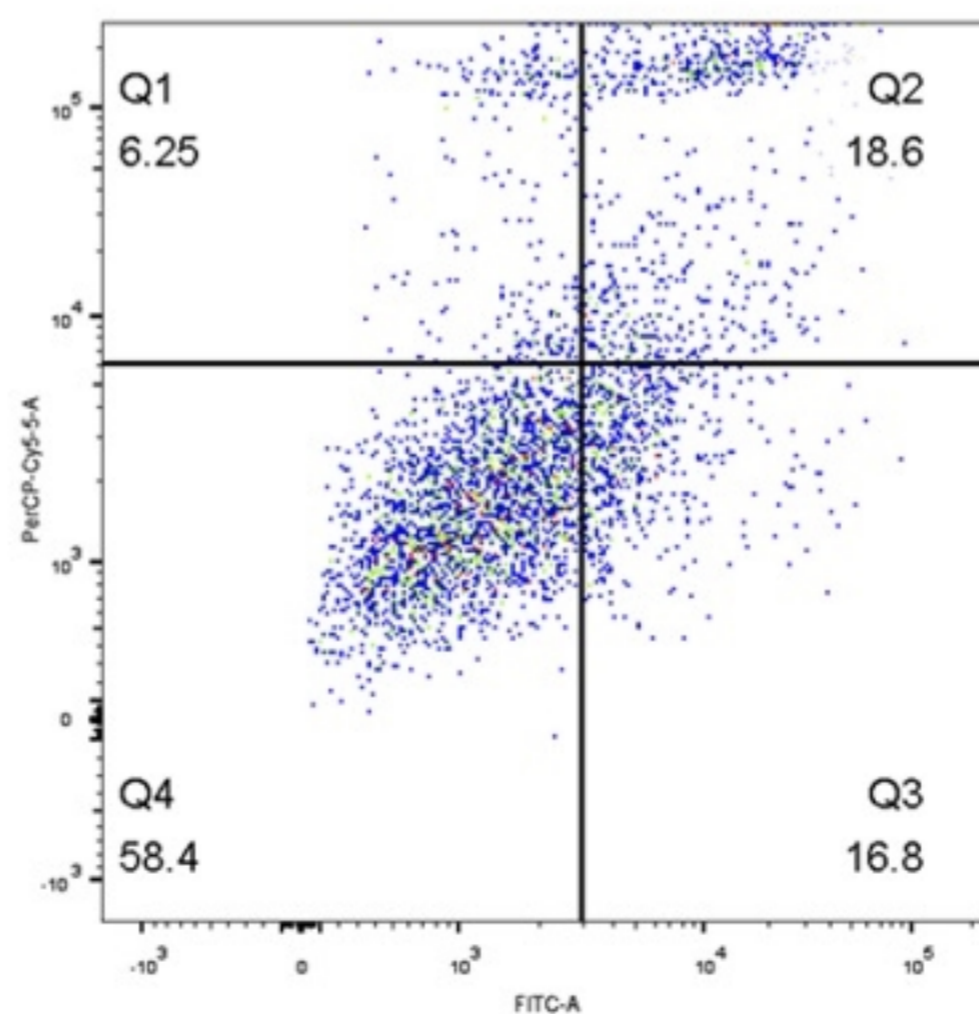

**C**

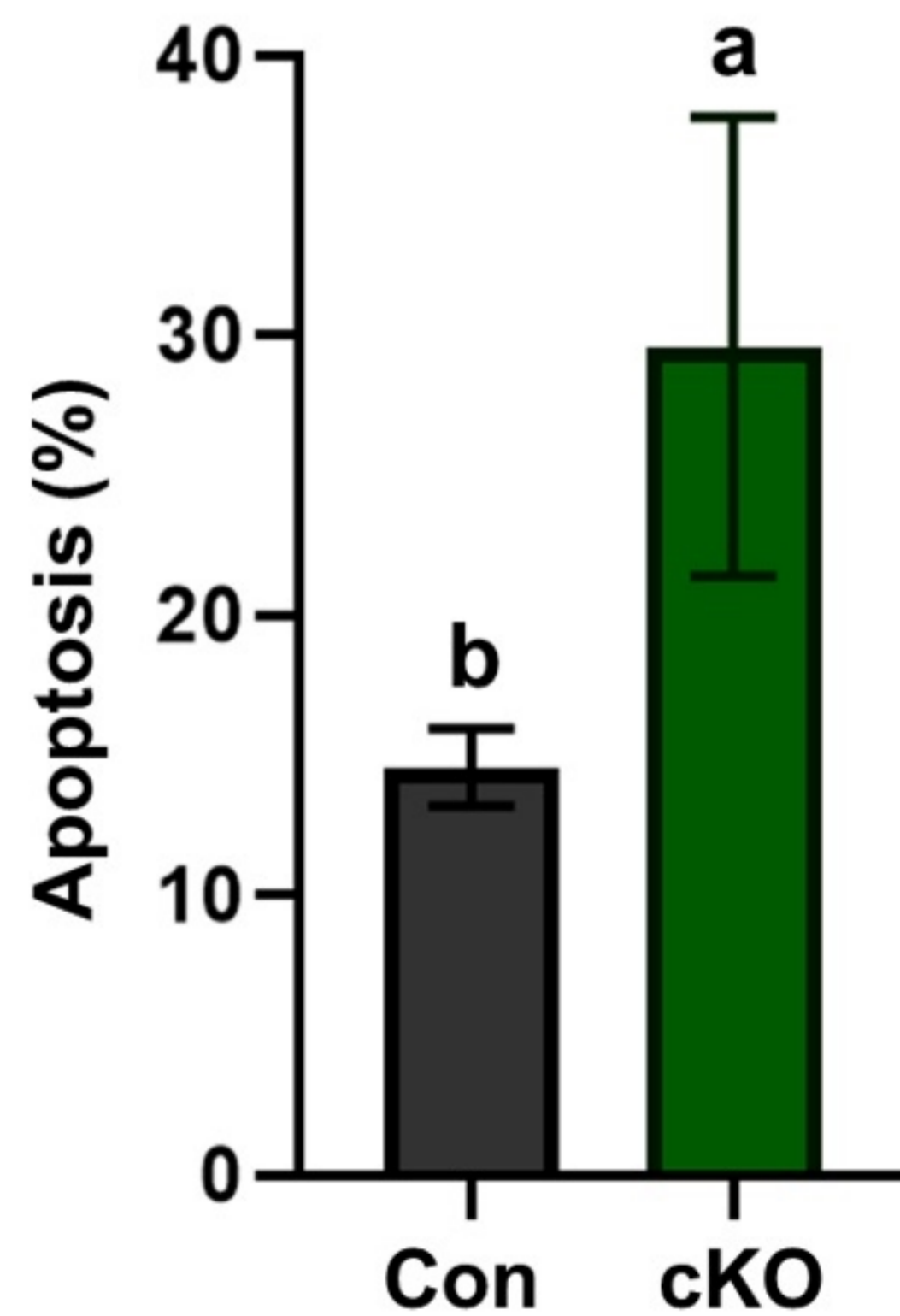

Supplement: Supplementary file 11 — Additional file 11. Figure S8. Primary ovarian GCs activity and apoptosis in cKO mice.CCK-F assay was used to detect cell activity: calcein, PI, and DAPI. Scale bar = 25 μm.Results of flow cytometry analysis.The statistical analysis results of the total percentage of early and late apoptotic cells. Each experiment was performed in three biological replicates [file 12915_2024_1957_MOESM11_ESM.pdf]

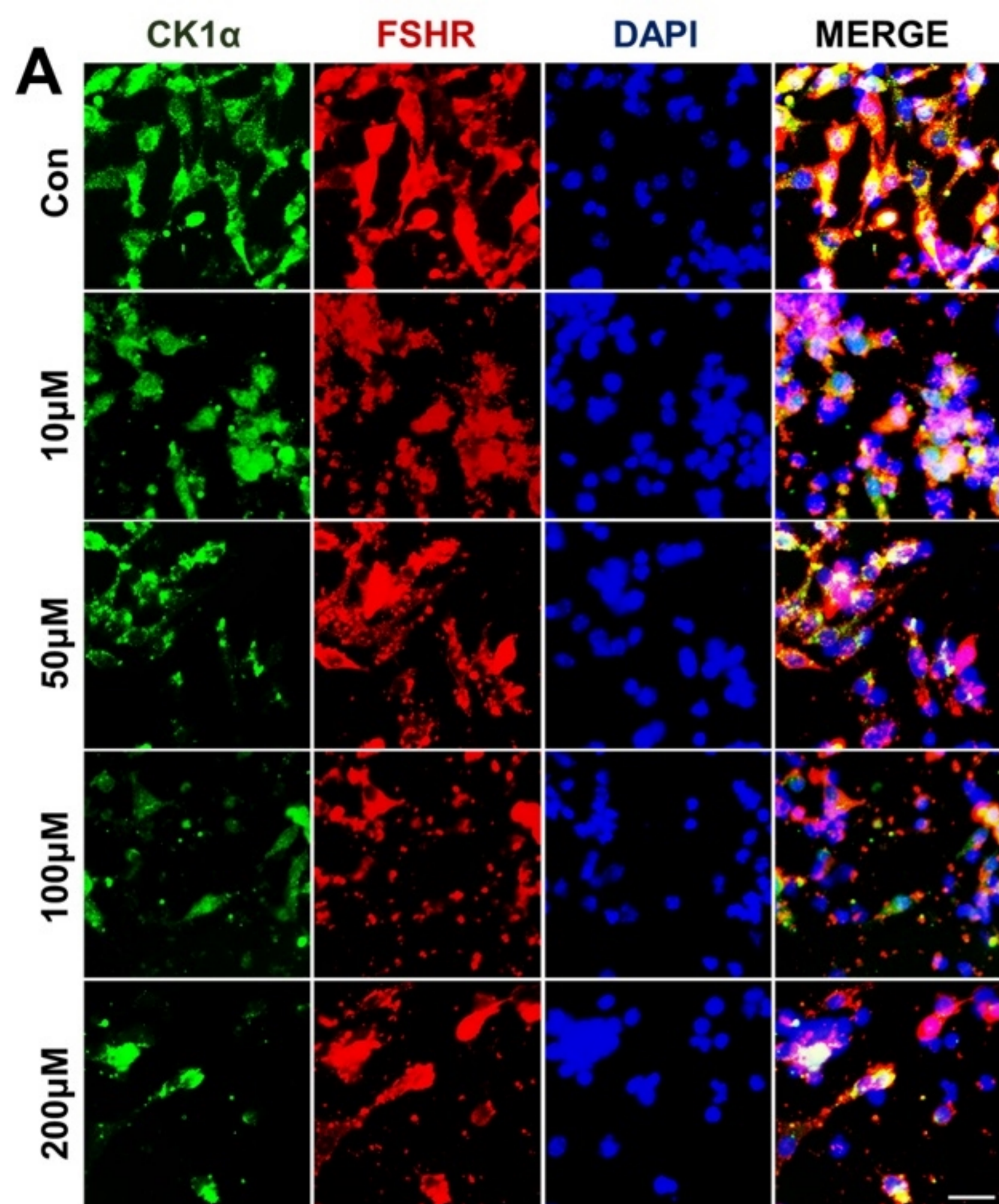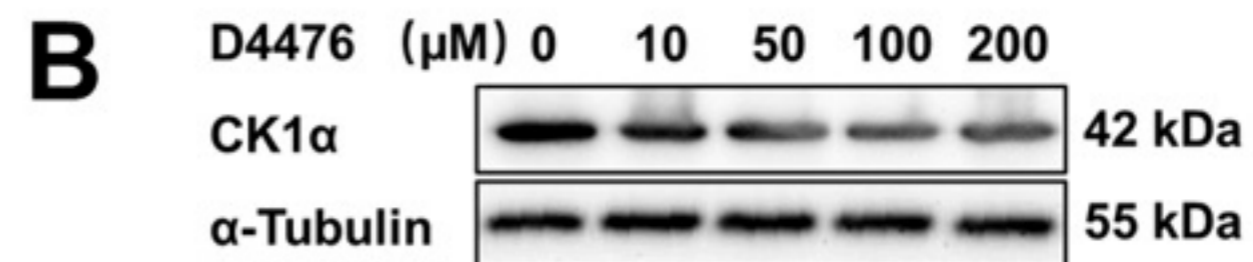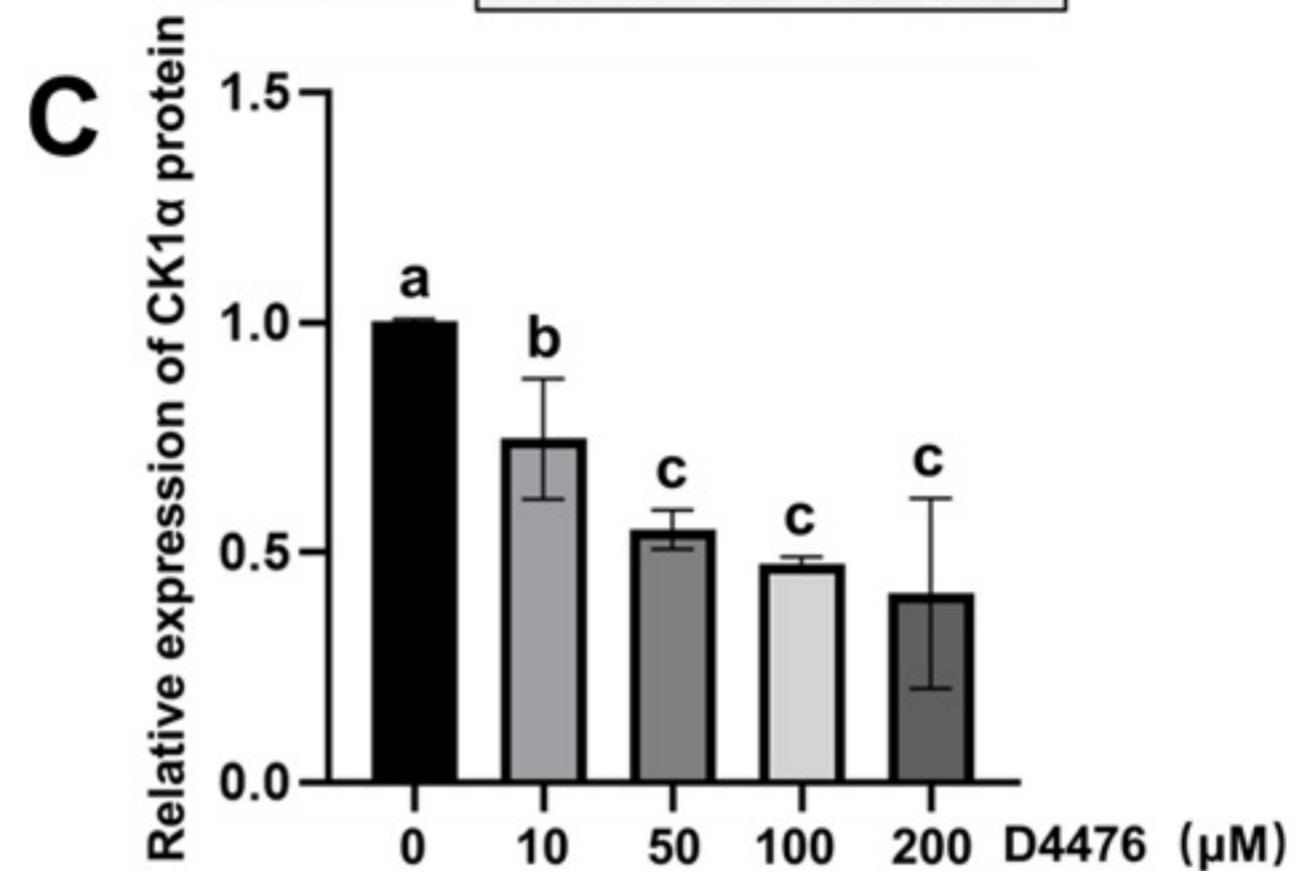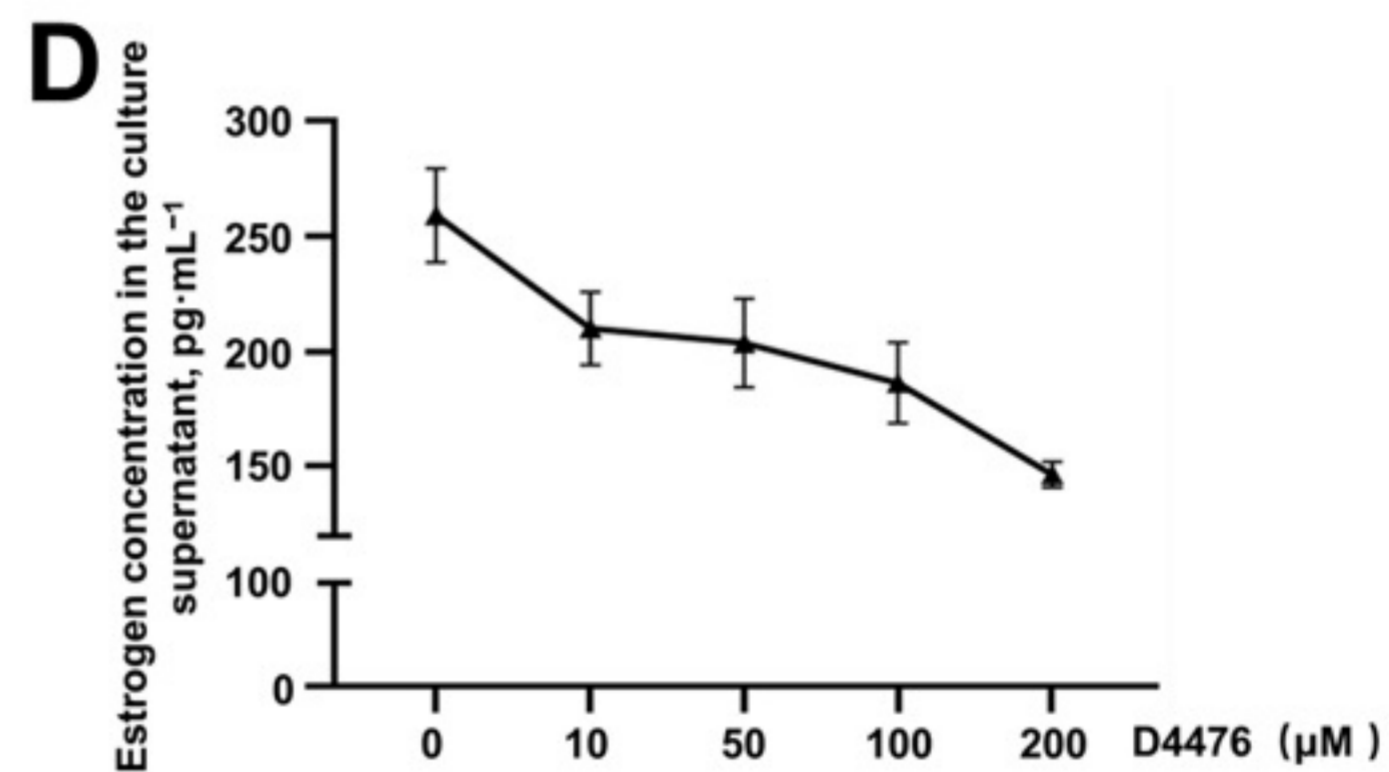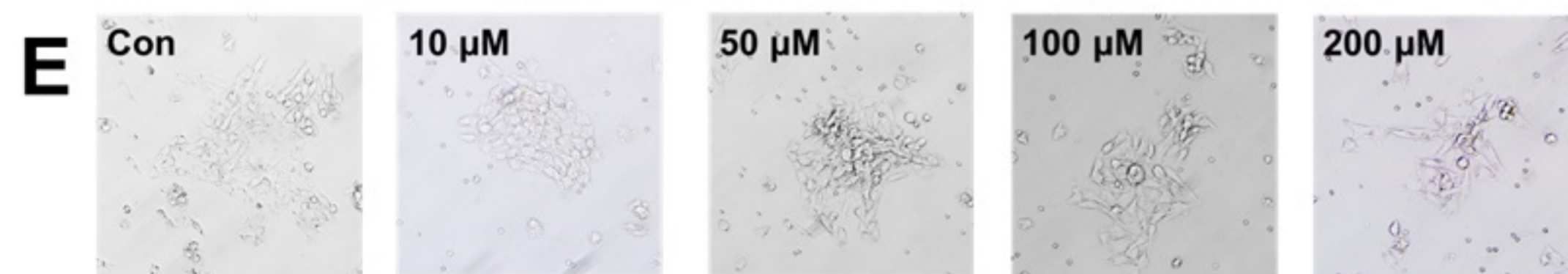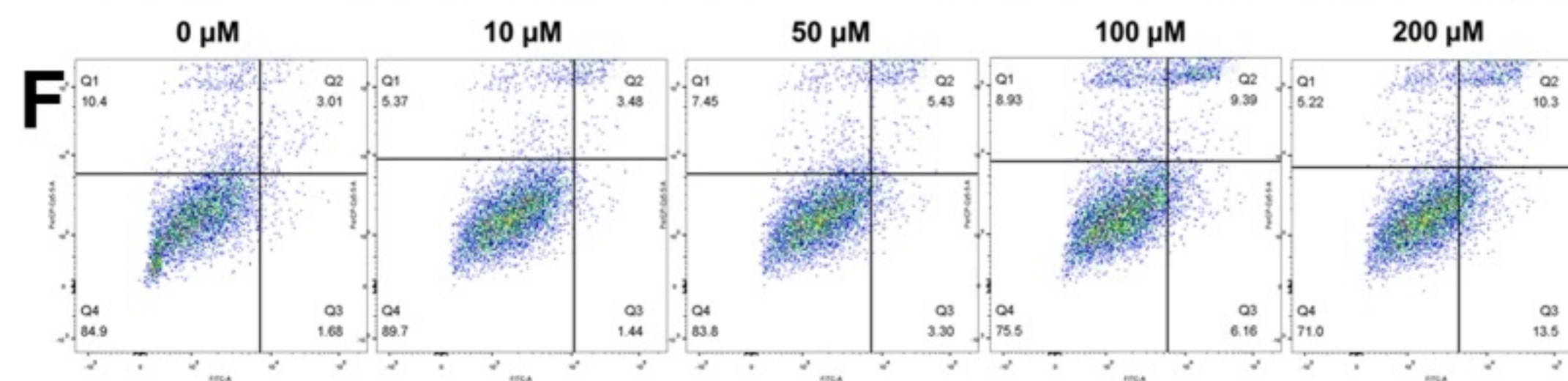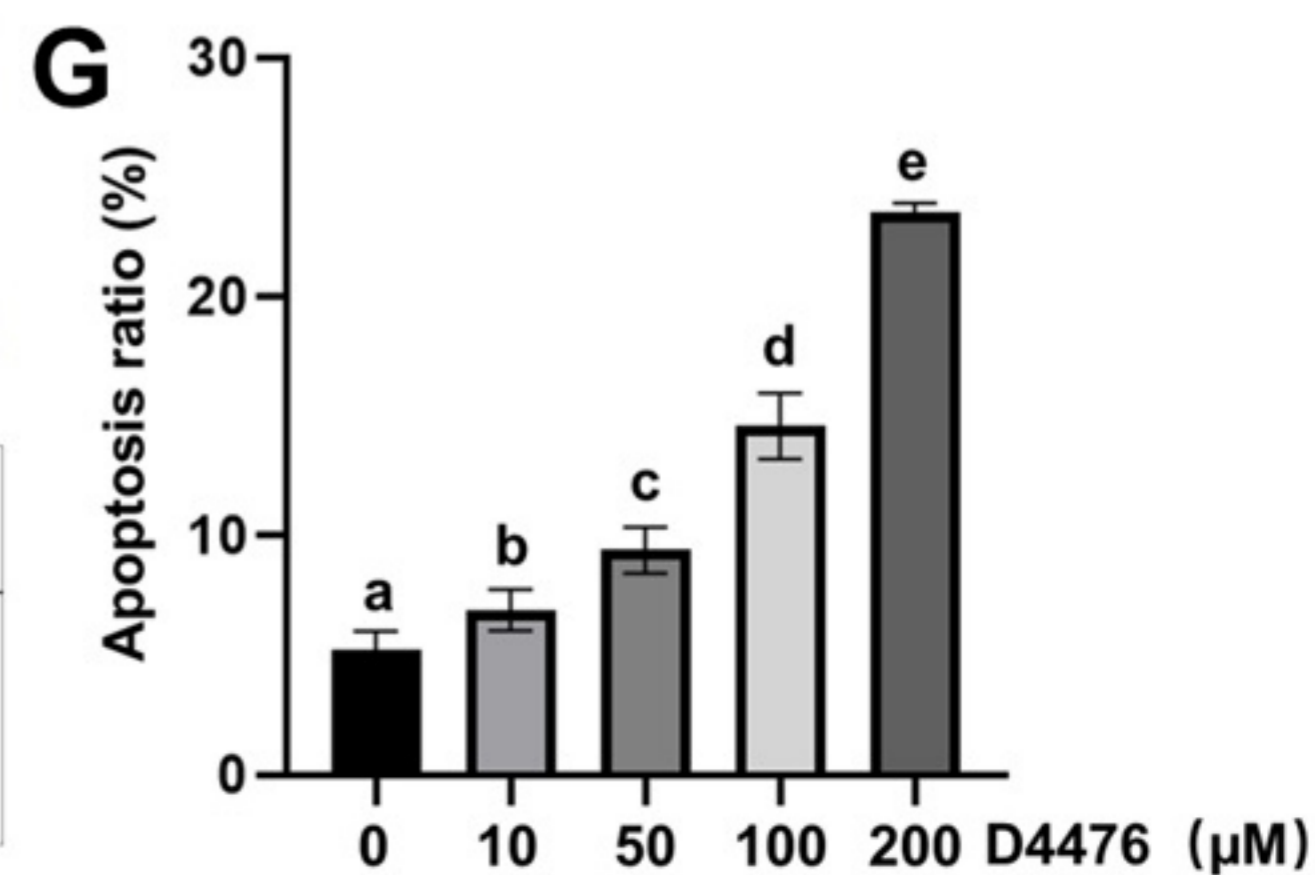

Supplement: Supplementary file 12 — Additional file 12. Figure S9. The inhibitor D4476 induces apoptosis in primary GCs from wild-type female ovaries.IF double staining for the expression of CK1αand FSHRin primary GCs from wild-type mice ovaries. DAPIwas used to stain the nucleus. Scale bar = 20 µm.Representative image of WB detecting the inhibitor efficiency of CK1α protein inside primary GCs. Relative protein levels were normalized to α-tubulin and quantified by using ImageJ software.E2 content in culture media measured by ELISA.The bright field image of primary GCs.Representative images of flow cytometry.The statistical analysis results of the total percentage of early and late apoptotic cells. Each experiment has three biological replicates. [file 12915_2024_1957_MOESM12_ESM.pdf]
