# Supplementary material for: Casein kinase 1α mediates estradiol secretion via CYP19A1 expression in mouse ovarian granulosa cells
Source: BMC Biol. 2024 Aug 26;22:176. doi: 10.1186/s12915-024-01957-3 (PMC11346181; doi:10.1186/s12915-024-01957-3)
Supplement: Supplementary file 5 — Additional file 5: Table S1. Records of the birthdates and litter sizes of the mice [file 12915_2024_1957_MOESM5_ESM.pdf]

Table S1. Records of the birthdates and litter sizes of the mice.

Group	Con		cKO	
	**Birth date**	**Litter size**	**Birthdate**	**Litter size**
Oct.	8th	9	7th	4
	23rd	7	11th	6
Nov.	6th	6	8th	6
	11th	9	22nd	5
	23rd	8		
Dec.	7th	8	4th	8
	7th	5		
	12th	7		
	8th	9		
