# Supplementary material for: Casein kinase 1α mediates estradiol secretion via CYP19A1 expression in mouse ovarian granulosa cells
Source: BMC Biol. 2024 Aug 26;22:176. doi: 10.1186/s12915-024-01957-3 (PMC11346181; doi:10.1186/s12915-024-01957-3)
Supplement: Supplementary file 6 — Additional file 6: Table S2. Records of the water and feed intake of the mice [file 12915_2024_1957_MOESM6_ESM.pdf]

Table S2. Records of the water and feed intake of the mice.

Group	Weekly water intake(mL)	Weekly feed intake(mL)	Number of mice(n)	Total weight of mice(g)	Average body weight (g)	Average daily feed intake	Average daily water intake (mL)
Con	106.51	69.25	4	76.59	19.15	2.47	3.80
Con	158.42	159.8	4	106.24	26.56	5.71	5.66
Con	83.03	47.16	2	44.81	22.41	3.37	5.93
Con	85.48	71.89	3	75.02	25.01	3.42	4.07
cKO	96.02	67.07	4	80.96	20.24	2.40	3.43
cKO	98.71	71.16	4	73.46	18.37	2.54	3.53
cKO	76.84	74.26	3	68.92	22.97	3.54	3.66
cKO	120.93	67.14	3	62.03	20.68	3.20	5.76
