# Supplementary material for: Casein kinase 1α mediates estradiol secretion via CYP19A1 expression in mouse ovarian granulosa cells
Source: BMC Biol. 2024 Aug 26;22:176. doi: 10.1186/s12915-024-01957-3 (PMC11346181; doi:10.1186/s12915-024-01957-3)
Supplement: Supplementary file 7 — Additional file 7: Table S3. Reproductive fertility performance of the mice [file 12915_2024_1957_MOESM7_ESM.pdf]

Table S3. Reproductive fertility performance of the mice.

Female ♀	Male ♂	Plugged rate (%)	Pregnant rate (%)	Total number born (n)
Con	Csnk1α1flox/flox	83.3 ± 8.33^{a}	58.3 ± 8.33^{a}	7.6 ± 0.47^{a}
cKO	Csnk1α1flox/flox	75.0 ± 14.3^{b}	41.6 ± 8.33^{b}	5.8 ± 0.66^{b}
