# Supplementary material for: Casein kinase 1α mediates estradiol secretion via CYP19A1 expression in mouse ovarian granulosa cells
Source: BMC Biol. 2024 Aug 26;22:176. doi: 10.1186/s12915-024-01957-3 (PMC11346181; doi:10.1186/s12915-024-01957-3)
Supplement: Supplementary file 13 — Additional file 13. Table 1. Primers used for RT-PCR [file 12915_2024_1957_MOESM13_ESM.pdf]

Table 1. Primers used for RT-PCR

Name	Sequence
Csnk1a1	F- CTGGCTCTTTCGGGGACATT
	R- TGCTCTCGTACAGCAACTGG
Cyp19a1	F- TTGGAAATGCTGAACCCCAT
	R- CAAGAATCTGCCATGGGAAA
Raf1	F- GCTAATTGACATTGCCCGACA
	R- TTCAACCTGCTGAGAACCAC
Gapdh	F- GGTTGTCTCCTGCGACTTCA
	R- GGGTGGTCCAGGGTTTCTTA
