# Supplementary material for: Casein kinase 1α mediates estradiol secretion via CYP19A1 expression in mouse ovarian granulosa cells
Source: BMC Biol. 2024 Aug 26;22:176. doi: 10.1186/s12915-024-01957-3 (PMC11346181; doi:10.1186/s12915-024-01957-3)

Fig.1

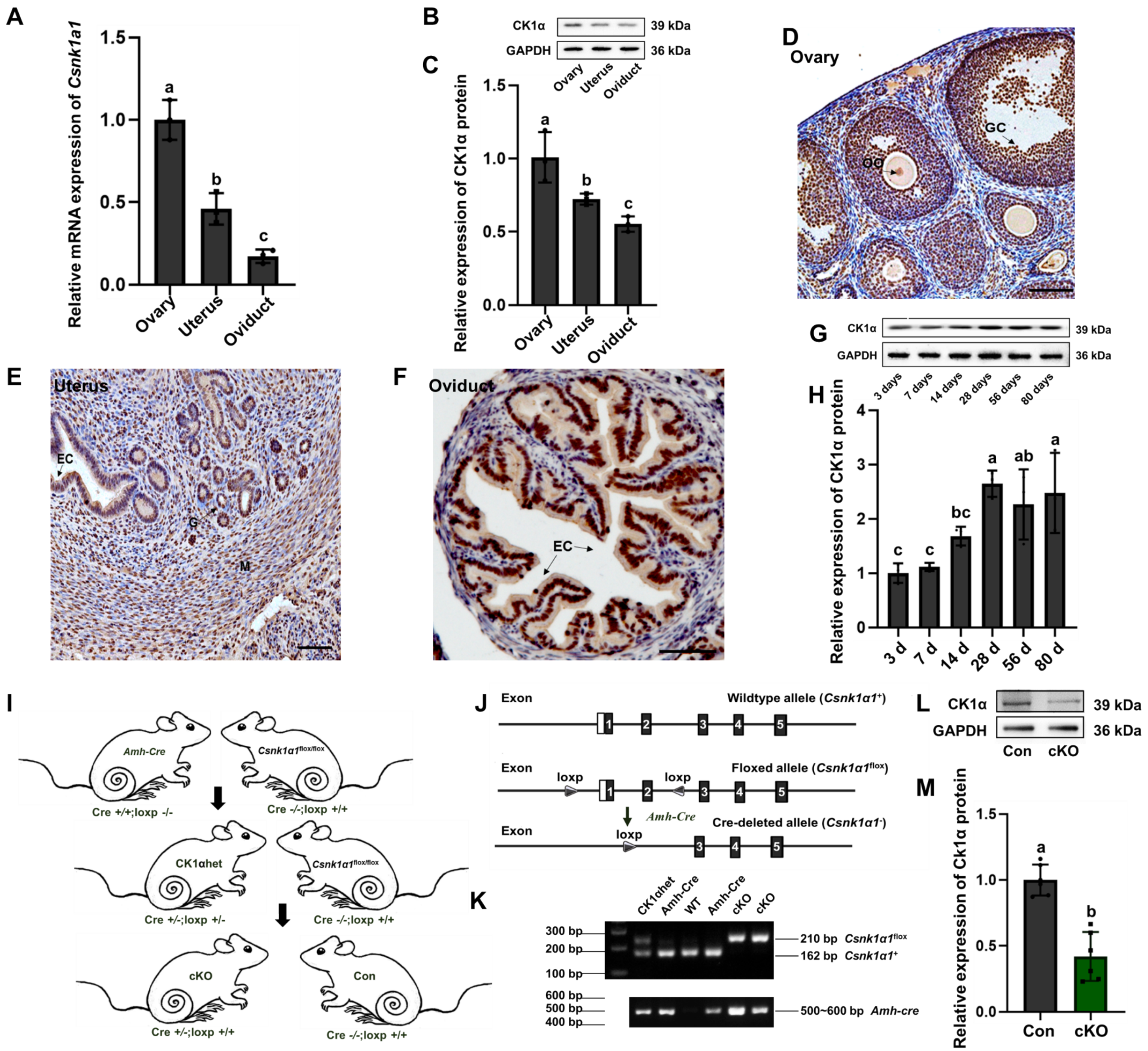
A
Relative mRNA expression of Csnk1a1
Ovary
Uterus
Oviduct
B
CK1α
GAPDH
39 kDa
36 kDa
C
Relative expression of CK1α protein
D
Ovary
GC
OO
E
Uterus
EC
G
M
F
Oviduct
EC
G
CK1α
GAPDH
3 days
7 days
14 days
28 days
56 days
80 days
H
Relative expression of CK1α protein
3 d
7 d
14 d
28 d
56 d
80 d
I
Amh-Cre
Csnk1α1flox/flox
Cre +/+;loxp -/-
Cre -/-;loxp +/+
CK1αhet
Cre +/-;loxp +/-
cKO
Con
Cre +/-;loxp +/+
J
Exon
Wildtype allele (Csnk1α1+)
Floxed allele (Csnk1α1flox)
Cre-deleted allele (Csnk1α1-)
loxp
Amh-Cre
K
CK1αhet
Amh-Cre
WT
cKO
300 bp
200 bp
100 bp
600 bp
500 bp
400 bp
210 bp Csnk1α1flox
162 bp Csnk1α1+
500~600 bp Amh-cre
L
CK1α
GAPDH
Con
cKO
M
Relative expression of Ck1α protein

Fig.1a

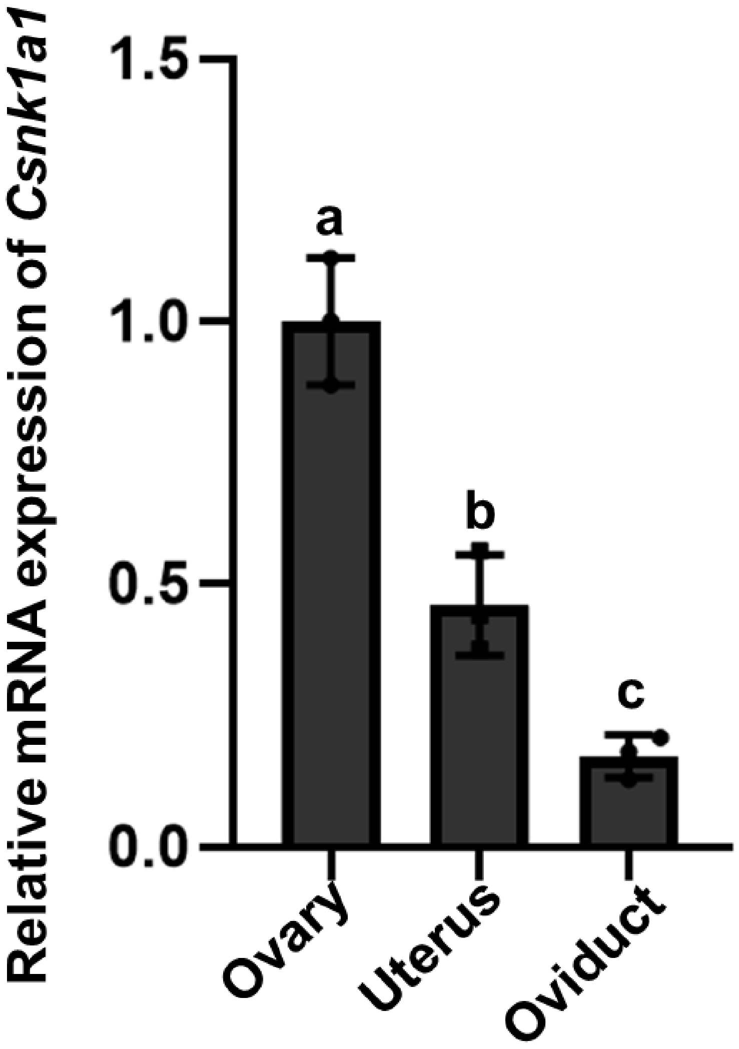
Relative mRNA expression of *Csnk1a1*
1.5
1.0
0.5
0.0
a
b
c
Ovary
Uterus
Oviduct

Fig.1b, c

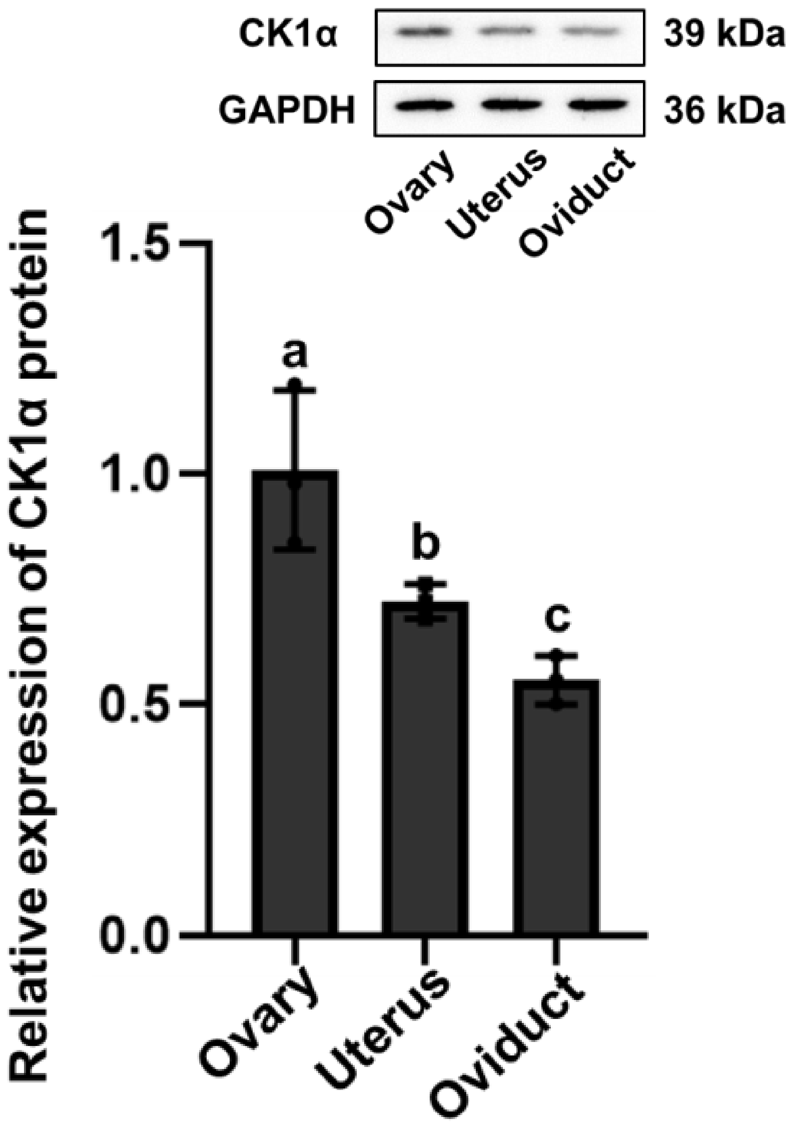
CK1α
39 kDa
GAPDH
36 kDa
Ovary
Uterus
Oviduct
Relative expression of CK1α protein
1.5
1.0
0.5
0.0
a
b
c
Ovary
Uterus
Oviduct

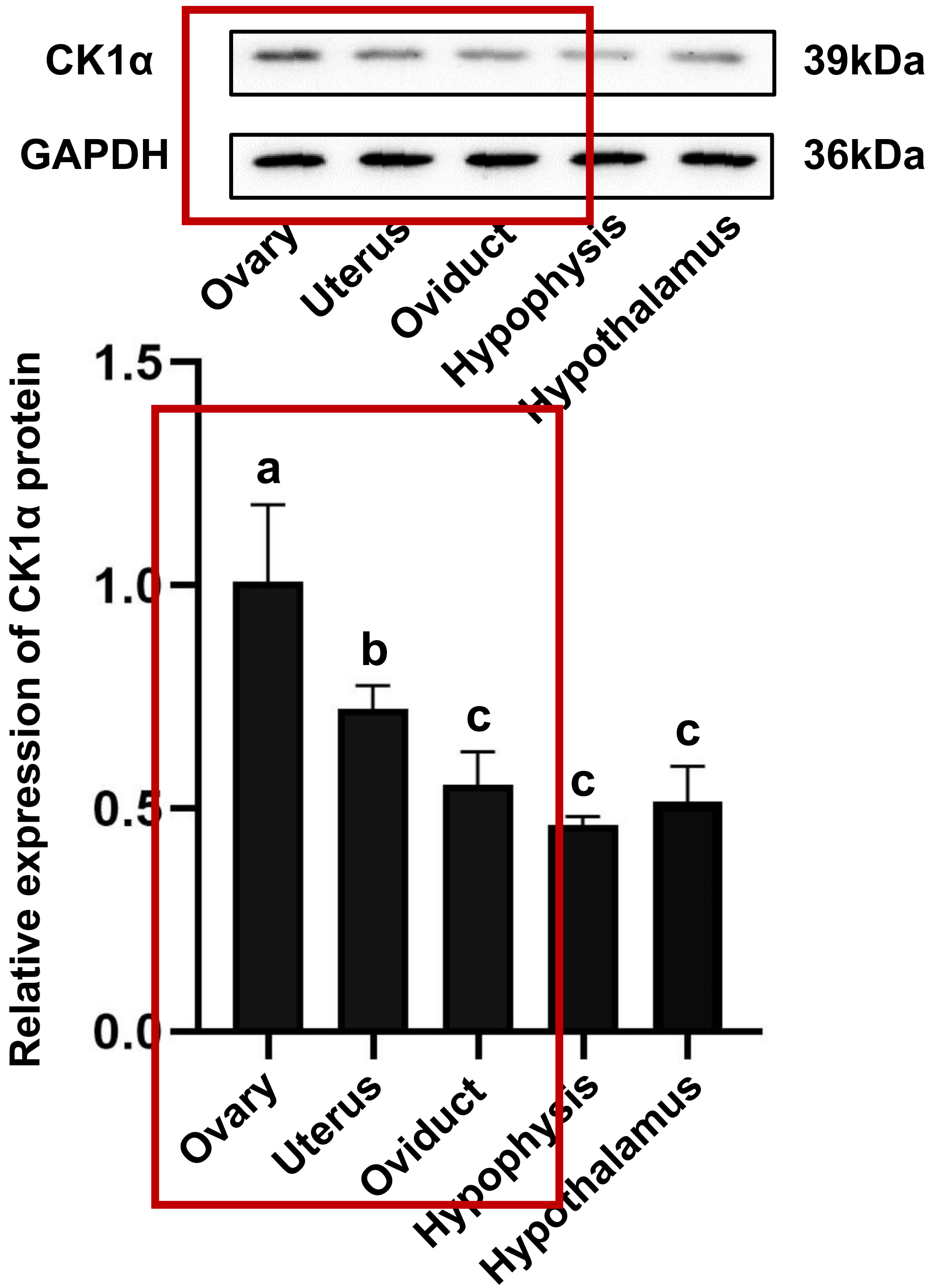
CK1α
39kDa
GAPDH
36kDa
Ovary
Uterus
Oviduct
Hypophysis
Hypothalamus
Relative expression of CK1α protein
1.5
1.0
0.5
0.0
a
b
c
c
c
Ovary
Uterus
Oviduct
Hypophysis
Hypothalamus

Fig.1d

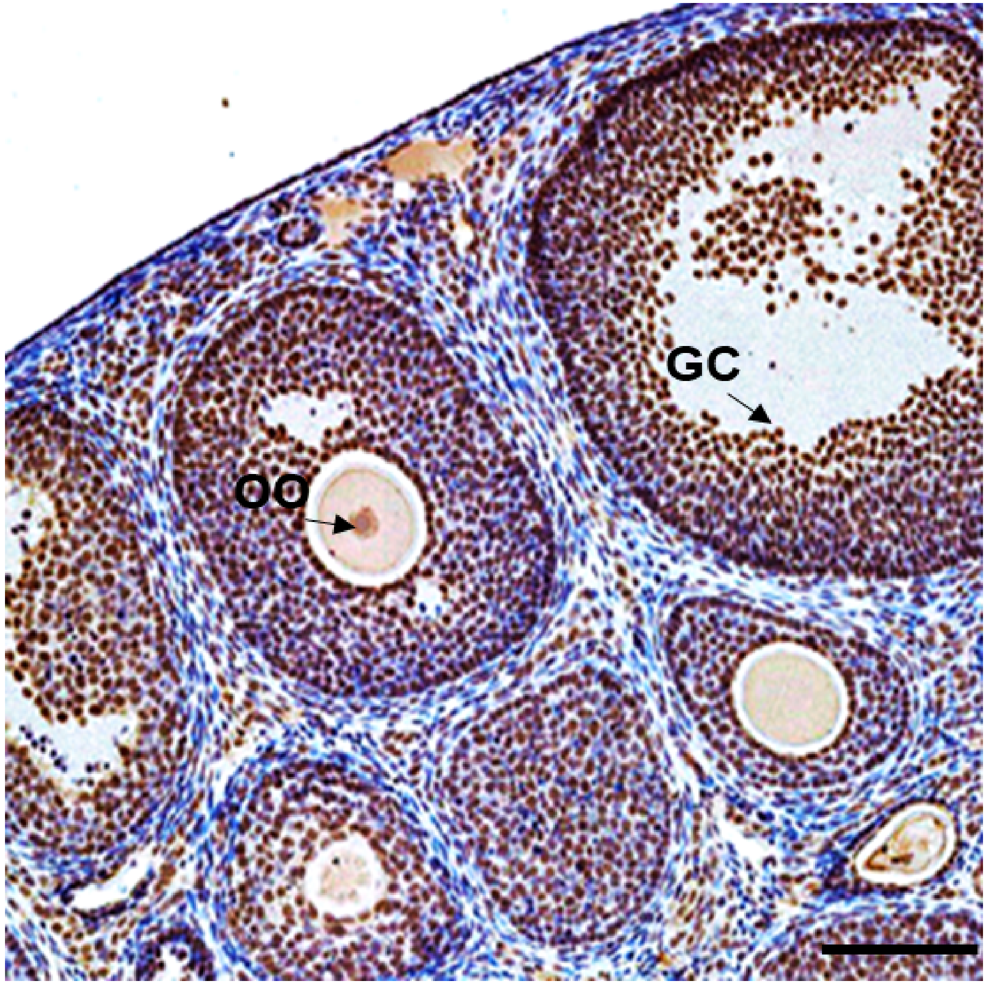

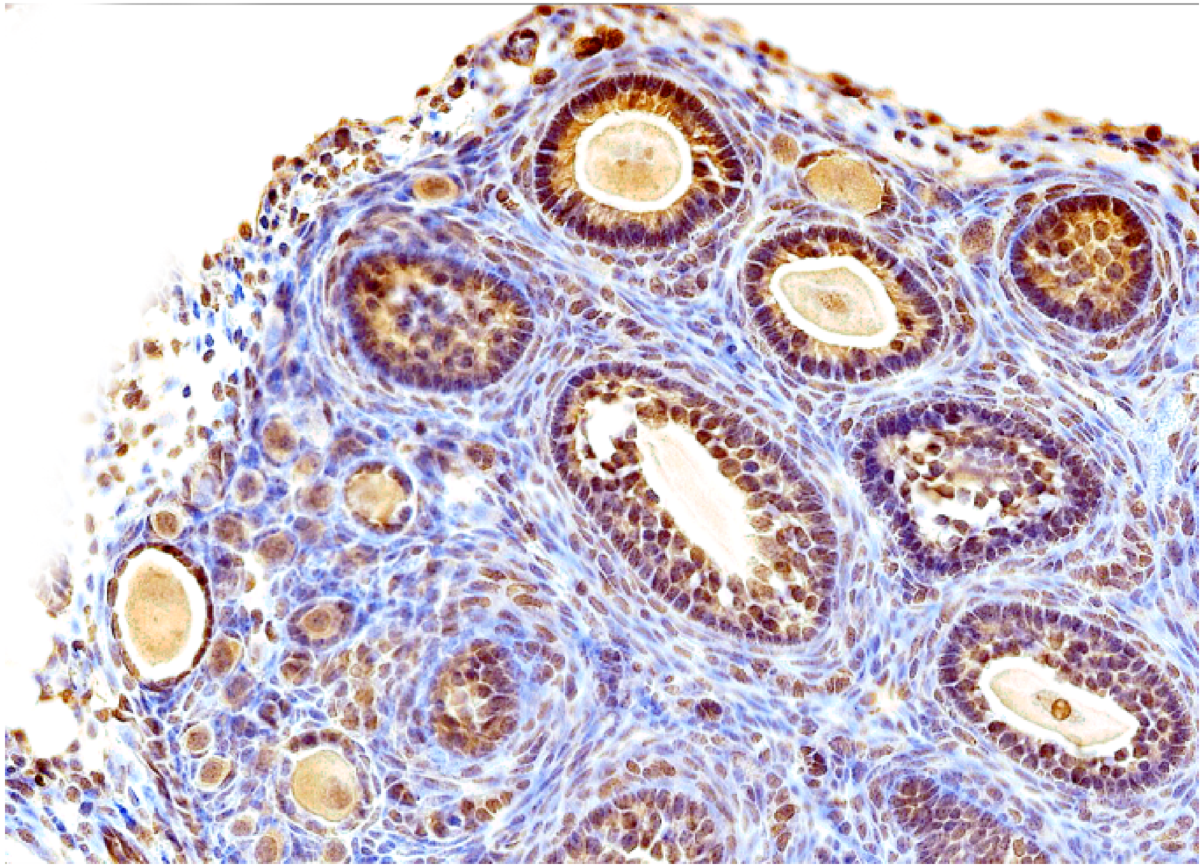

Fig.1e

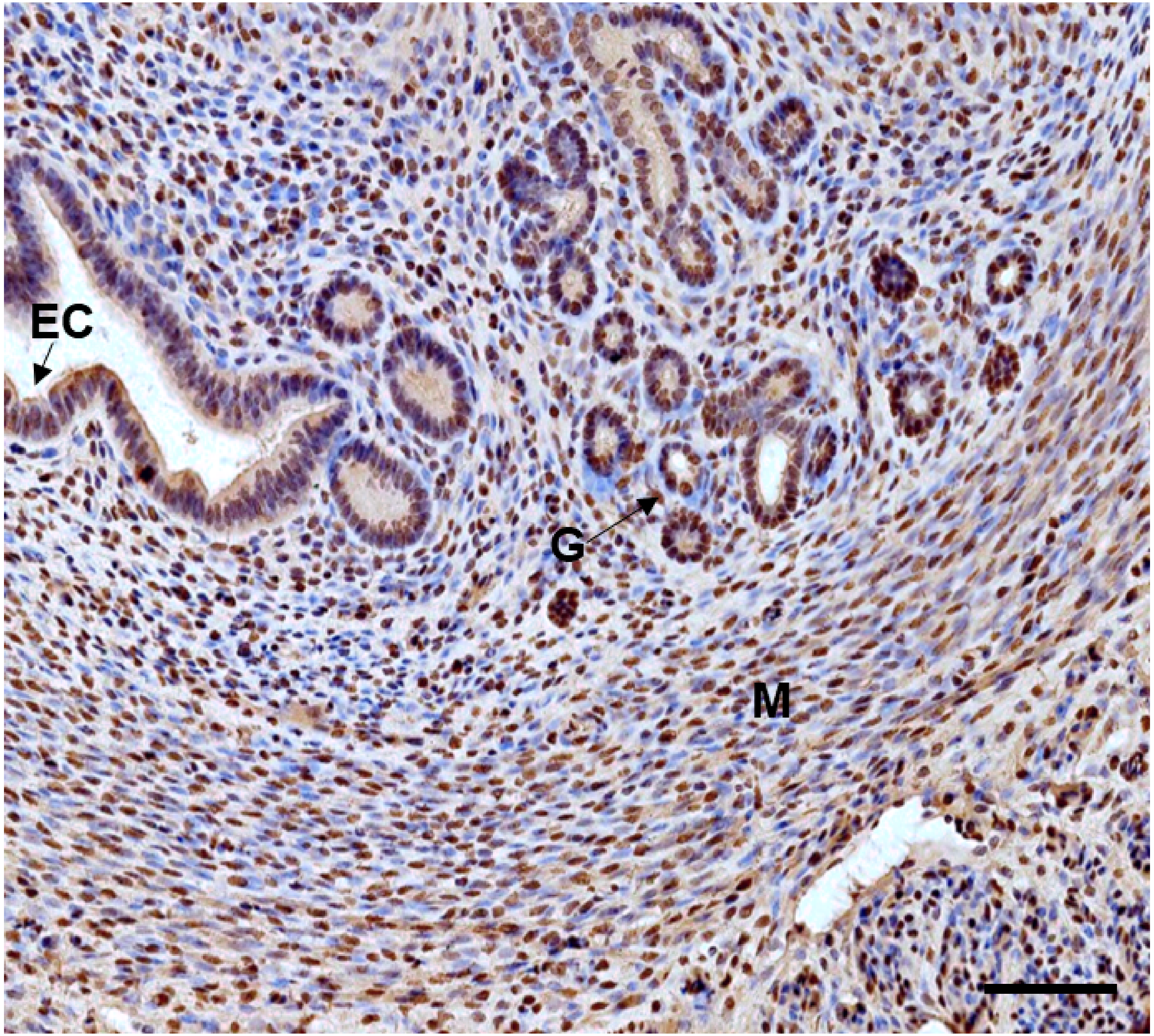

Fig.1f

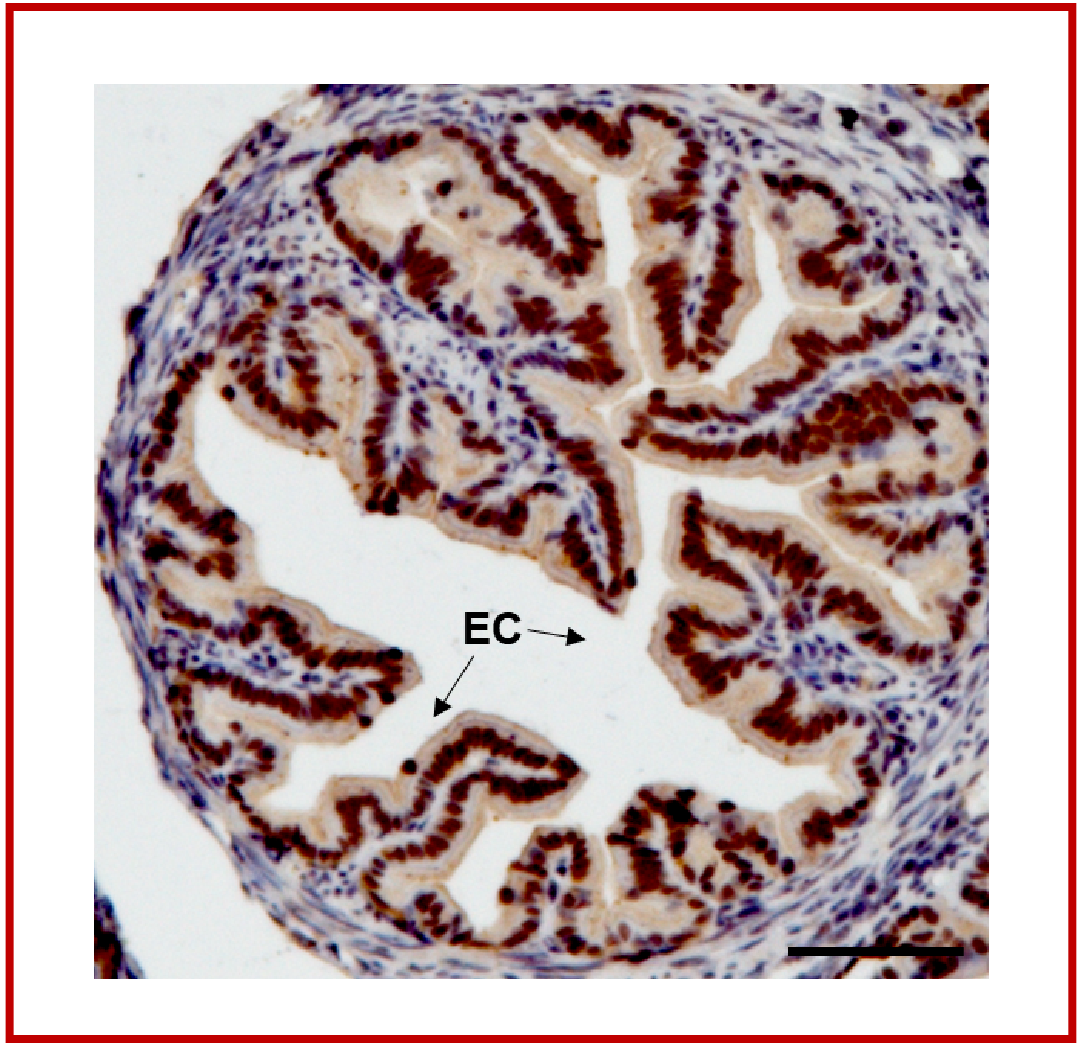

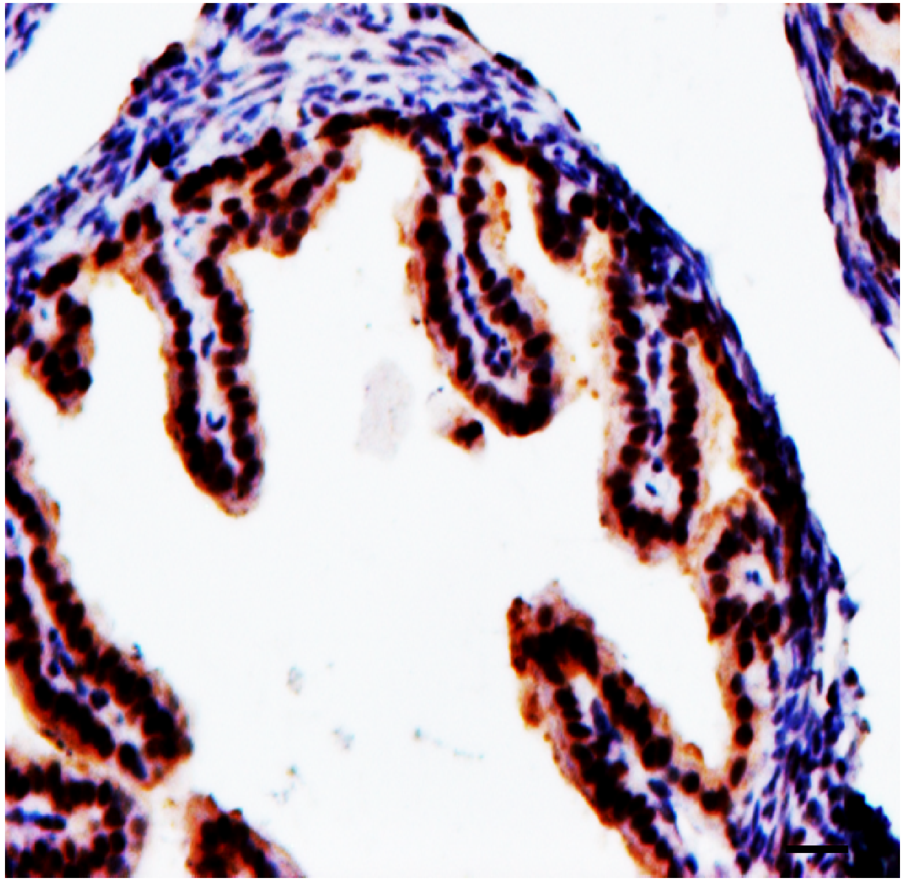

Fig.1g, k

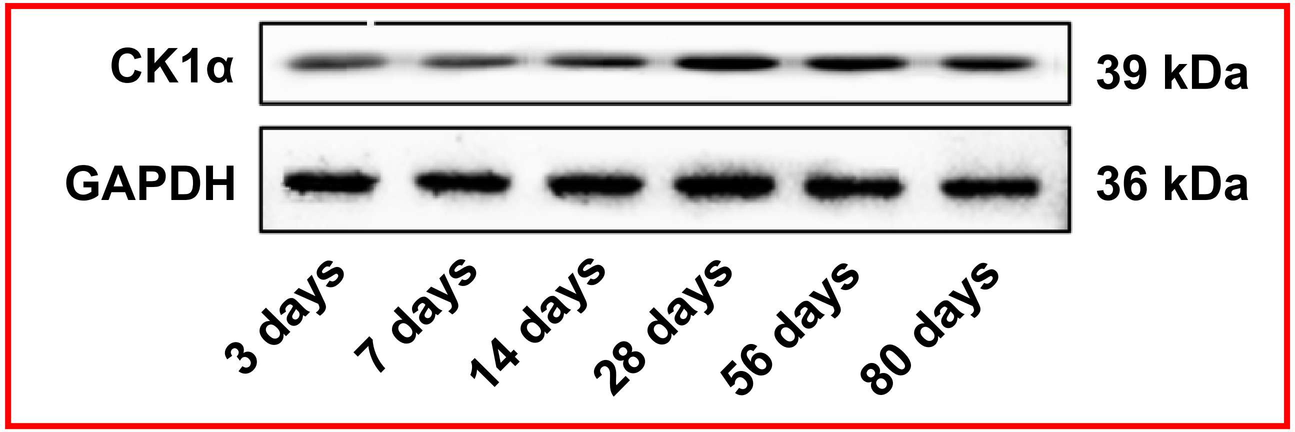
CK1α
39 kDa
GAPDH
36 kDa
3 days
7 days
14 days
28 days
56 days
80 days

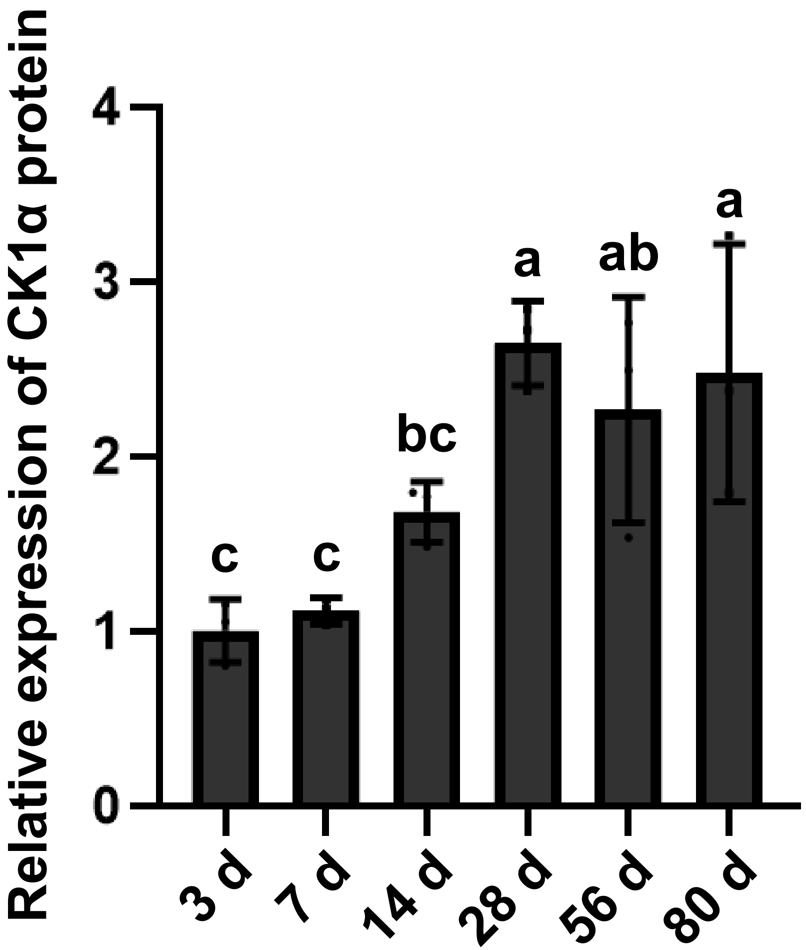
Relative expression of CK1α protein
4
3
2
1
0
c
c
bc
a
ab
a
3 d
7 d
14 d
28 d
56 d
80 d

Fig.1I

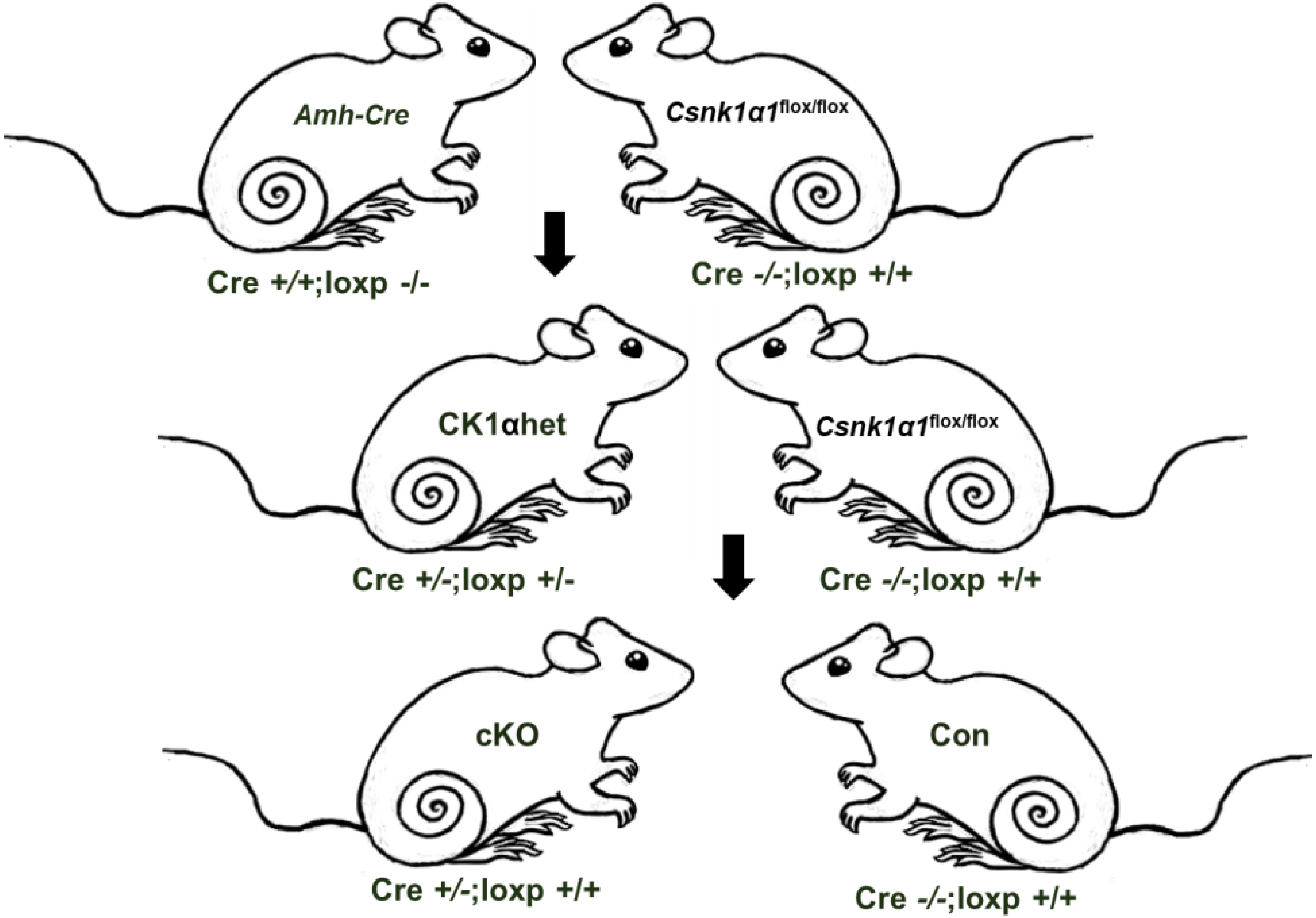

Amh-Cre
*Csnk1α1*flox/flox
Cre +/+;loxp -/-
Cre -/-;loxp +/+
CK1αhet
*Csnk1α1*flox/flox
Cre +/-;loxp +/-
Cre -/-;loxp +/+
cKO
Con
Cre +/-;loxp +/+
Cre -/-;loxp +/+

Fig.1j

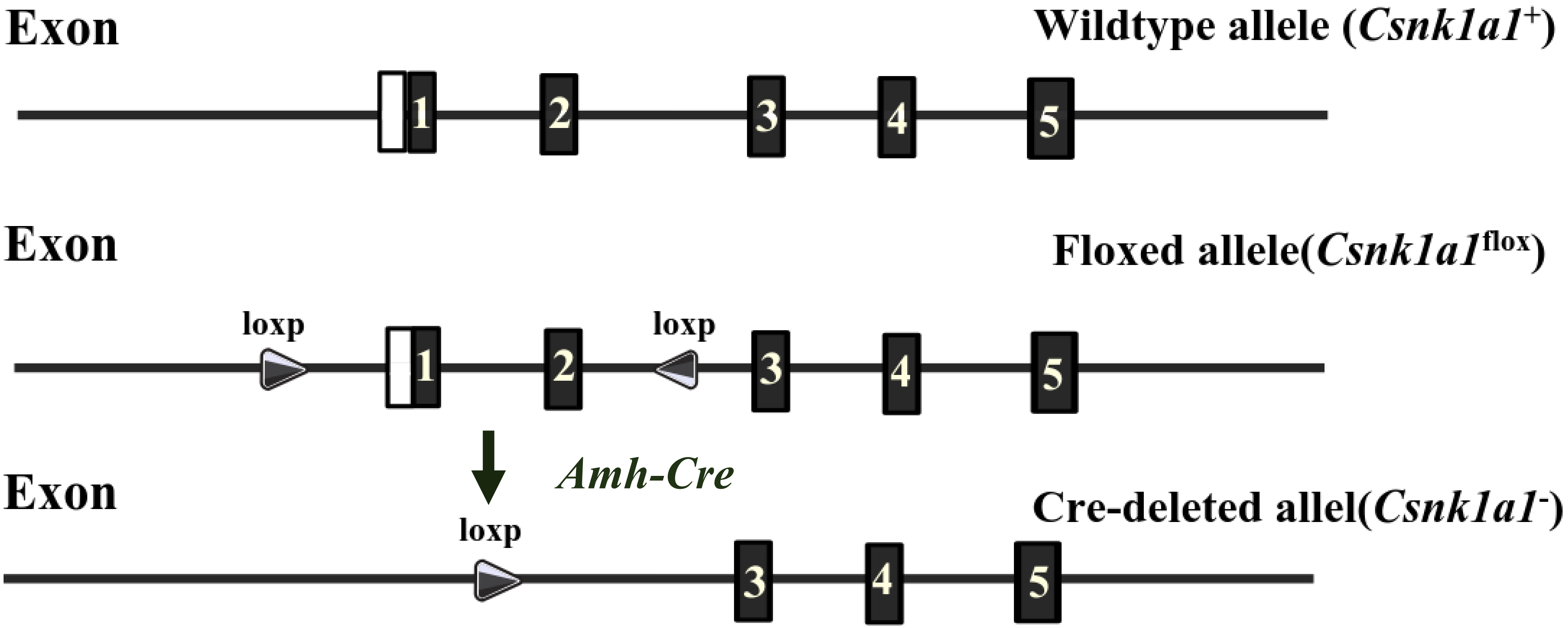
Exon
Wildtype allele ($Csnk1a1^{+}$)
1
2
3
4
5
Exon
Floxed allele($Csnk1a1^{flox}$)
loxp
loxp
1
2
3
4
5
Amh-Cre
Exon
Cre-deleted allel($Csnk1a1^{-}$)
loxp
3
4
5

Fig.1k

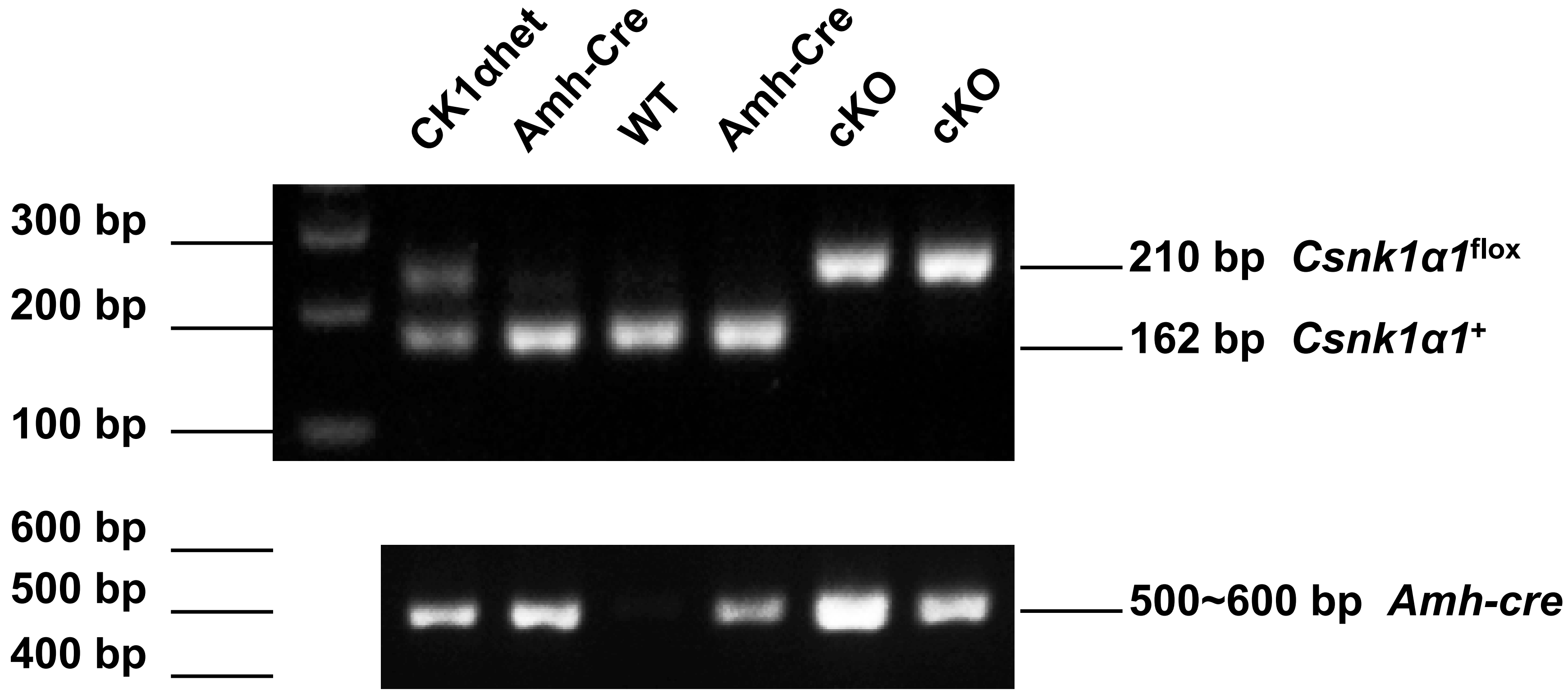

CK1αhet
Amh-Cre
WT
Amh-Cre
cKO
cKO
300 bp
200 bp
100 bp
210 bp Csnk1α1flox
162 bp Csnk1α1+
600 bp
500 bp
400 bp
500~600 bp Amh-cre

Fig.1k

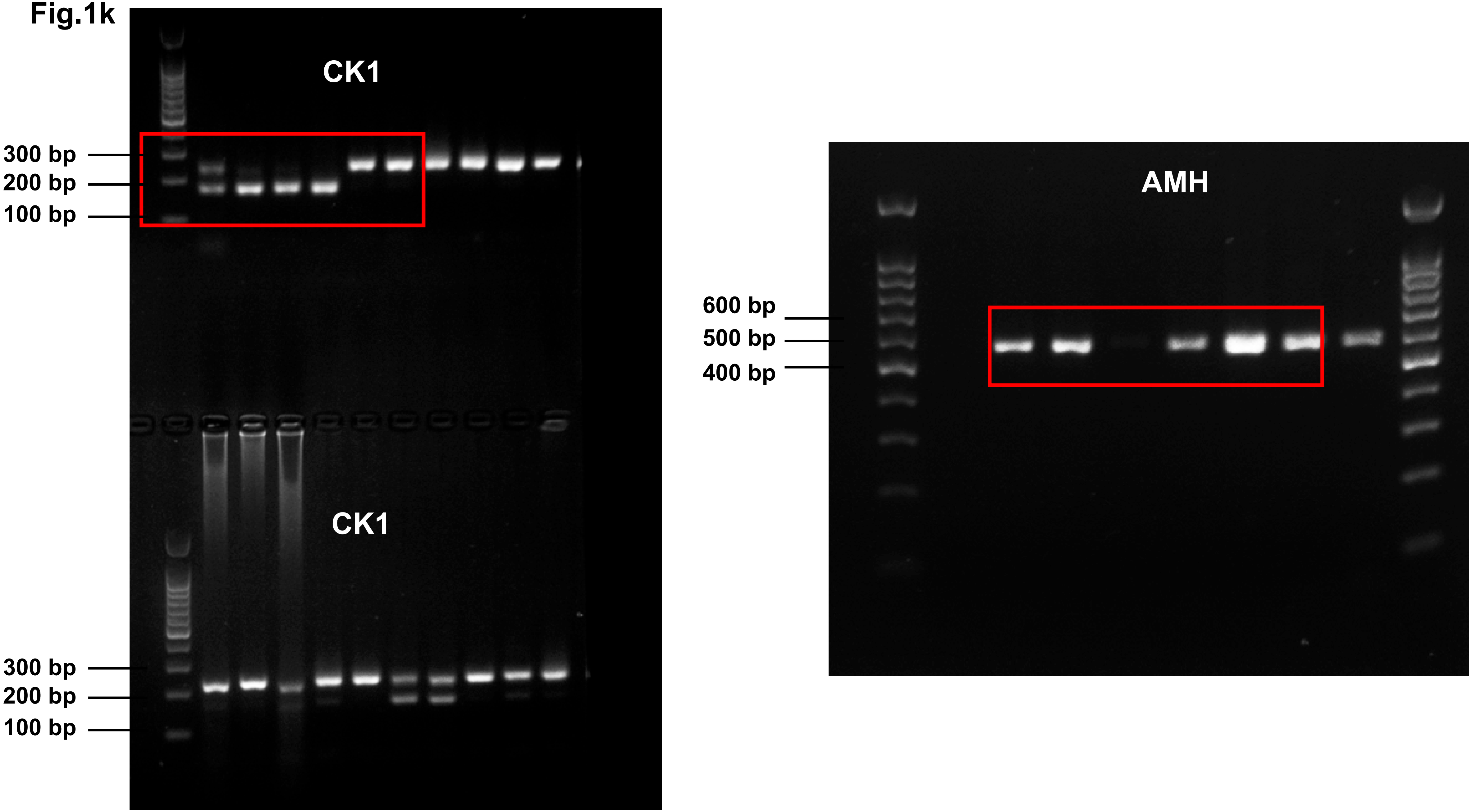
CK1
300 bp
200 bp
100 bp
CK1
300 bp
200 bp
100 bp
AMH
600 bp
500 bp
400 bp

Fig.1l，m

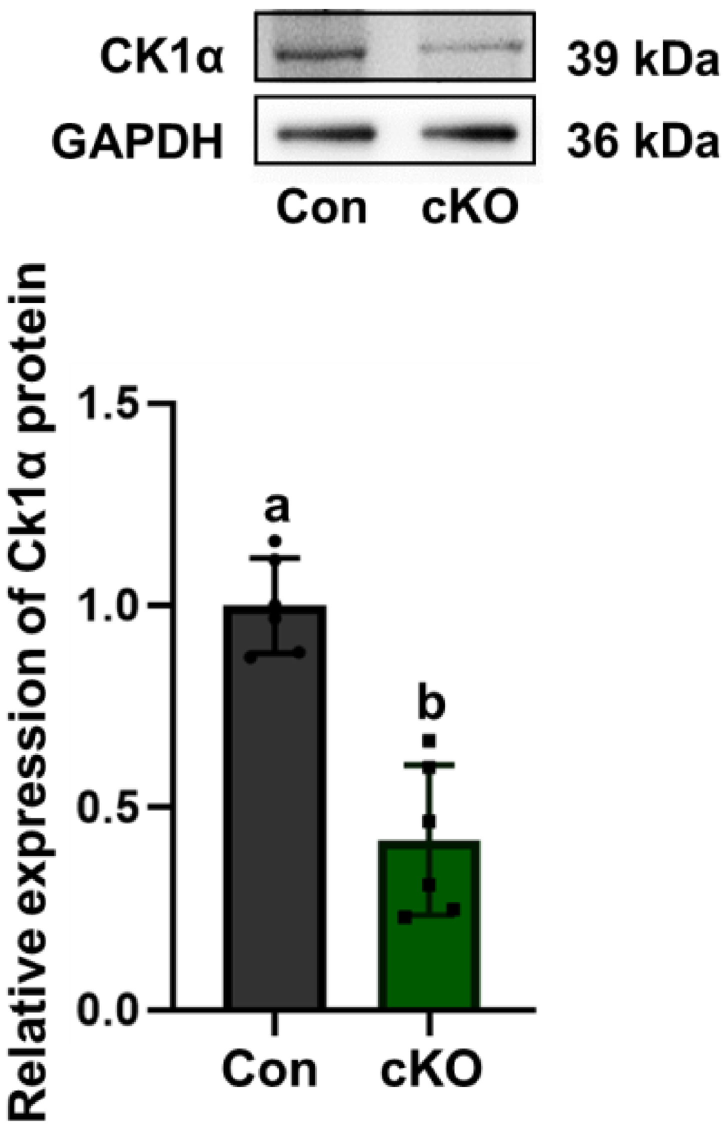
CK1α
39 kDa
GAPDH
36 kDa
Con
cKO
Relative expression of Ck1α protein
1.5
1.0
0.5
0.0
a
b
Con
cKO

Fig.1l， m

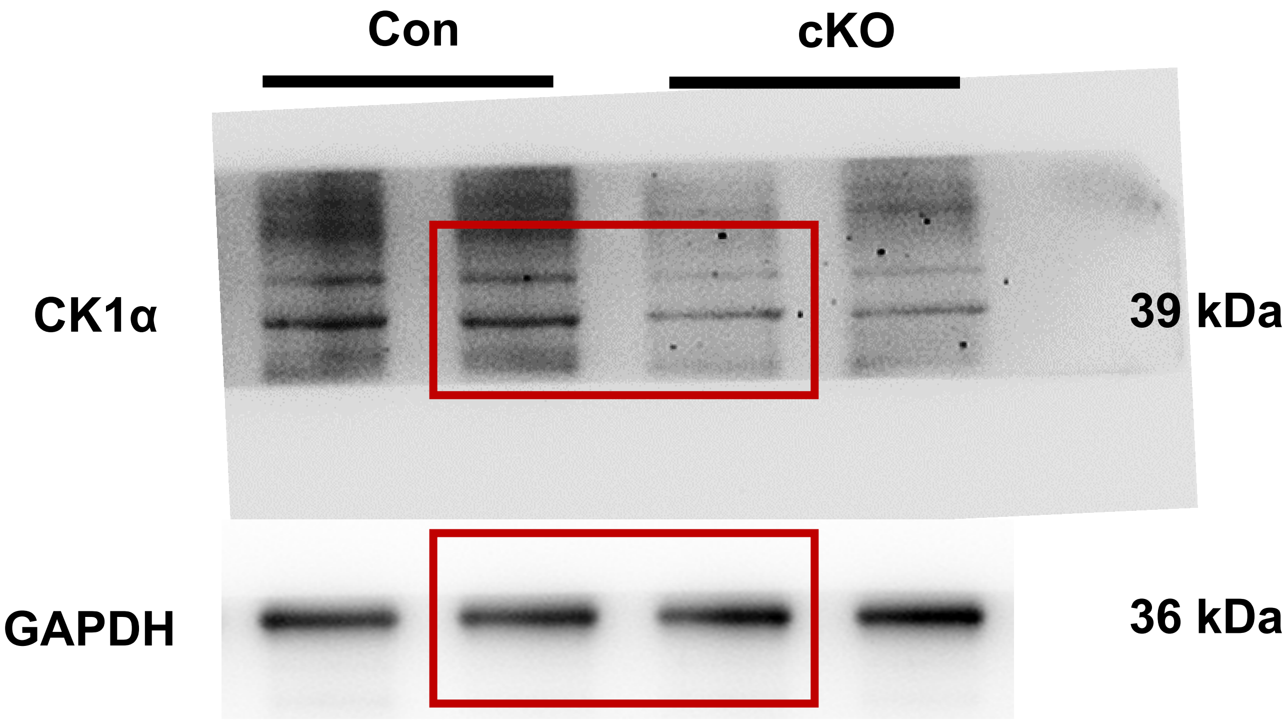

Fig.2

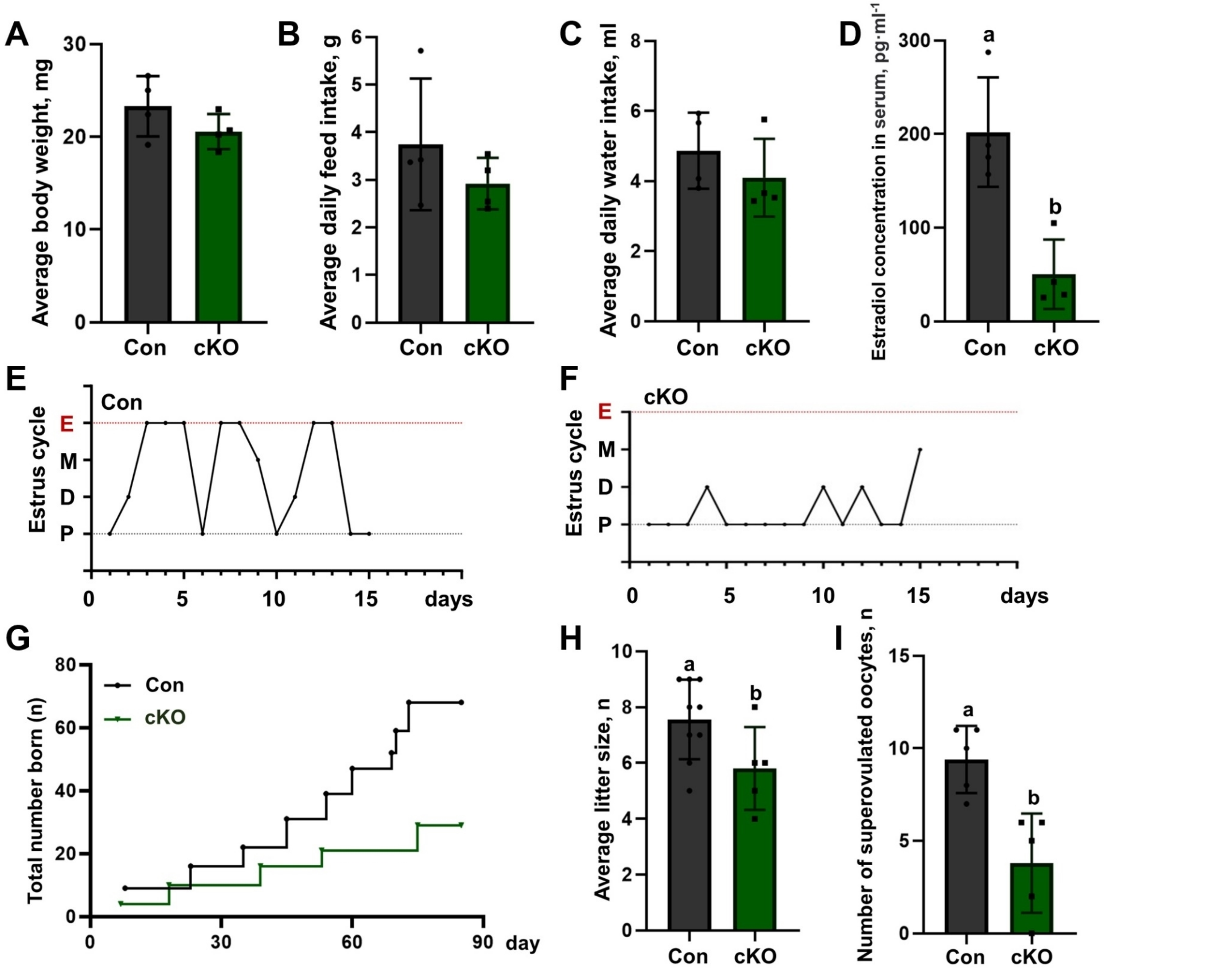
A
Average body weight, mg
Con
cKO
B
Average daily feed intake, g
Con
cKO
C
Average daily water intake, ml
Con
cKO
D
Estradiol concentration in serum, pg·ml-1
a
b
Con
cKO
E
Con
Estrus cycle
E
M
D
P
days
F
cKO
Estrus cycle
E
M
D
P
days
G
Total number born (n)
Con
cKO
day
H
Average litter size, n
a
b
Con
cKO
I
Number of superovulated oocytes, n
a
b
Con
cKO

Fig.2a-c

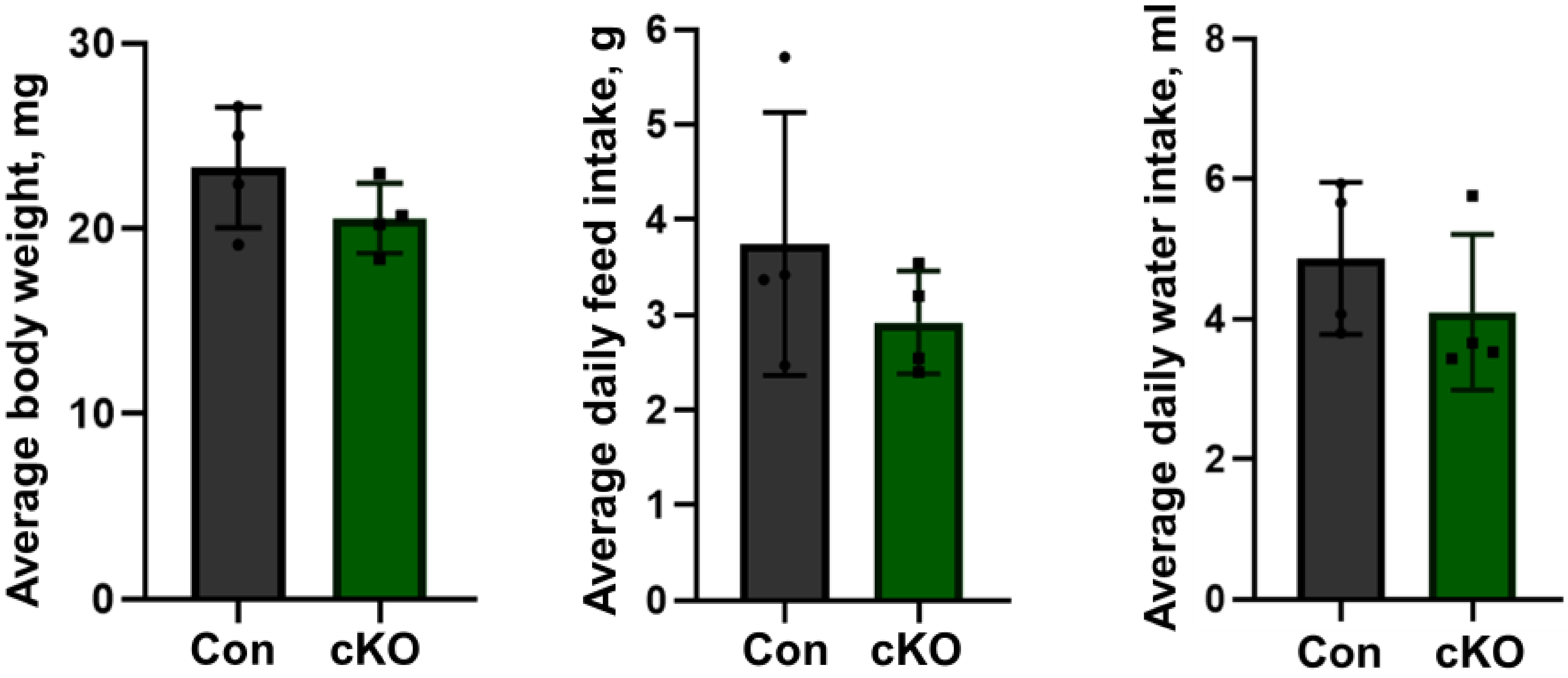

Average body weight, mg
30
20
10
0
Con
cKO
Average daily feed intake, g
6
5
4
3
2
1
0
Con
cKO
Average daily water intake, ml
8
6
4
2
0
Con
cKO

Fig.2a-c

Group	Weekly water intake (ml)	Weekly feed intake (mL)	Number of mice (n)	Total weight of mice (g)	Average body weight (g)	Average daily feed intake	Average daily water intake (mL)
Con	106.51	69.25	4	76.59	19.15	2.47	3.80
Con	158.42	159.8	4	106.24	26.56	5.71	5.66
Con	83.03	47.16	2	44.81	22.41	3.37	5.93
Con	85.48	71.89	3	75.02	25.01	3.42	4.07
cKO	96.02	67.07	4	80.96	20.24	2.40	3.43
cKO	98.71	71.16	4	73.46	18.37	2.54	3.53
cKO	76.84	74.26	3	68.92	22.97	3.54	3.66
cKO	120.93	67.14	3	62.03	20.68	3.20	5.76

Fig.2d

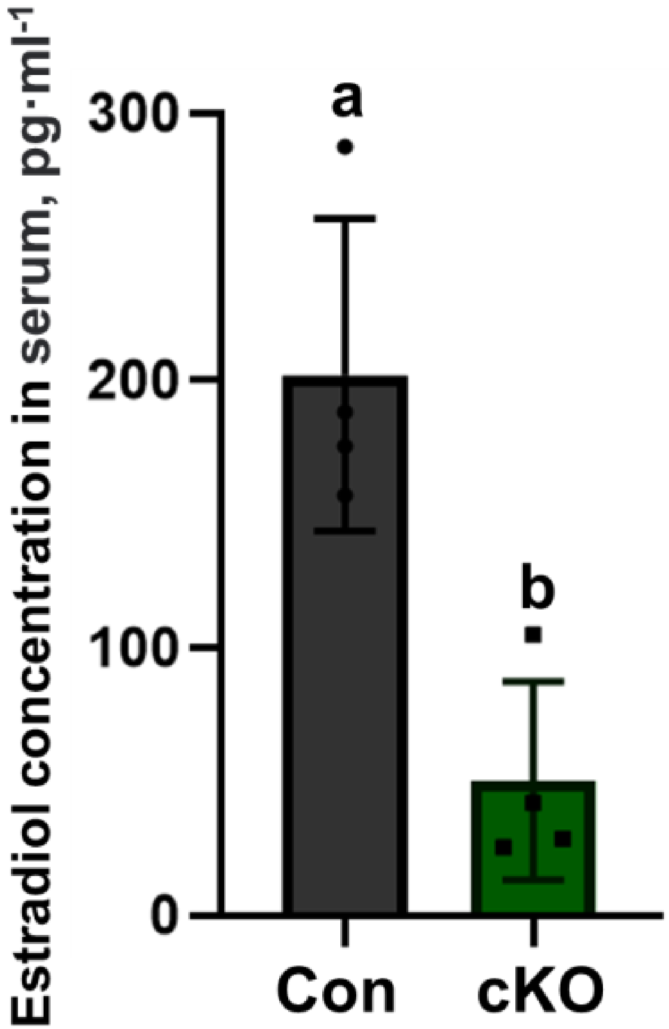
Estradiol concentration in serum, pg·ml-1
300
200
100
0
a
b
Con
cKO

Fig.2e

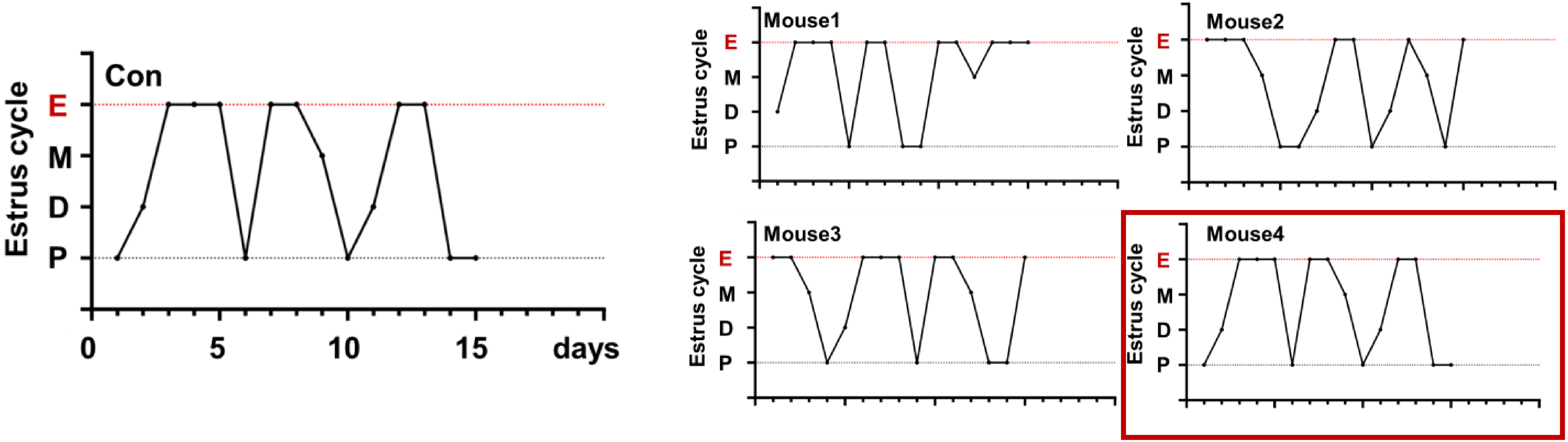
Con
Estrus cycle
E
M
D
P
0
5
10
15
days
Mouse1
Mouse2
Mouse3
Mouse4

Fig.2f

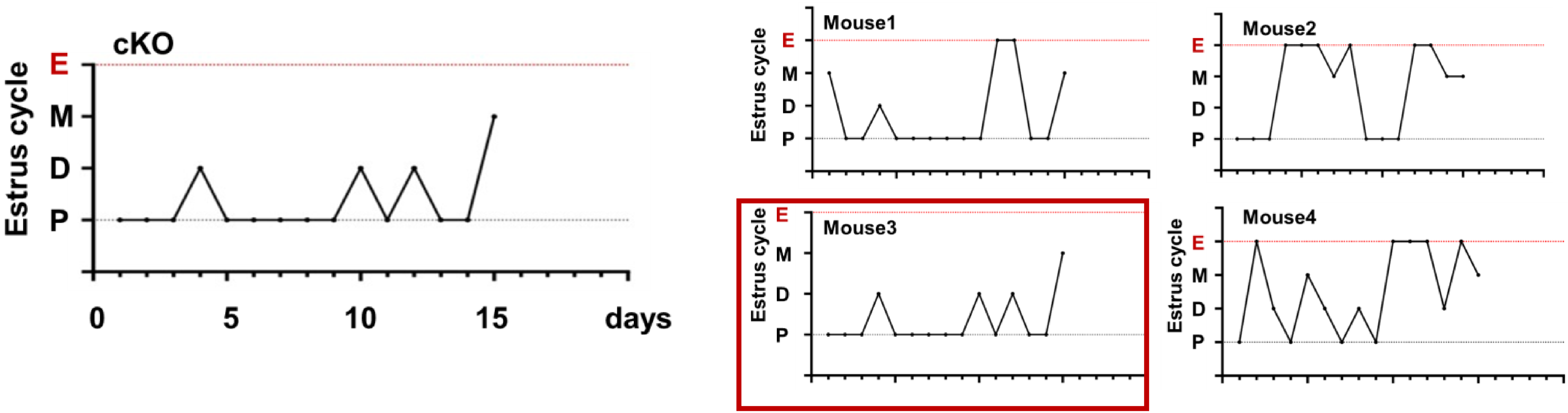

cKO
Estrus cycle
E
M
D
P
0
5
10
15
days
Mouse1
Mouse2
Mouse3
Mouse4

Fig.2g

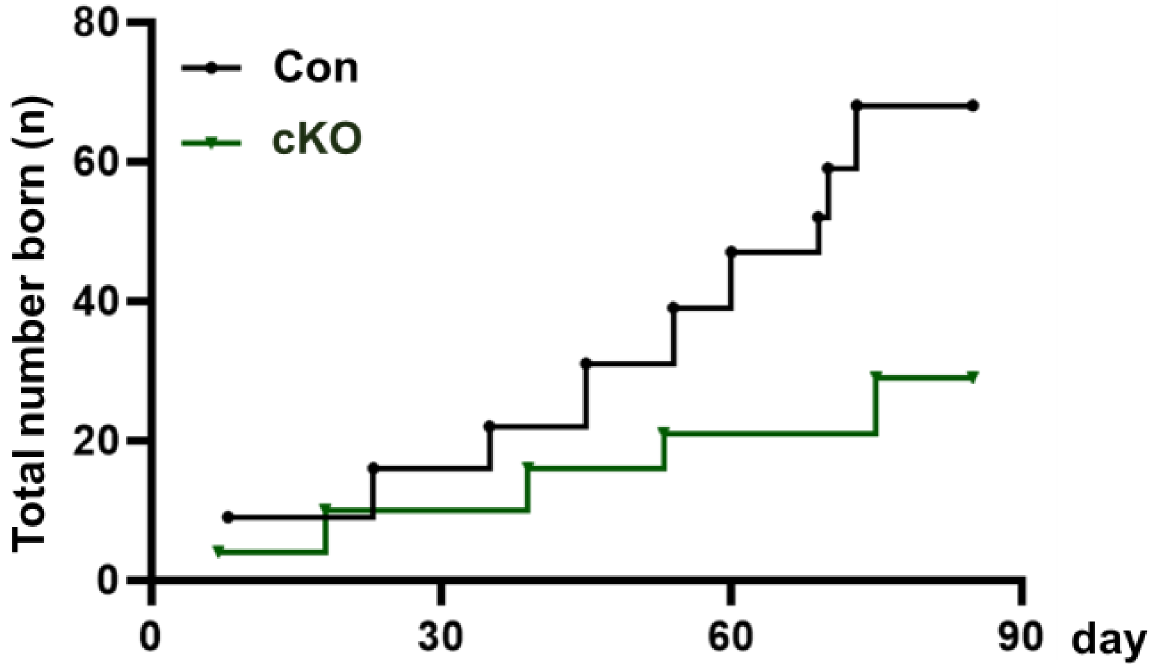

Table Records of birthdate and litter number of mice

	Con		cKO	
	**Birthdate**	**Litter size**	**Birthdate**	**Litter size**
Oct.	8th	9	7th	4
	23rd	7	11th	6
Nov.	6th	6	8th	6
	11th	9	22nd	5
	23rd	8		
Dec.	7th	8	4th	8
	7th	5		
	12th	7		
	8th	9		

Fig.2h,i

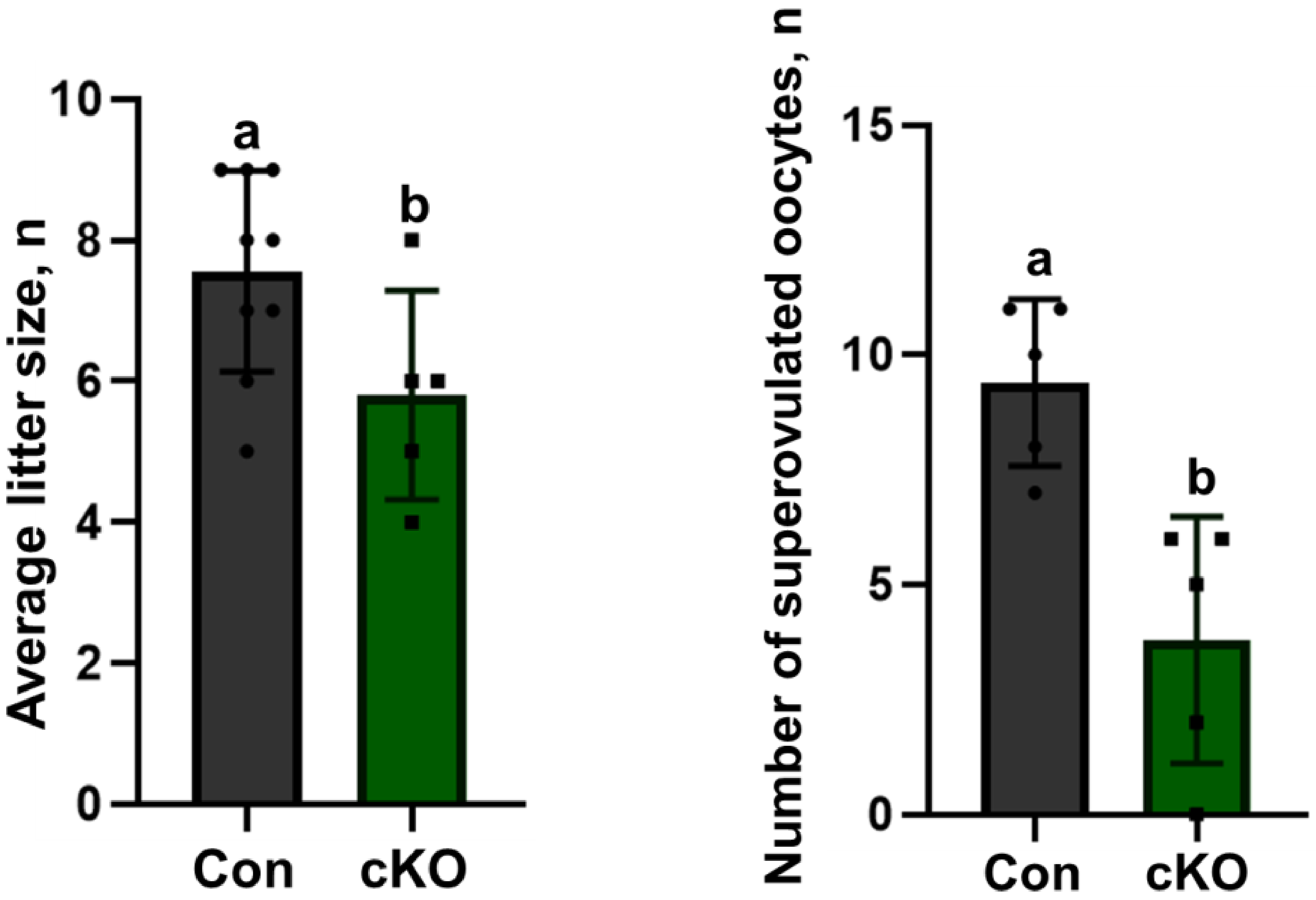

Average litter size, n
10
8
6
4
2
0
a
b
Con
cKO
Number of superovulated oocytes, n
15
10
5
0
a
b
Con
cKO

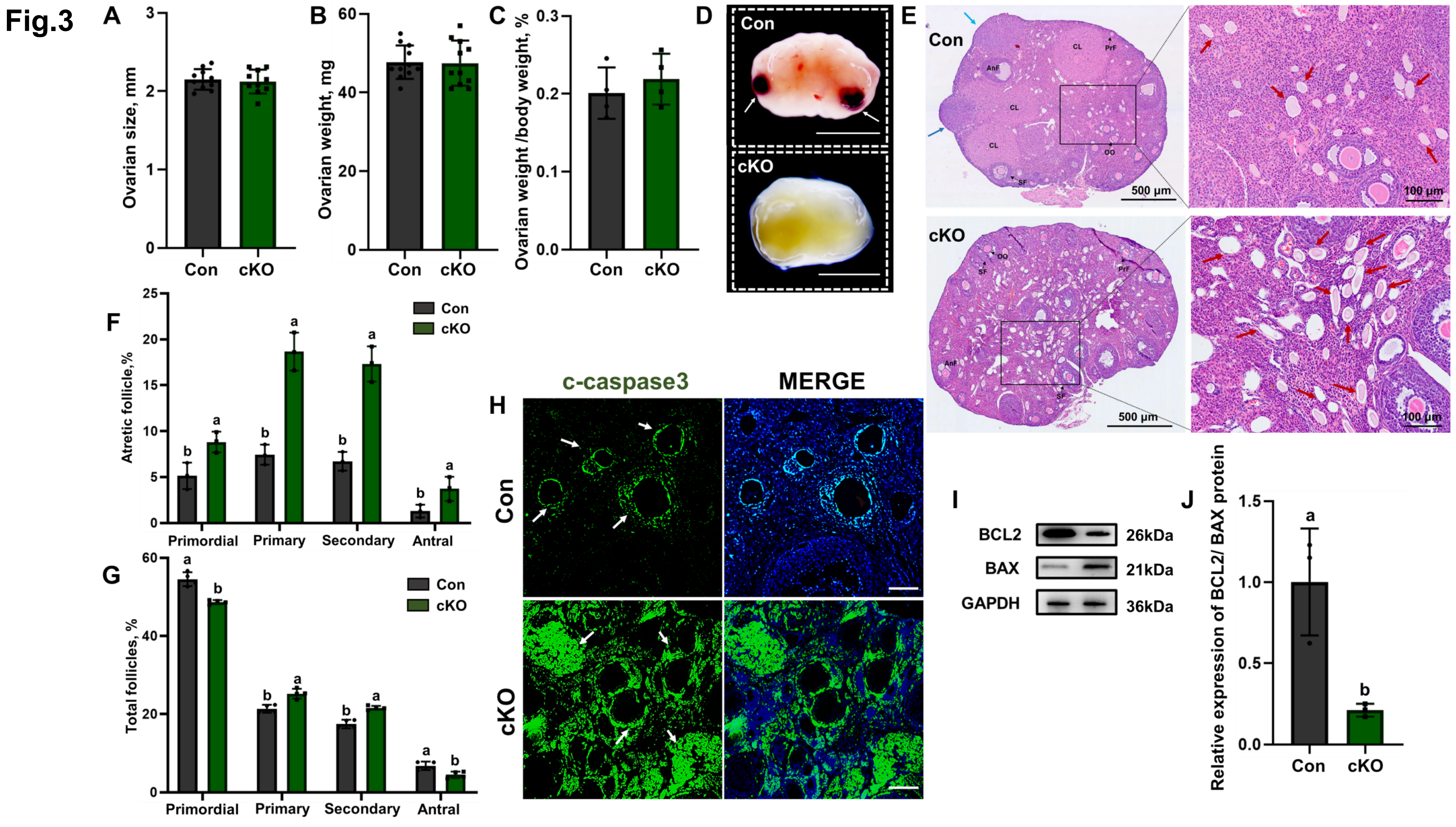
Fig.3
A
Ovarian size, mm
Con
cKO
B
Ovarian weight, mg
C
Ovarian weight /body weight, %
D
Con
cKO
E
Con
cKO
500 μm
100 μm
CL
PrF
AnF
OO
SF
F
Atretic follicle, %
Primordial
Primary
Secondary
Antral
G
Total follicles, %
H
c-caspase3
MERGE
I
BCL2
BAX
GAPDH
26kDa
21kDa
36kDa
J
Relative expression of BCL2/ BAX protein

Fig.3a-3c

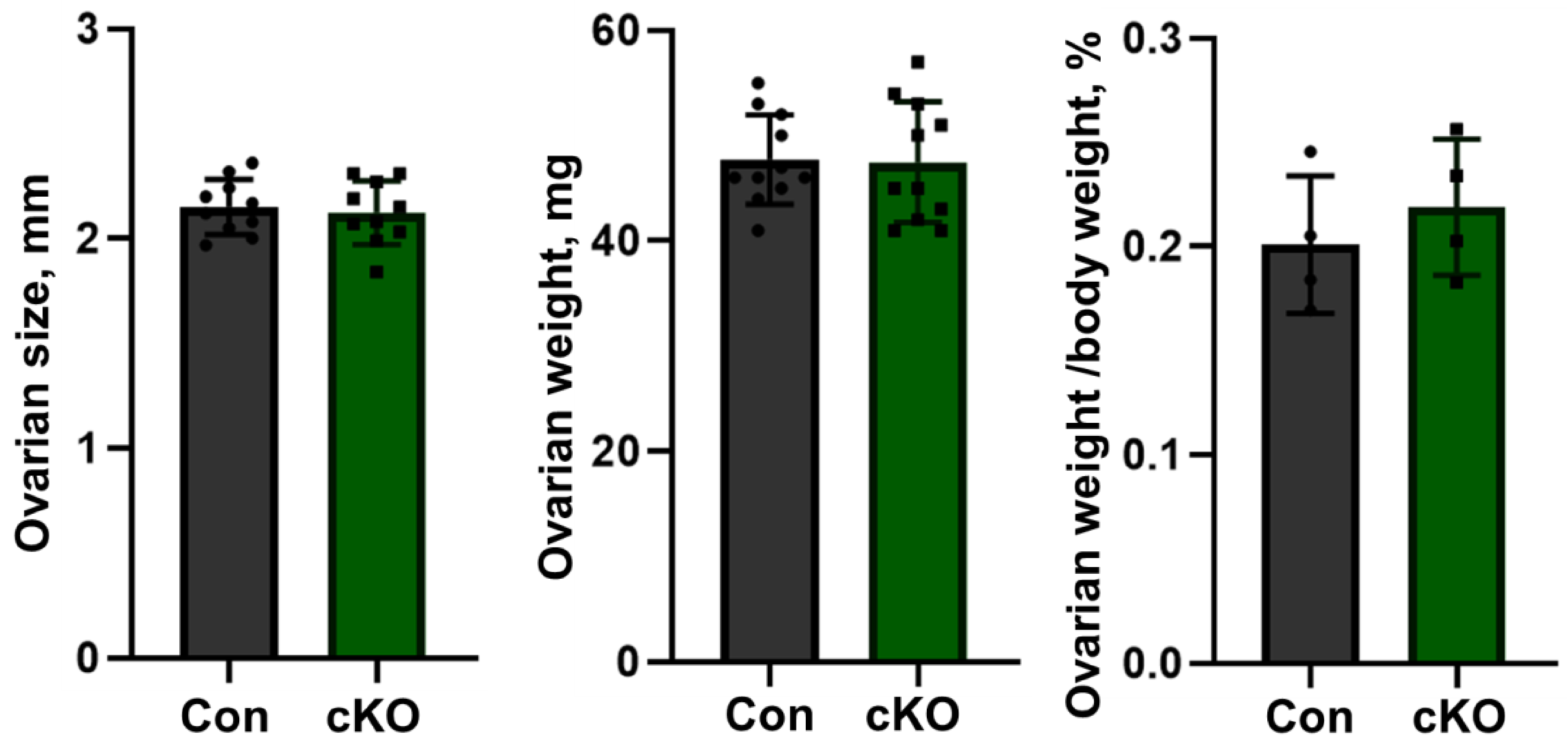

Ovarian size, mm
3
2
1
0
Con
cKO
Ovarian weight, mg
60
40
20
0
Con
cKO
Ovarian weight /body weight, %
0.3
0.2
0.1
0.0
Con
cKO

Fig.3d

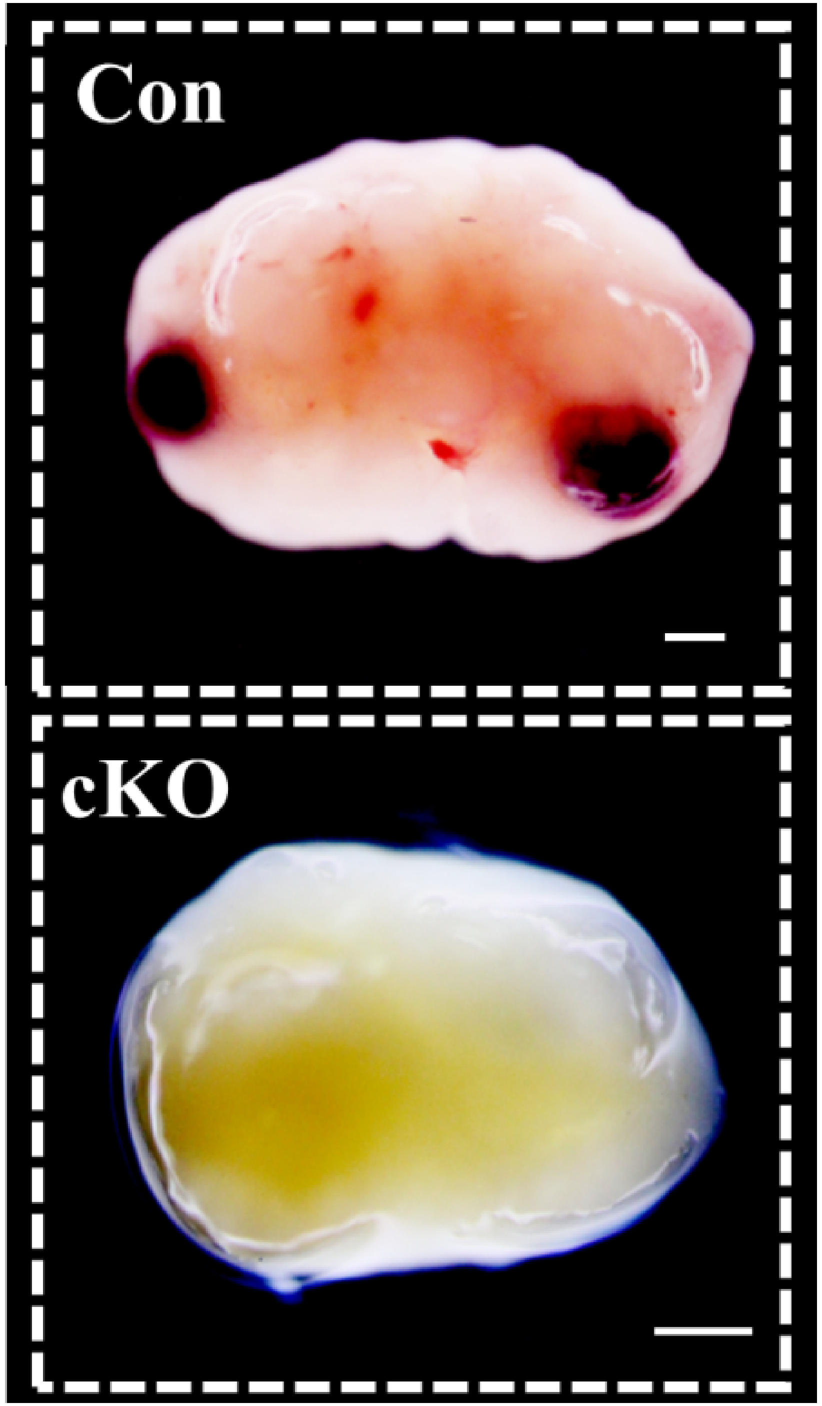

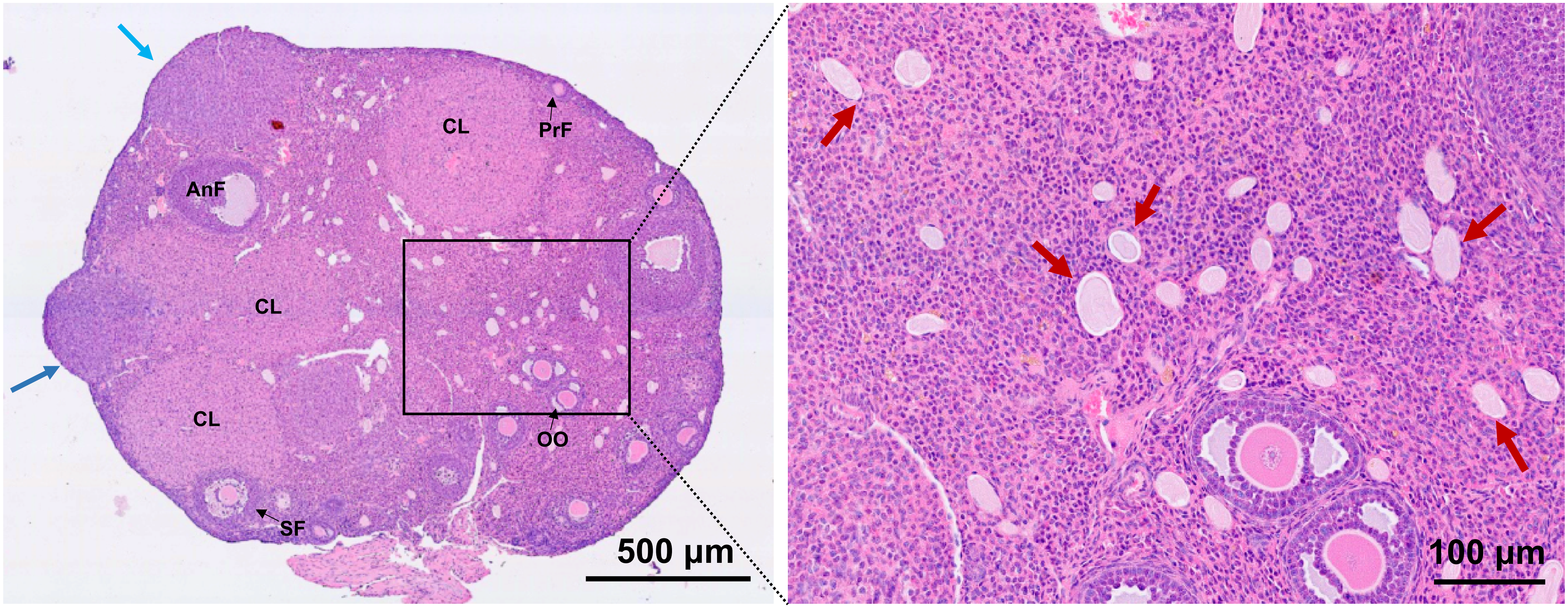
CL
PrF
AnF
CL
CL
OO
SF
500 µm
100 µm

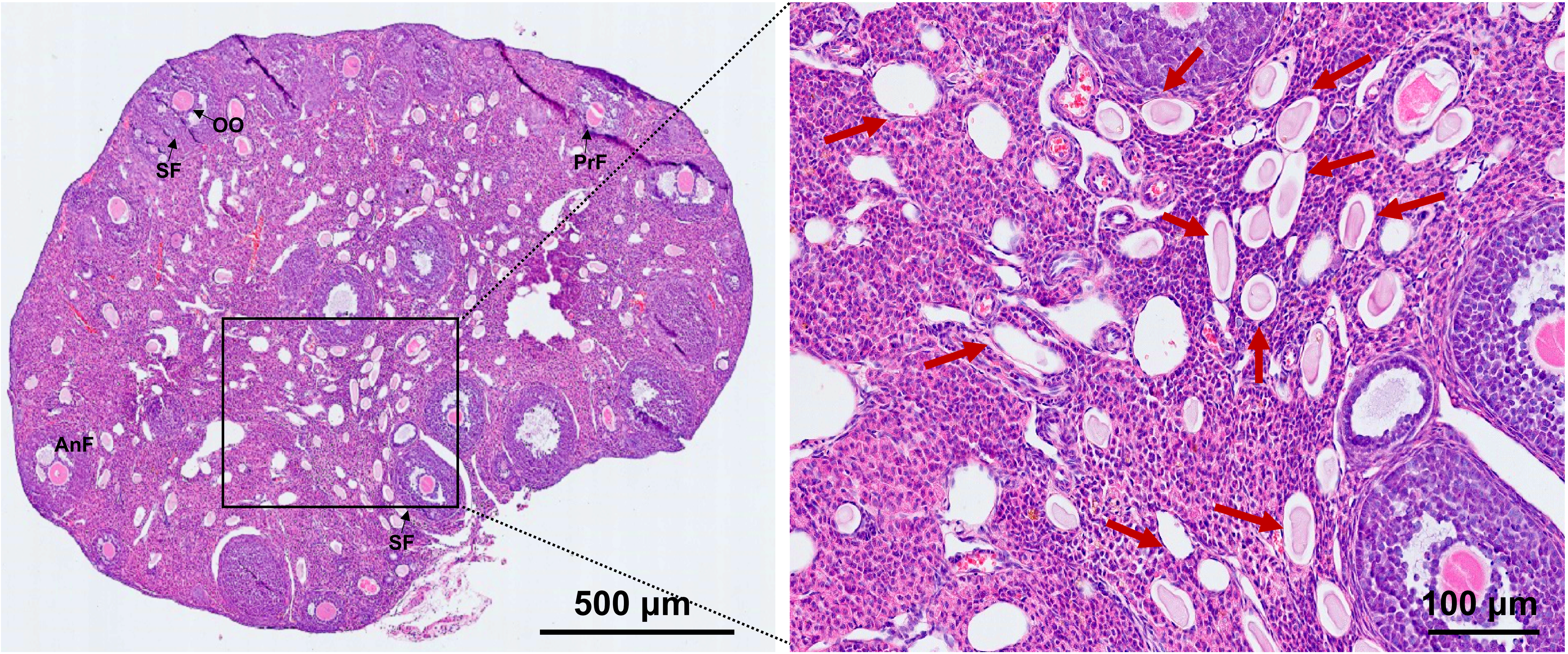
OO
SF
PrF
AnF
SF
500 µm
100 µm

Fig.3f

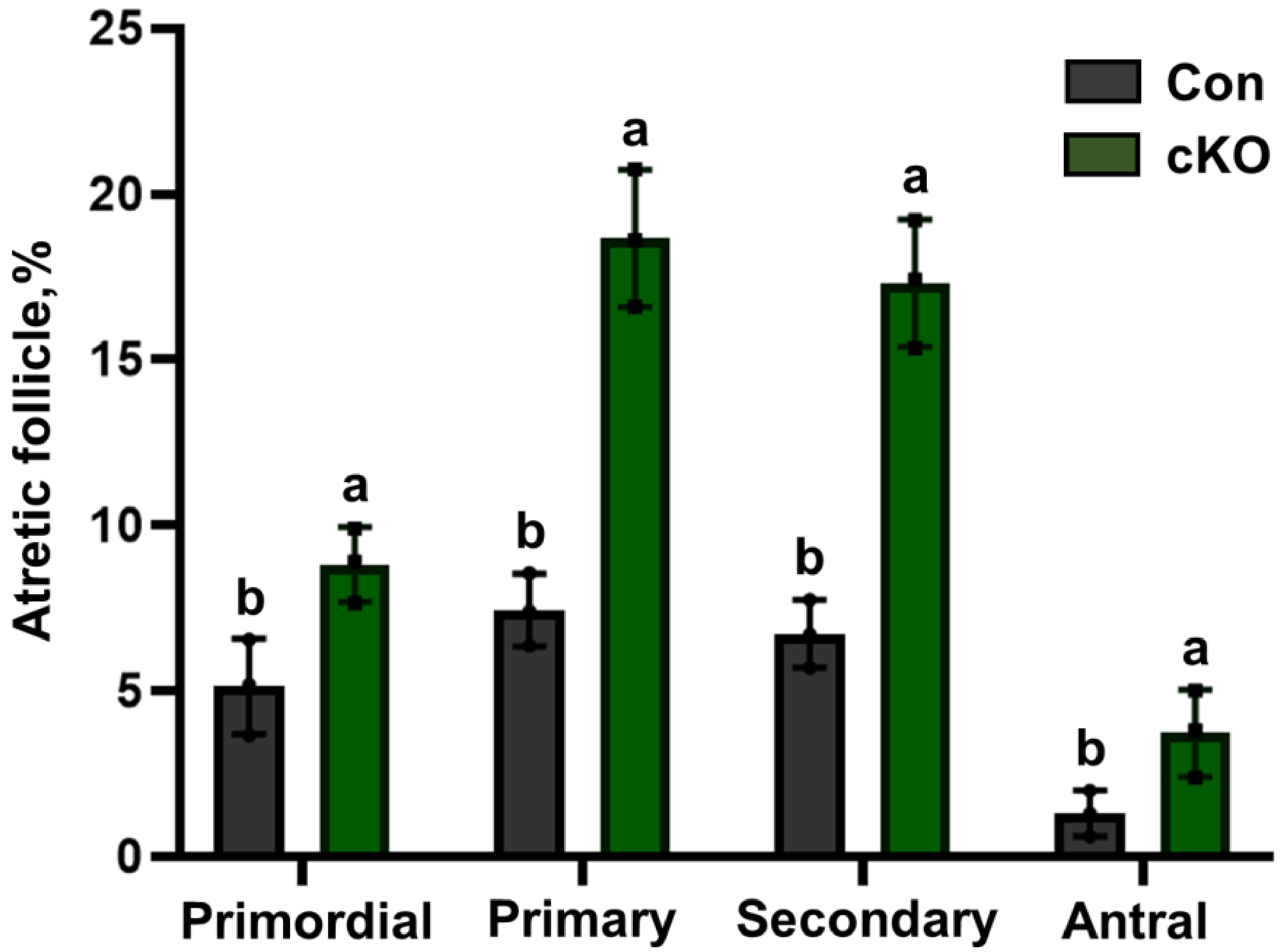

Con
cKO
Atretic follicle,%
25
20
15
10
5
0
a
b
Primordial
Primary
Secondary
Antral

Fig.3h

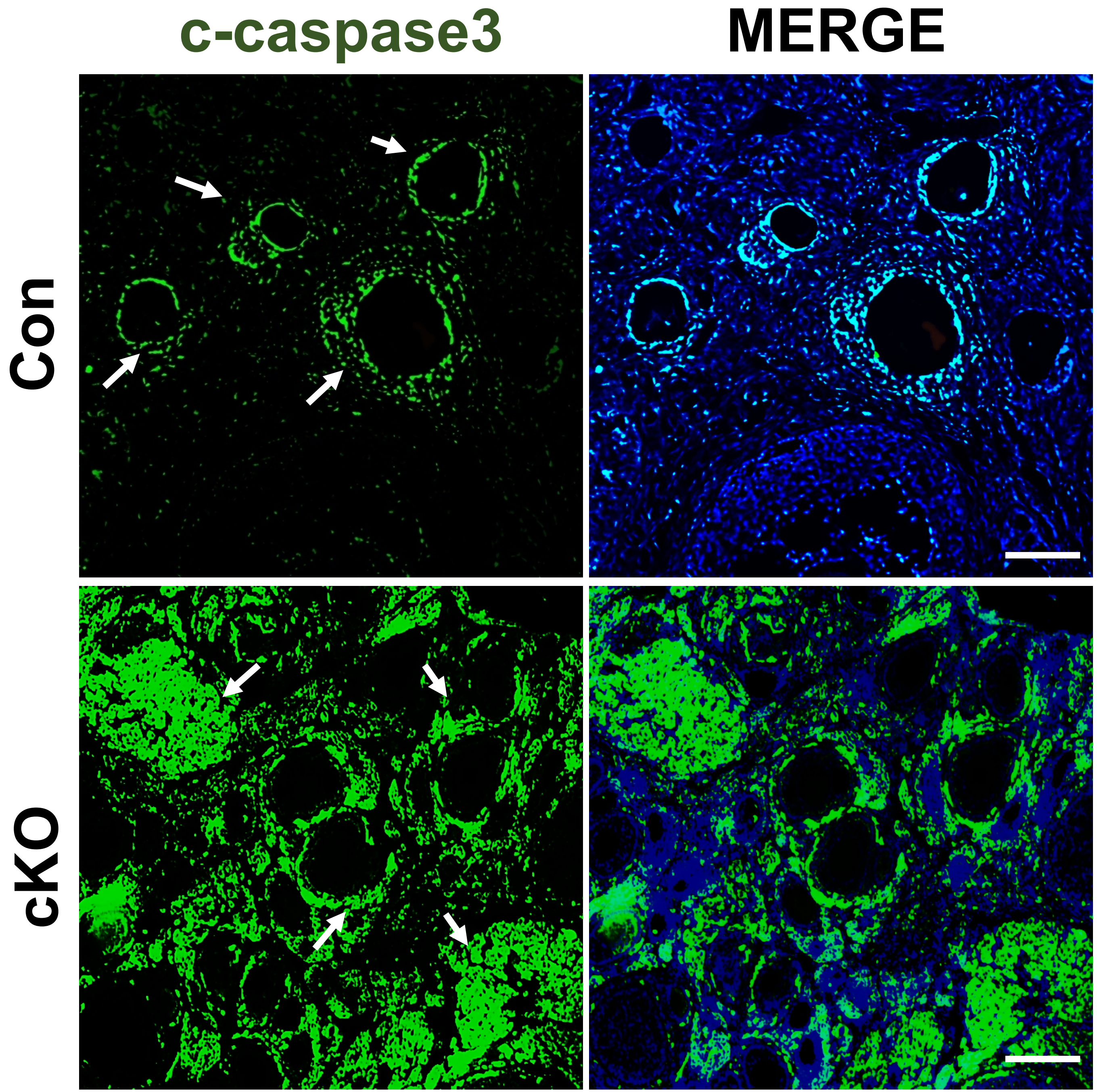

Fig.3i,j

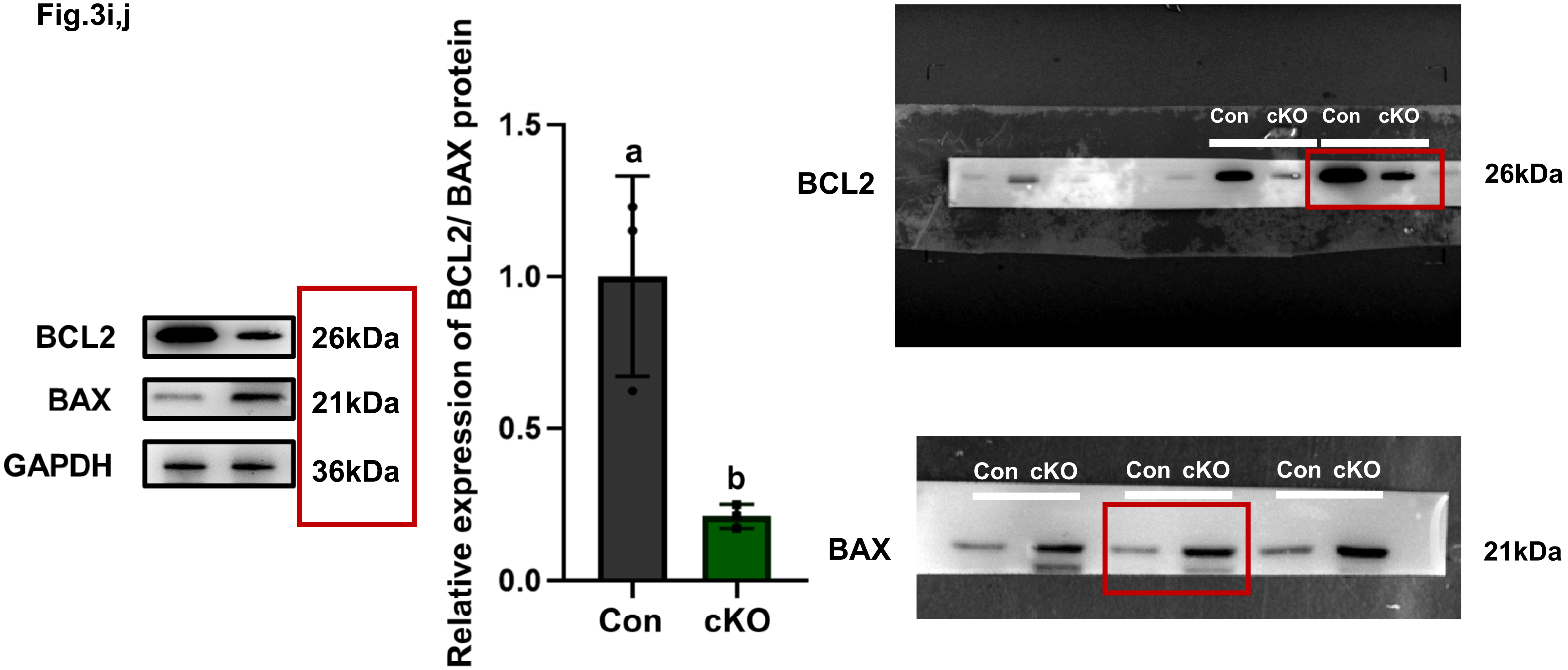

BCL2
BAX
GAPDH
26kDa
21kDa
36kDa
Relative expression of BCL2/ BAX protein
1.5
1.0
0.5
0.0
a
b
Con
cKO
BCL2
Con cKO Con cKO
26kDa
BAX
Con cKO Con cKO Con cKO
21kDa

Fig.4

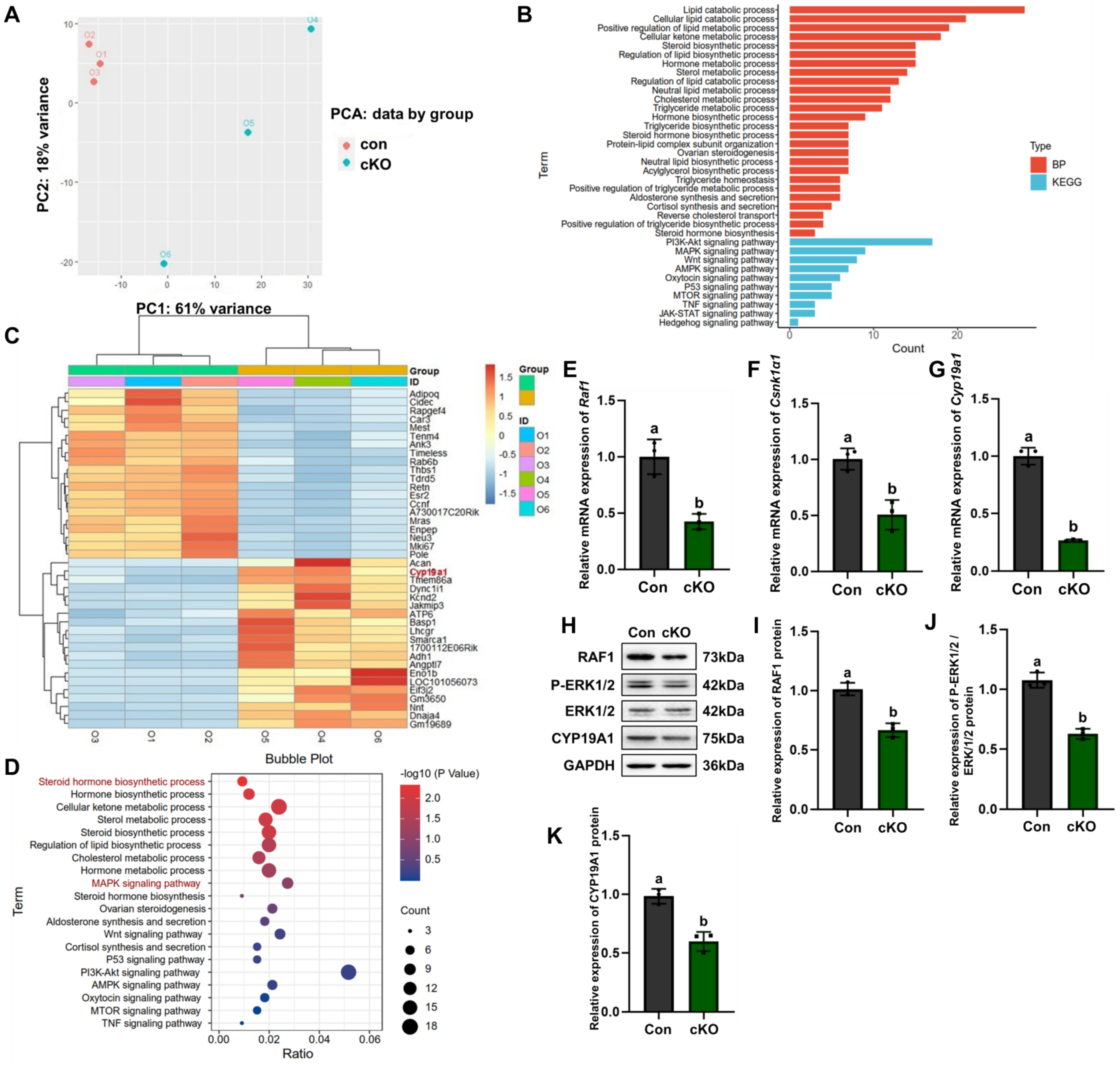
A
PCA: data by group
con
cKO
PC1: 61% variance
PC2: 18% variance
B
Lipid catabolic process
Cellular lipid catabolic process
Positive regulation of lipid metabolic process
Cellular ketone metabolic process
Steroid biosynthetic process
Regulation of lipid biosynthetic process
Hormone metabolic process
Sterol metabolic process
Regulation of lipid catabolic process
Neutral lipid metabolic process
Cholesterol metabolic process
Triglyceride metabolic process
Hormone biosynthetic process
Triglyceride biosynthetic process
Steroid hormone biosynthetic process
Protein-lipid complex subunit organization
Ovarian steroidogenesis
Neutral lipid biosynthetic process
Acylglycerol biosynthetic process
Triglyceride homeostasis
Positive regulation of triglyceride metabolic process
Aldosterone synthesis and secretion
Cortisol synthesis and secretion
Reverse cholesterol transport
Positive regulation of triglyceride biosynthetic process
Steroid hormone biosynthesis
PI3K-Akt signaling pathway
MAPK signaling pathway
Wnt signaling pathway
AMPK signaling pathway
Oxytocin signaling pathway
P53 signaling pathway
MTOR signaling pathway
TNF signaling pathway
JAK-STAT signaling pathway
Hedgehog signaling pathway
Term
Count
Type
BP
KEGG
C
Group
ID
Cyp19a1
D
Bubble Plot
Steroid hormone biosynthetic process
MAPK signaling pathway
-log10 (P Value)
Count
Ratio
Term
E
Relative mRNA expression of Raf1
F
Relative mRNA expression of Csnk1a1
G
Relative mRNA expression of Cyp19a1
Con
cKO
H
RAF1 73kDa
P-ERK1/2 42kDa
ERK1/2 42kDa
CYP19A1 75kDa
GAPDH 36kDa
I
Relative expression of RAF1 protein
J
Relative expression of P-ERK1/2 / ERK1/2 protein
K
Relative expression of CYP19A1 protein

Fig.4a

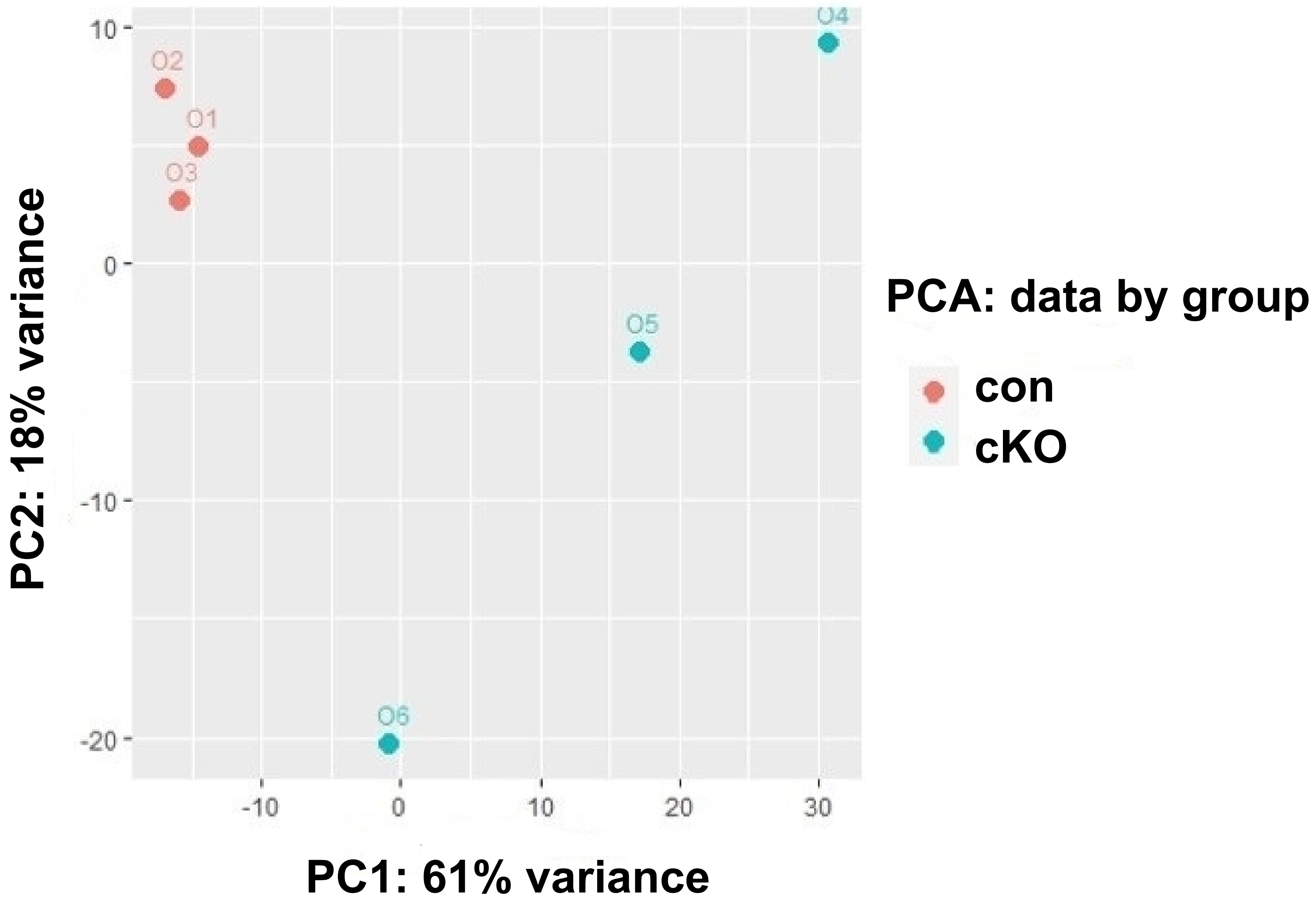

10
0
-10
-20
-10
0
10
20
30
O1
O2
O3
O4
O5
O6
PC2: 18% variance
PC1: 61% variance
PCA: data by group
con
cKO

Fig.4b

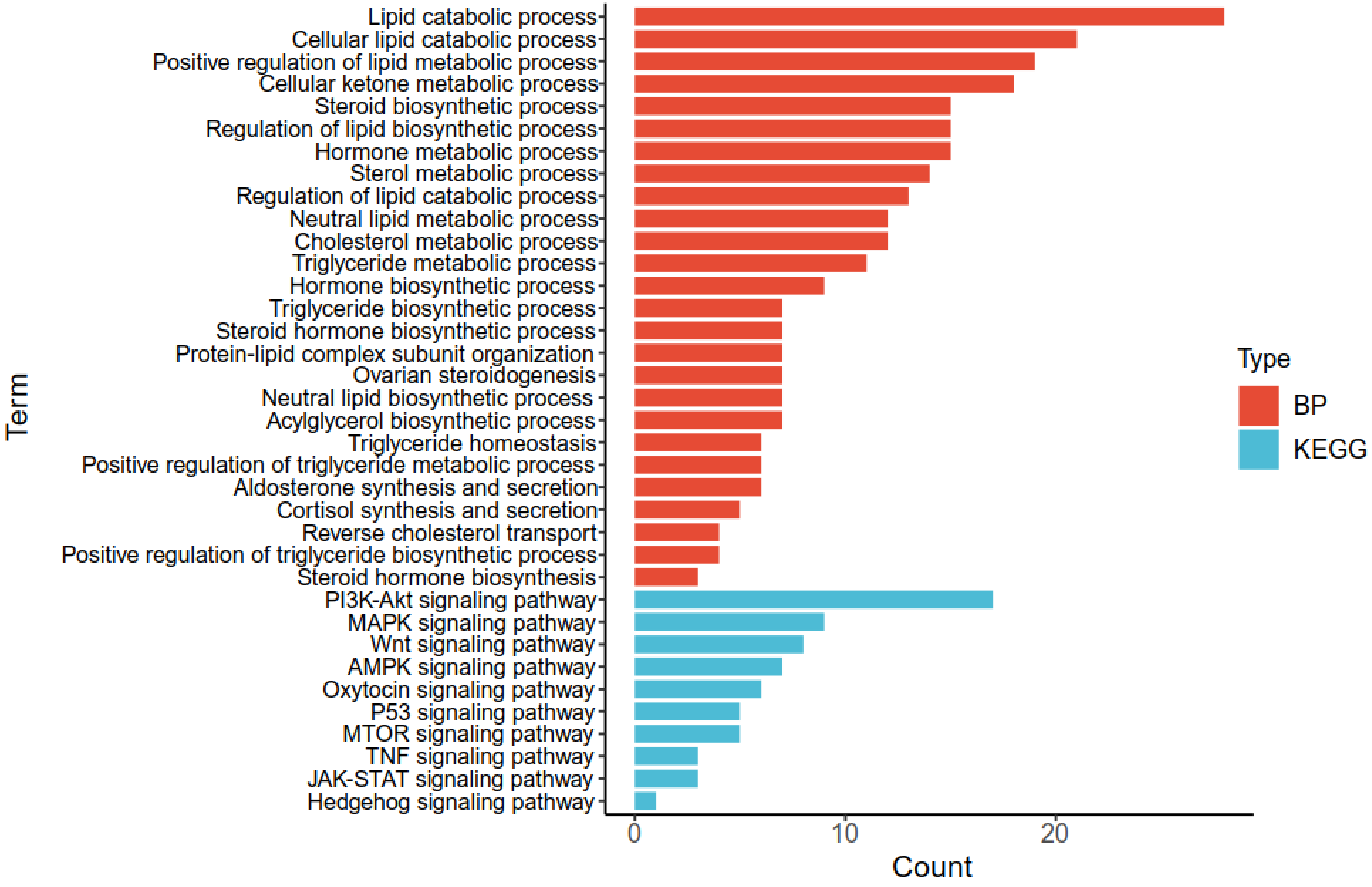
Lipid catabolic process
Cellular lipid catabolic process
Positive regulation of lipid metabolic process
Cellular ketone metabolic process
Steroid biosynthetic process
Regulation of lipid biosynthetic process
Hormone metabolic process
Sterol metabolic process
Regulation of lipid catabolic process
Neutral lipid metabolic process
Cholesterol metabolic process
Triglyceride metabolic process
Hormone biosynthetic process
Triglyceride biosynthetic process
Steroid hormone biosynthetic process
Protein-lipid complex subunit organization
Ovarian steroidogenesis
Neutral lipid biosynthetic process
Acylglycerol biosynthetic process
Triglyceride homeostasis
Positive regulation of triglyceride metabolic process
Aldosterone synthesis and secretion
Cortisol synthesis and secretion
Reverse cholesterol transport
Positive regulation of triglyceride biosynthetic process
Steroid hormone biosynthesis
PI3K-Akt signaling pathway
MAPK signaling pathway
Wnt signaling pathway
AMPK signaling pathway
Oxytocin signaling pathway
P53 signaling pathway
MTOR signaling pathway
TNF signaling pathway
JAK-STAT signaling pathway
Hedgehog signaling pathway
Term
0
10
20
Count
Type
BP
KEGG

Fig.4c

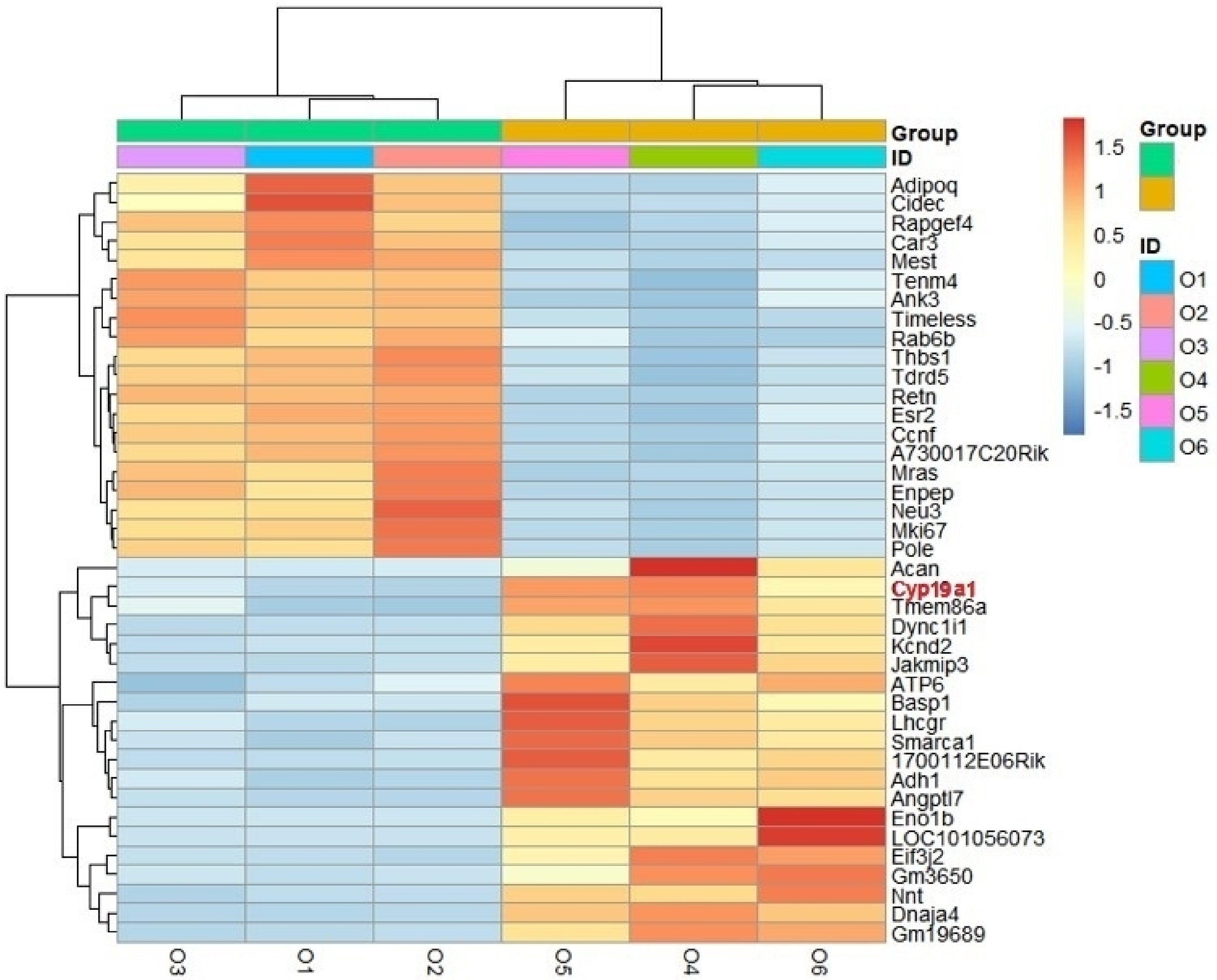
Group
ID
Adipoq
Cidec
Rapgef4
Car3
Mest
Tenm4
Ank3
Timeless
Rab6b
Thbs1
Tdrd5
Retn
Esr2
Ccnf
A730017C20Rik
Mras
Enpep
Neu3
Mki67
Pole
Acan
Cyp19a1
Tmem86a
Dync1i1
Kcnd2
Jakmip3
ATP6
Basp1
Lhcgr
Smarca1
1700112E06Rik
Adh1
Angptl7
Eno1b
LOC101056073
Eif3j2
Gm3650
Nnt
Dnaja4
Gm19689
O3
O1
O2
O5
O4
O6
1.5
1
0.5
0
-0.5
-1
-1.5
Group
ID
O1
O2
O3
O4
O5
O6

Fig.4d

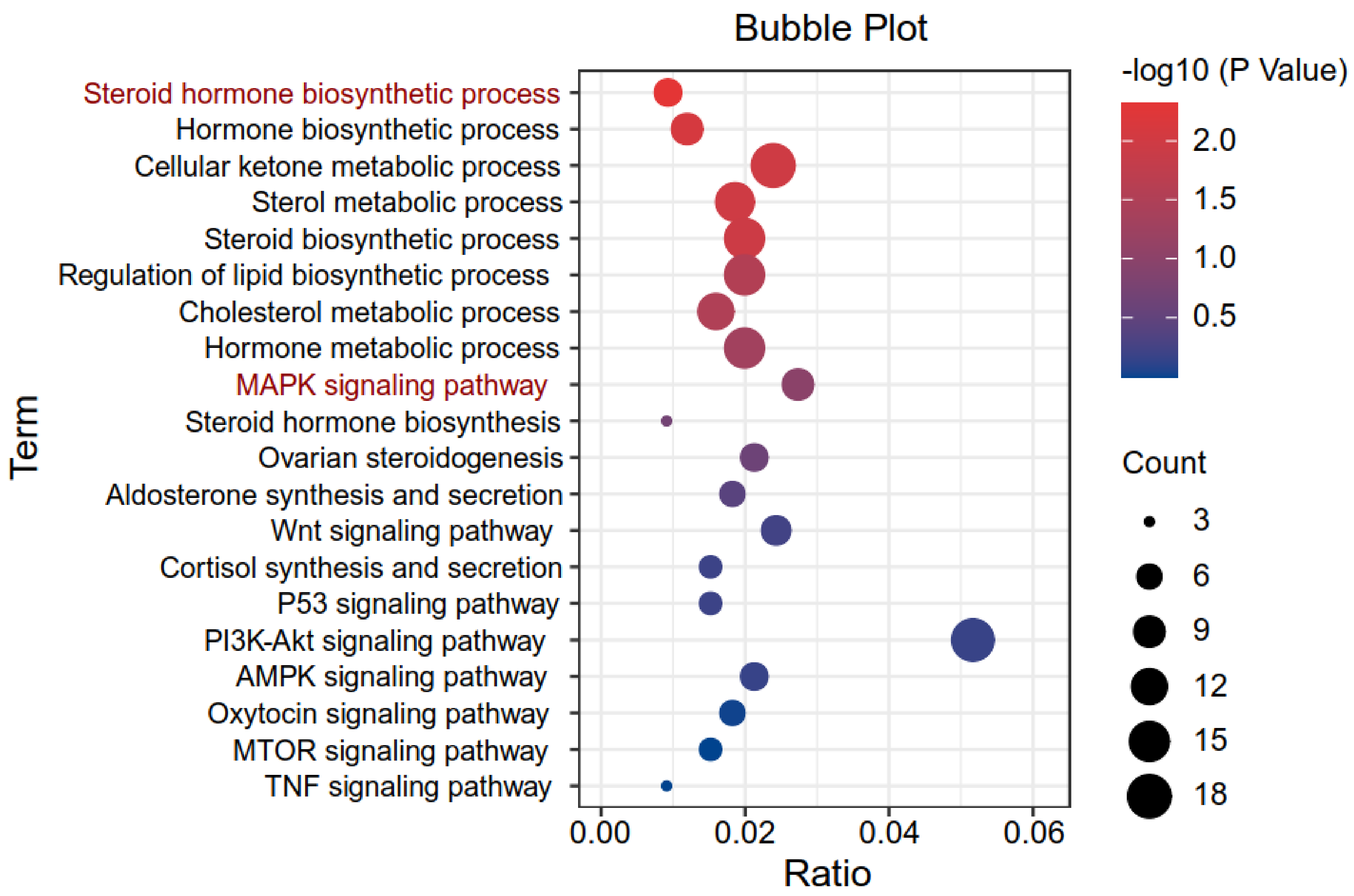

Bubble Plot
-log10 (P Value)
2.0
1.5
1.0
0.5
Count
3
6
9
12
15
18
Term
Steroid hormone biosynthetic process
Hormone biosynthetic process
Cellular ketone metabolic process
Sterol metabolic process
Steroid biosynthetic process
Regulation of lipid biosynthetic process
Cholesterol metabolic process
Hormone metabolic process
MAPK signaling pathway
Steroid hormone biosynthesis
Ovarian steroidogenesis
Aldosterone synthesis and secretion
Wnt signaling pathway
Cortisol synthesis and secretion
P53 signaling pathway
PI3K-Akt signaling pathway
AMPK signaling pathway
Oxytocin signaling pathway
MTOR signaling pathway
TNF signaling pathway
0.00
0.02
0.04
0.06
Ratio

Fig.4e-4g

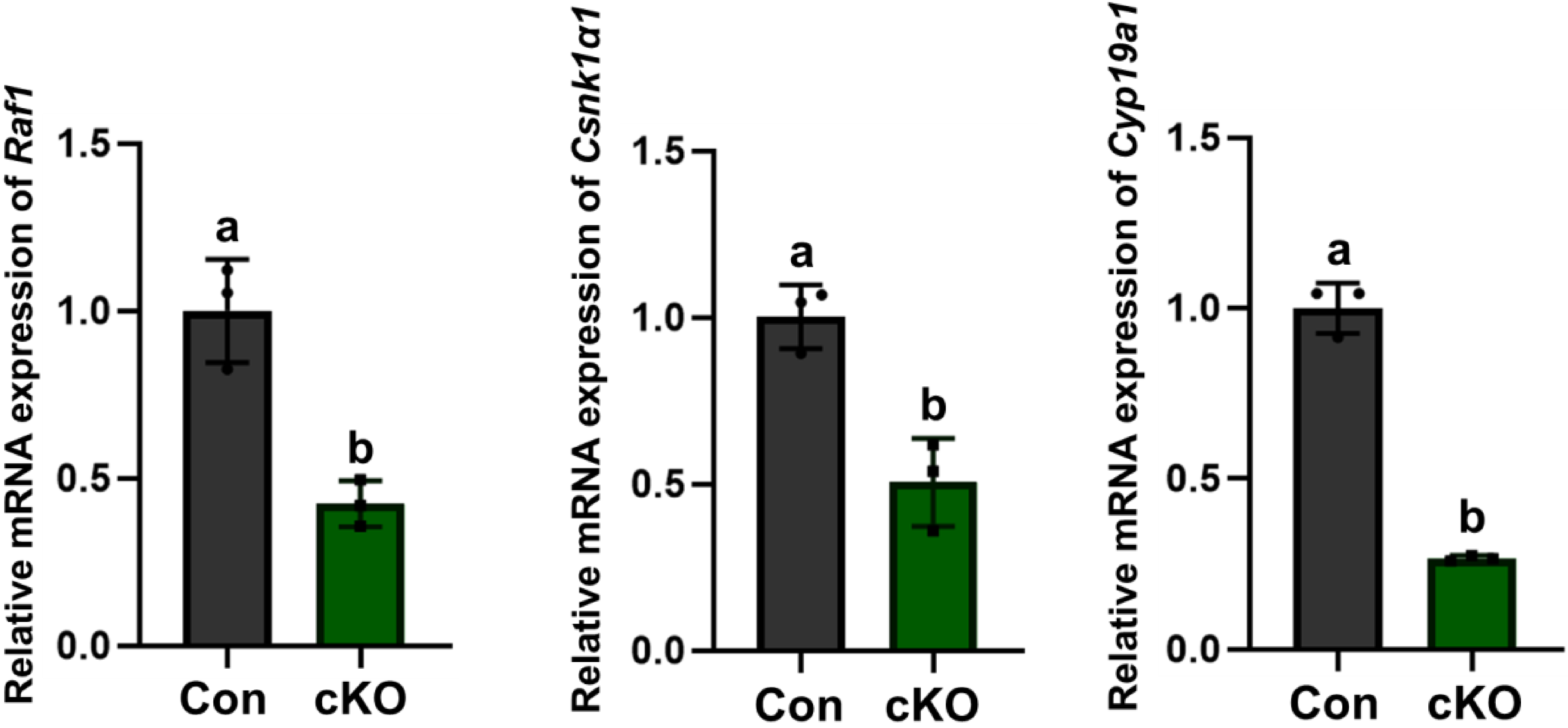

Relative mRNA expression of *Raf1*
1.5
1.0
0.5
0.0
a
b
Con
cKO
Relative mRNA expression of *Csnk1α1*
1.5
1.0
0.5
0.0
a
b
Con
cKO
Relative mRNA expression of *Cyp19a1*
1.5
1.0
0.5
0.0
a
b
Con
cKO

Fig.4h

Organizational samples

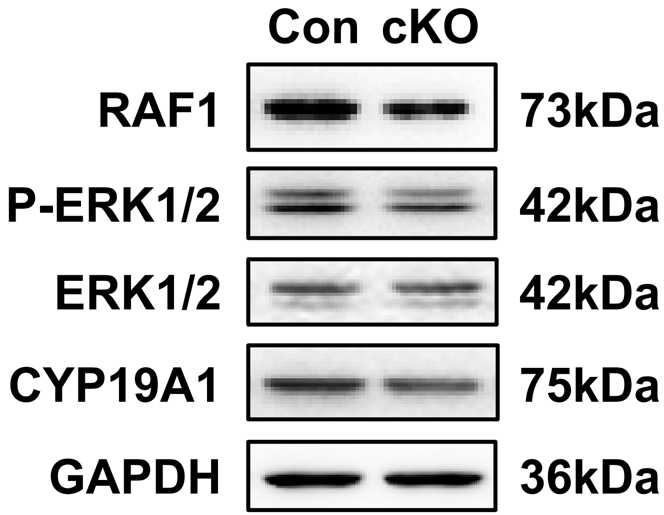

Fig.4h

Organizational samples

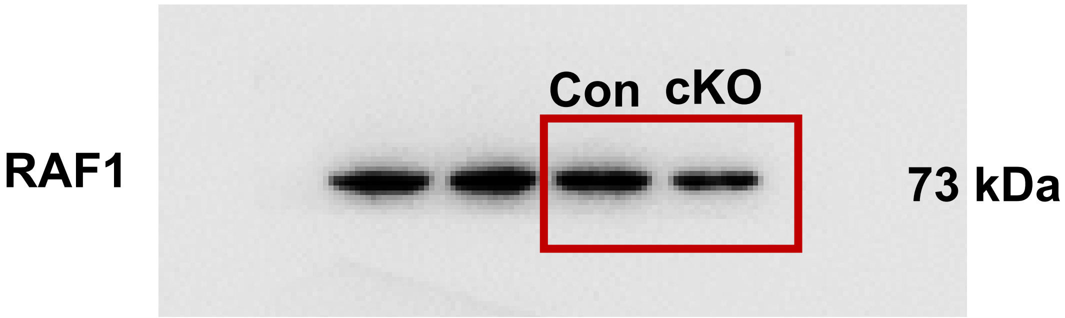

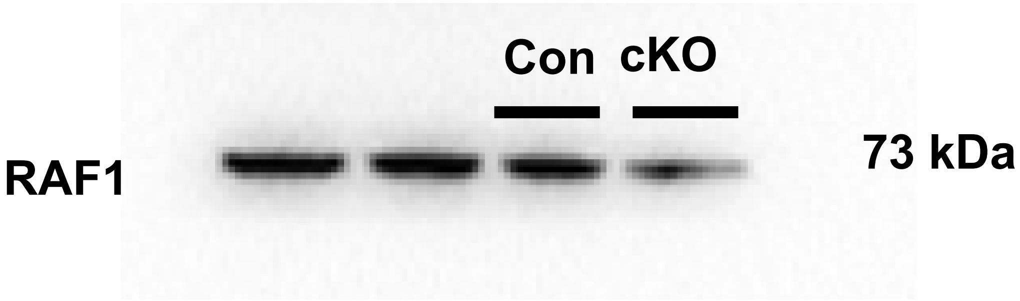

Fig.4h

Organizational samples

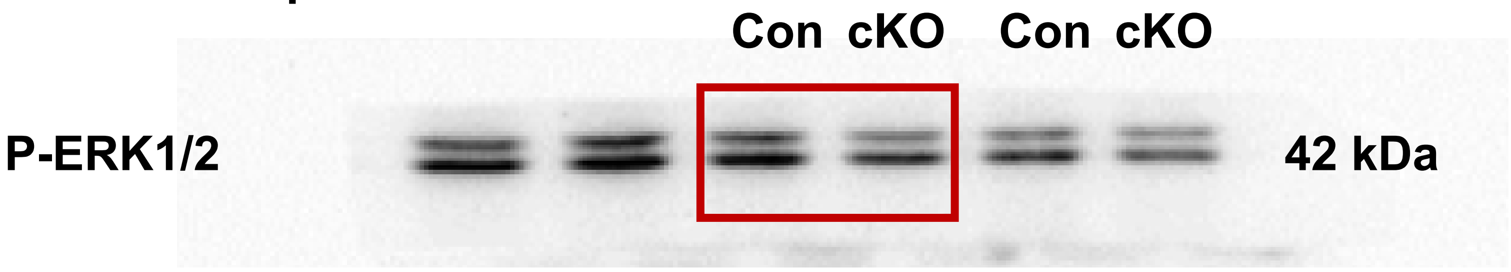

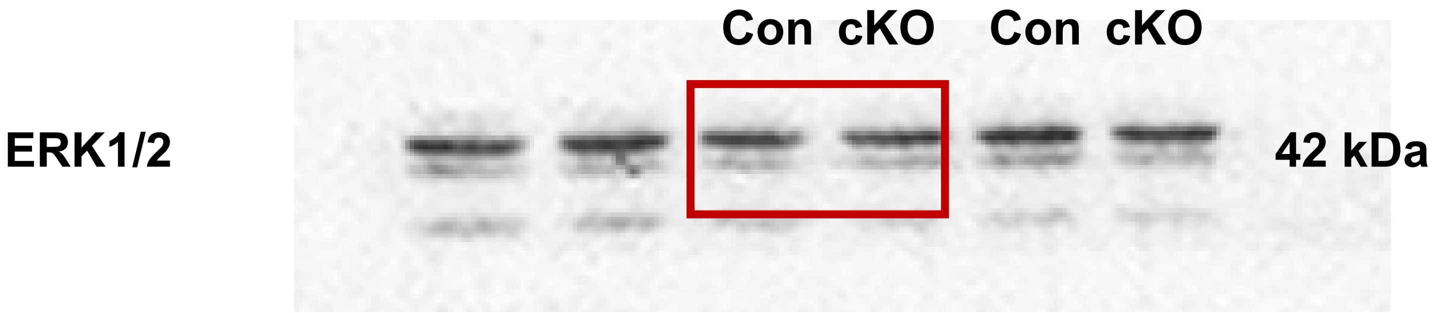

Fig.4h

Organizational samples

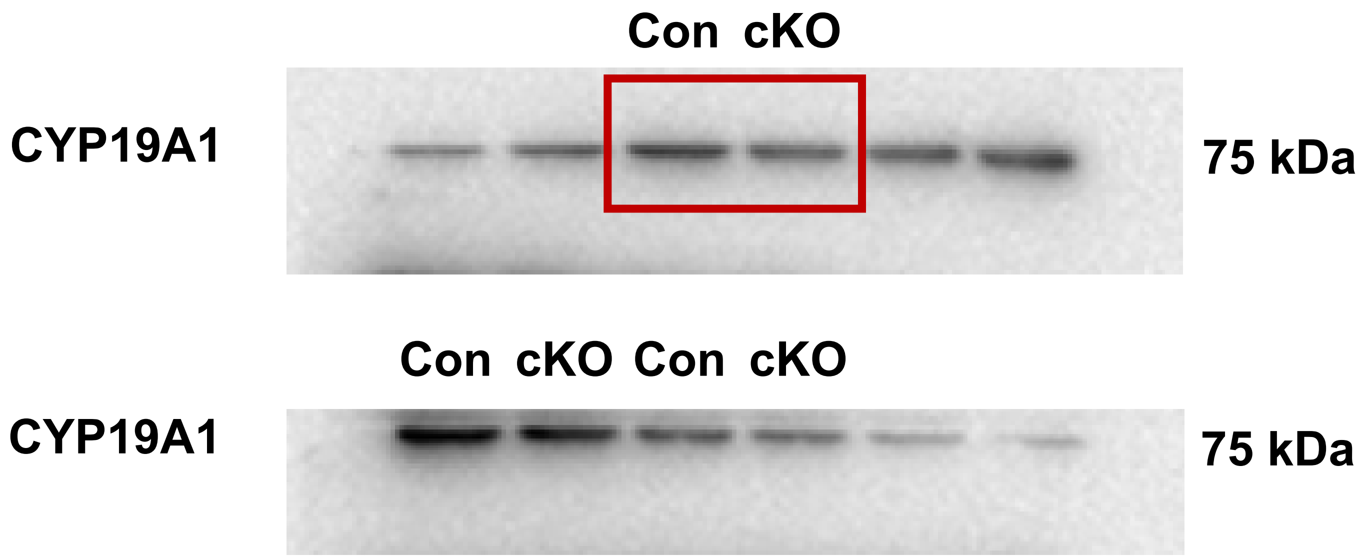

Fig.4h

Organizational samples

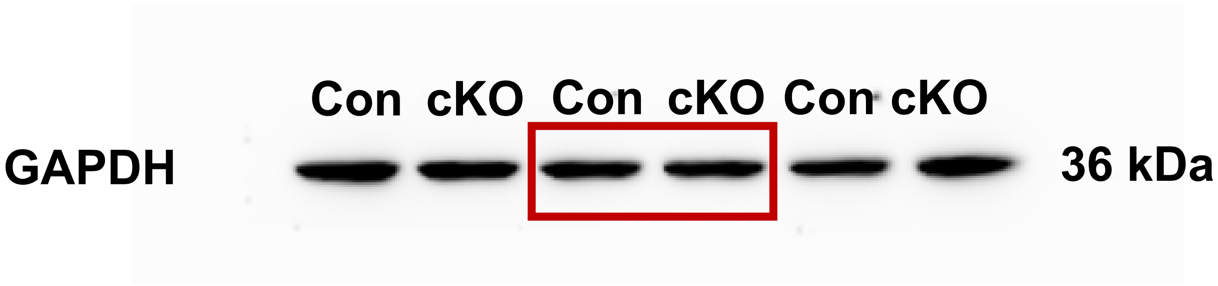

Fig.4i-4k

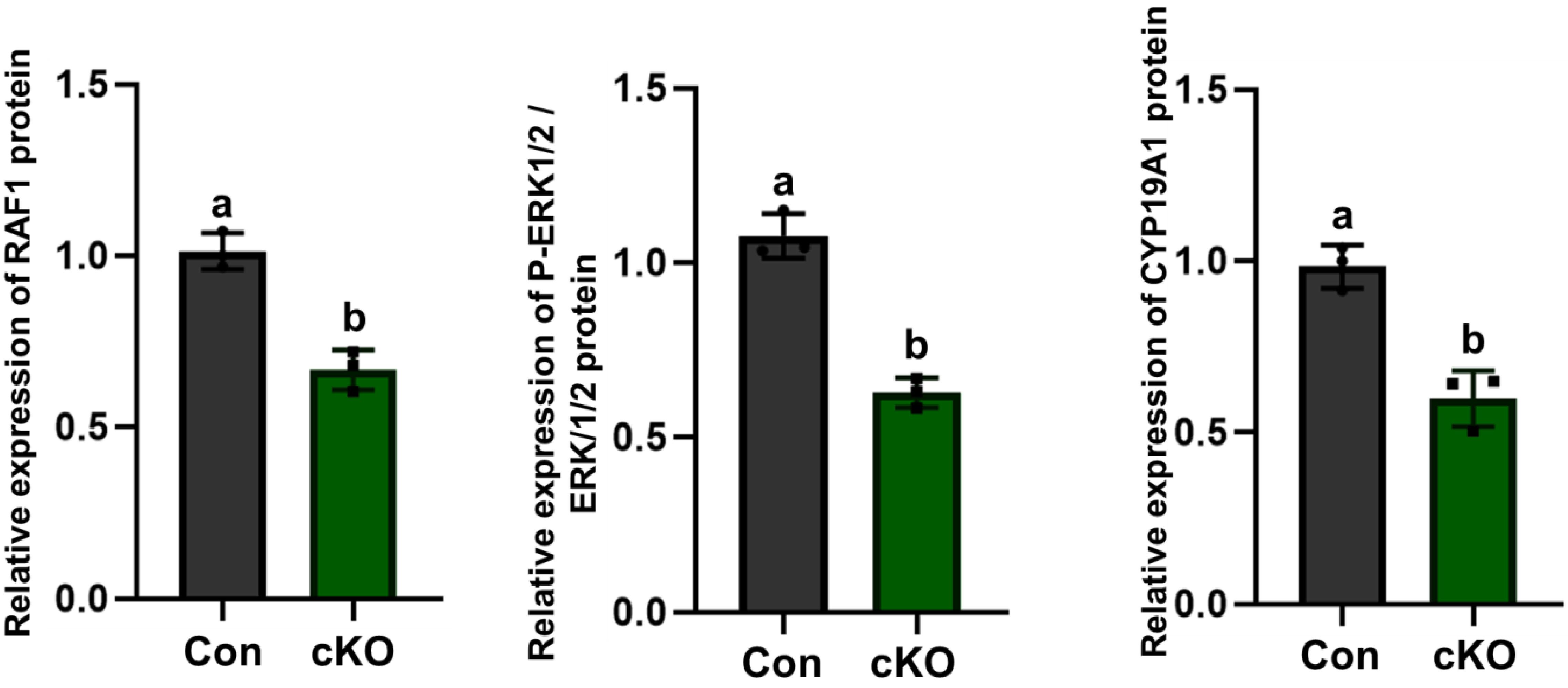
Relative expression of RAF1 protein
1.5
1.0
0.5
0.0
a
b
Con
cKO
Relative expression of P-ERK1/2 / ERK/1/2 protein
1.5
1.0
0.5
0.0
a
b
Con
cKO
Relative expression of CYP19A1 protein
1.5
1.0
0.5
0.0
a
b
Con
cKO

Fig.5

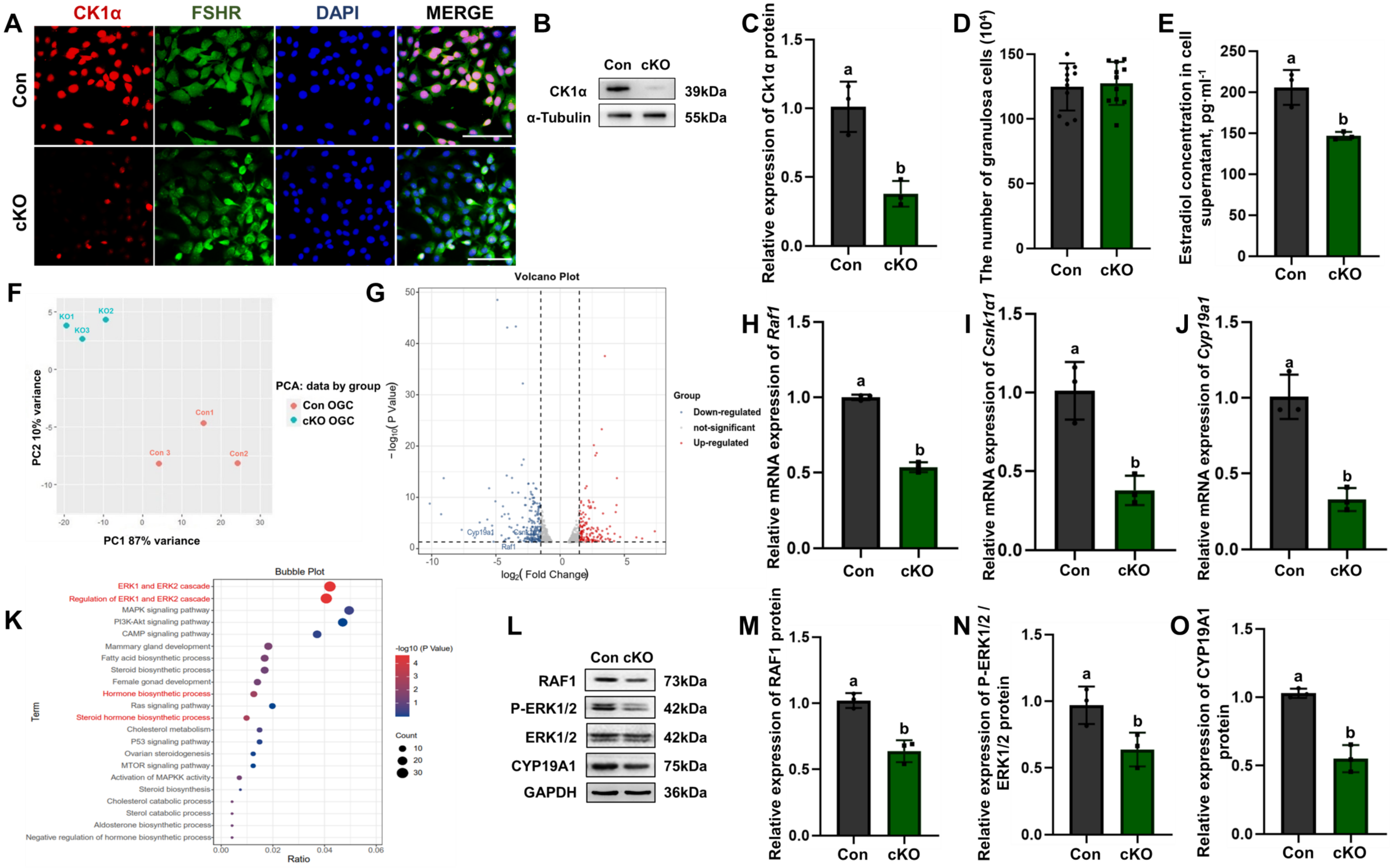
A
CK1α
FSHR
DAPI
MERGE
Con
cKO
B
Con cKO
CK1α
α-Tubulin
39kDa
55kDa
C
Relative expression of Ck1α protein
D
The number of granulosa cells (10⁴)
E
Estradiol concentration in cell supernatant, pg·ml⁻¹
F
PCA: data by group
Con OGC
cKO OGC
PC1 87% variance
PC2 10% variance
G
Volcano Plot
Group
Down-regulated
not-significant
Up-regulated
Cyp19a1
Raf1
H
Relative mRNA expression of Raf1
I
Relative mRNA expression of Csnk1α1
J
Relative mRNA expression of Cyp19a1
K
Bubble Plot
ERK1 and ERK2 cascade
Regulation of ERK1 and ERK2 cascade
MAPK signaling pathway
PI3K-Akt signaling pathway
CAMP signaling pathway
Mammary gland development
Fatty acid biosynthetic process
Steroid biosynthetic process
Female gonad development
Hormone biosynthetic process
Ras signaling pathway
Steroid hormone biosynthetic process
Cholesterol metabolism
P53 signaling pathway
Ovarian steroidogenesis
MTOR signaling pathway
Activation of MAPKK activity
Steroid biosynthesis
Cholesterol catabolic process
Sterol catabolic process
Aldosterone biosynthetic process
Negative regulation of hormone biosynthetic process
Ratio
Term
-log10 (P Value)
Count
L
Con cKO
RAF1
P-ERK1/2
ERK1/2
CYP19A1
GAPDH
73kDa
42kDa
42kDa
75kDa
36kDa
M
Relative expression of RAF1 protein
N
Relative expression of P-ERK1/2 / ERK1/2 protein
O
Relative expression of CYP19A1 protein

Fig.5a

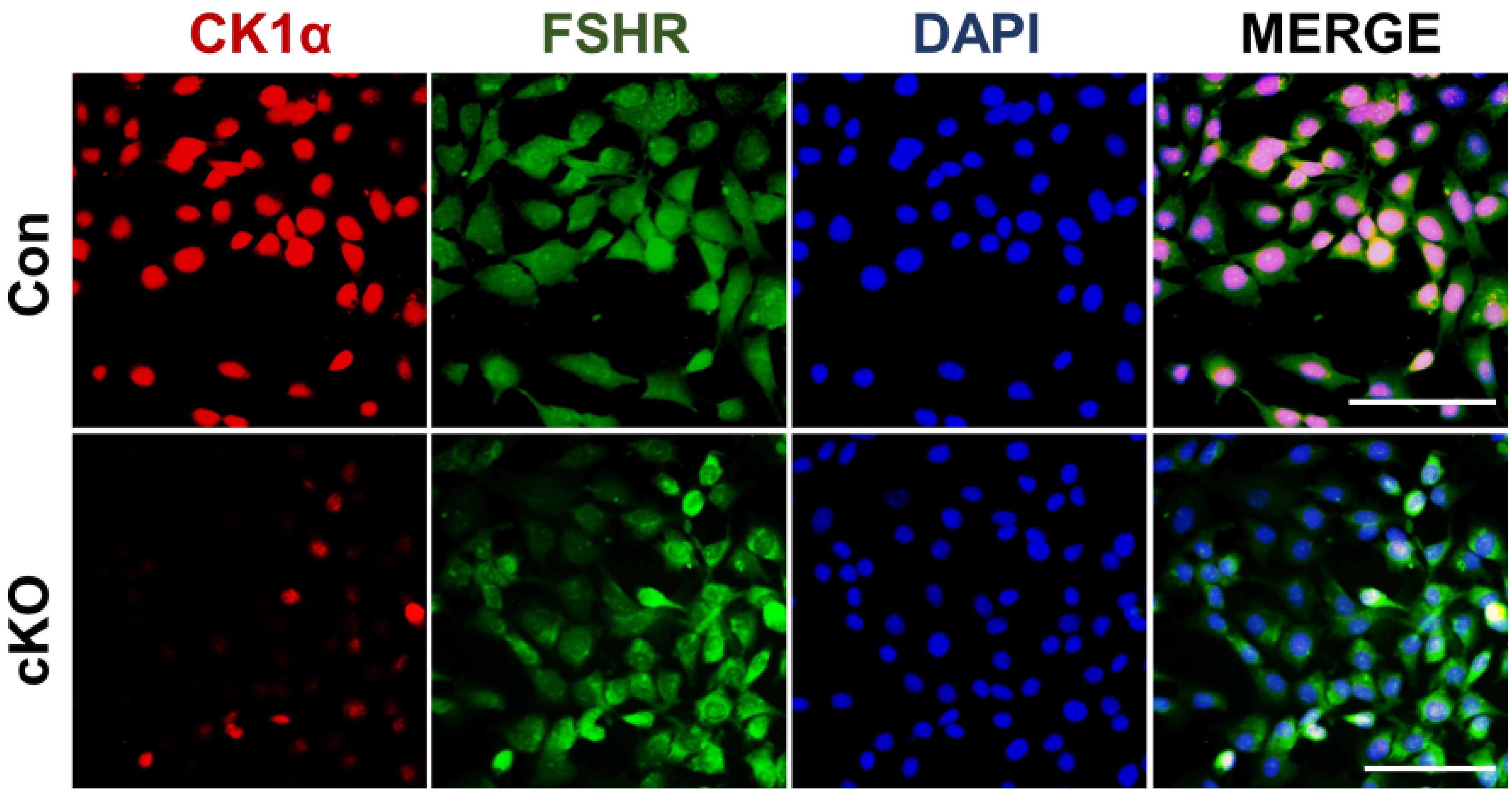

Fig.5b,c

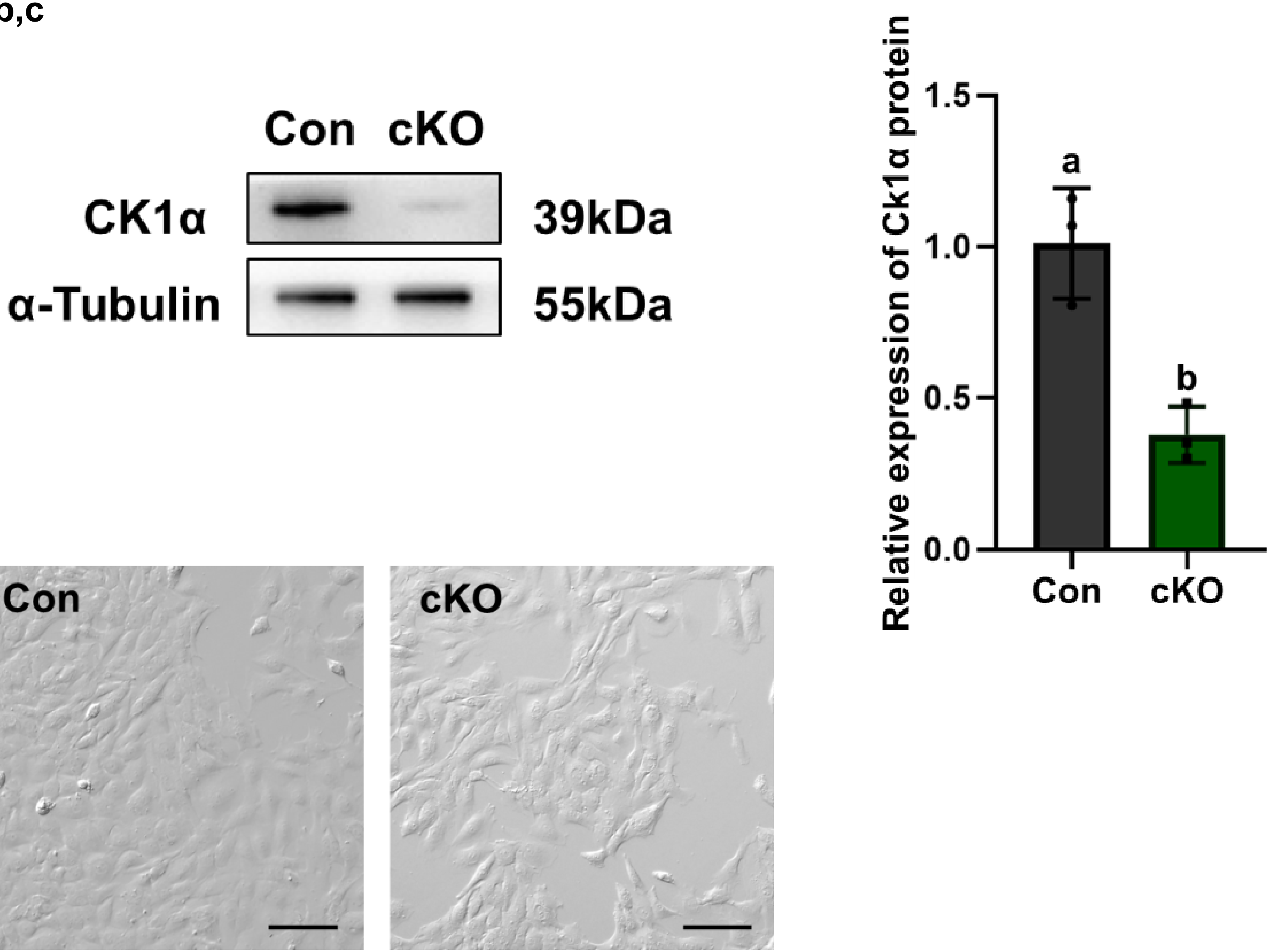
Con
cKO
CK1α
39kDa
α-Tubulin
55kDa
Relative expression of Ck1α protein
1.5
1.0
0.5
0.0
a
b
Con
cKO
Con
cKO

Fig.5b,c

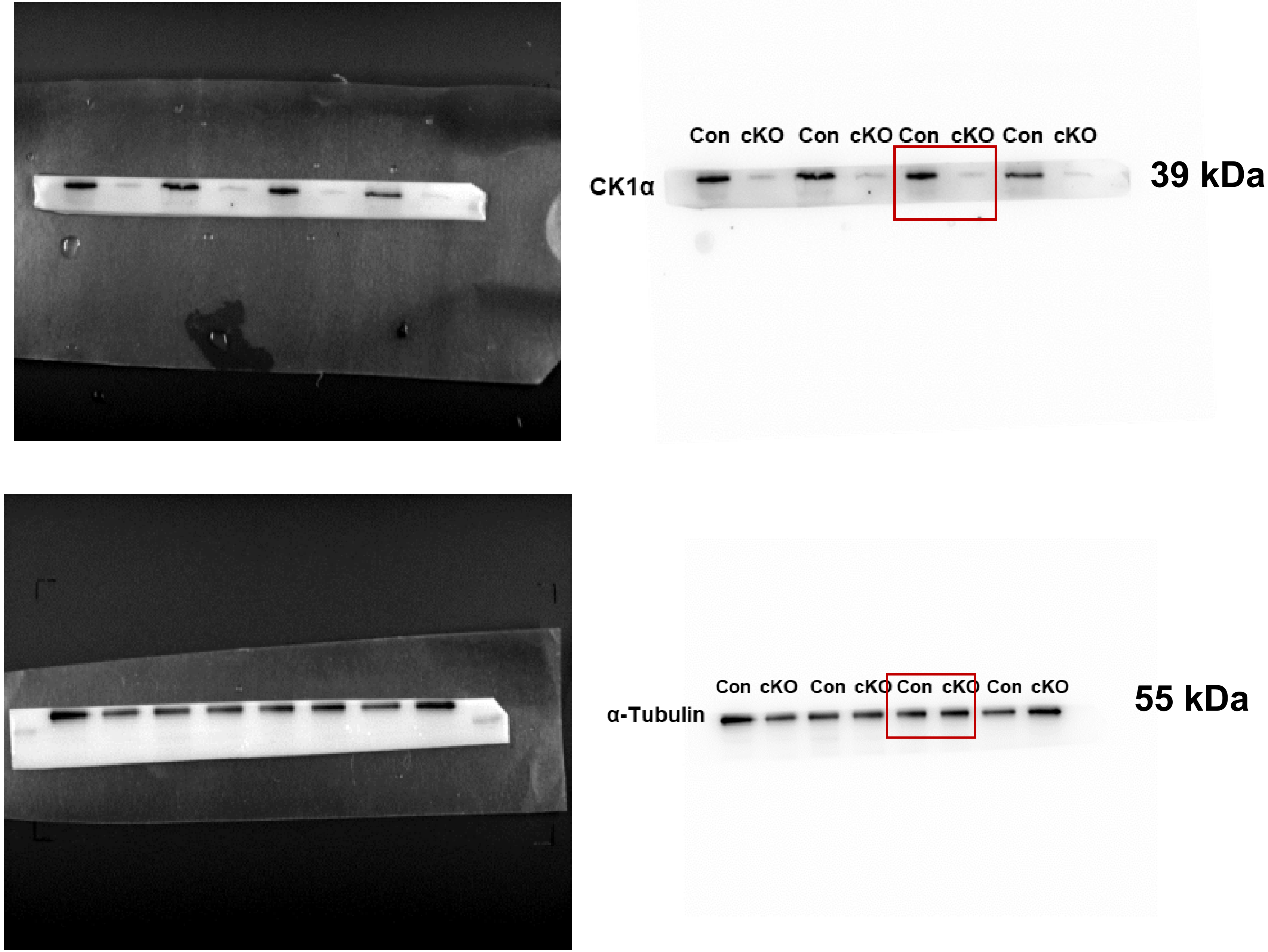

Con cKO Con cKO Con cKO Con cKO
CK1α
39 kDa
Con cKO Con cKO Con cKO Con cKO
α-Tubulin
55 kDa

Fig.5d

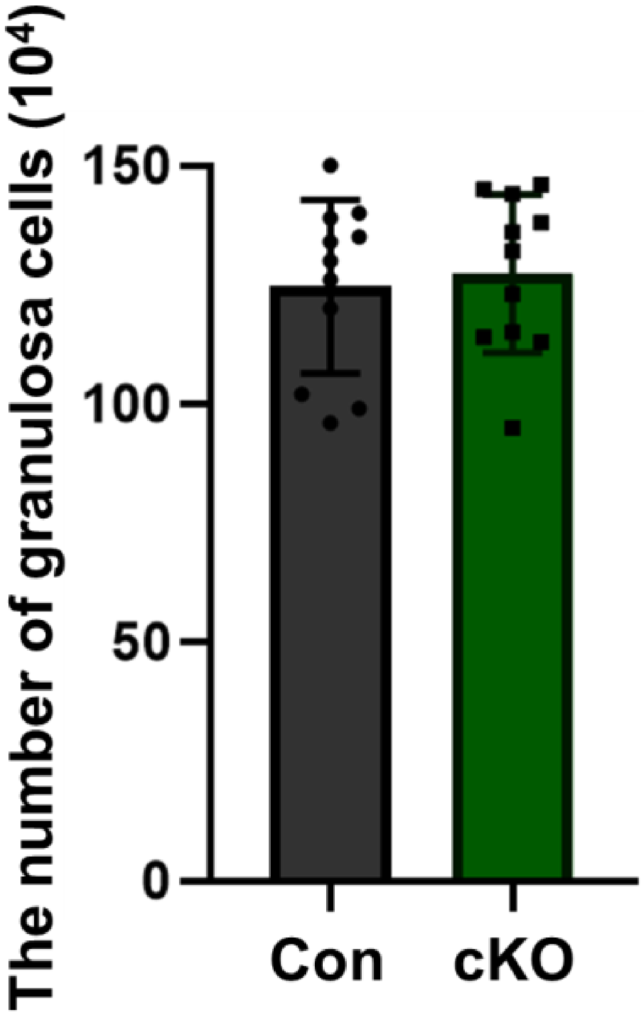

Fig.5e

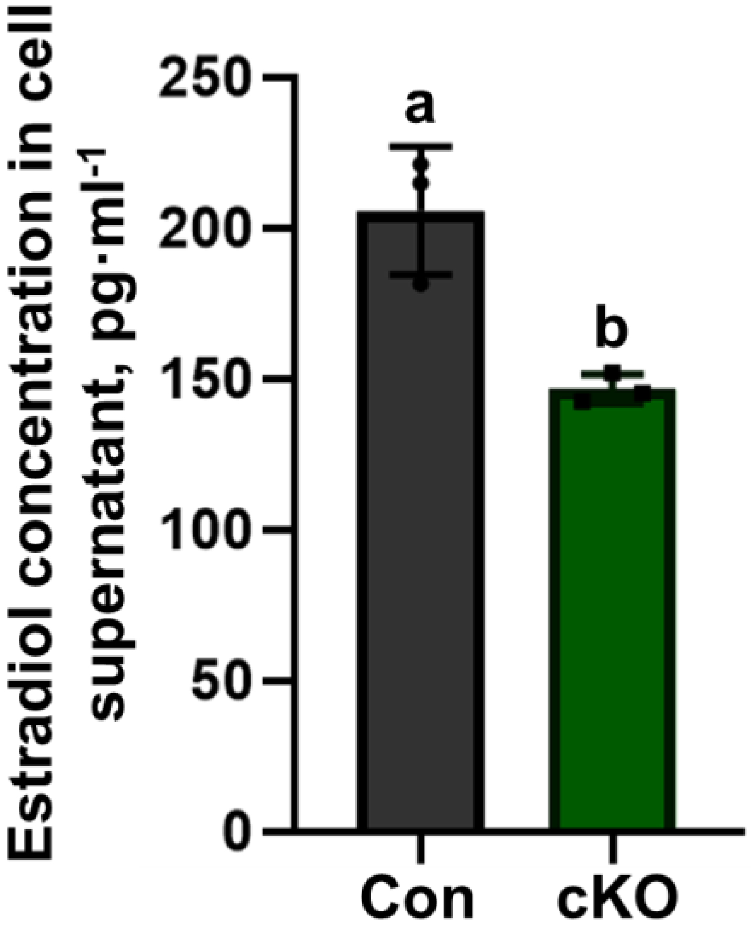
Estradiol concentration in cell supernatant, pg·ml-1
250
200
150
100
50
0
a
b
Con
cKO

Fig.5f

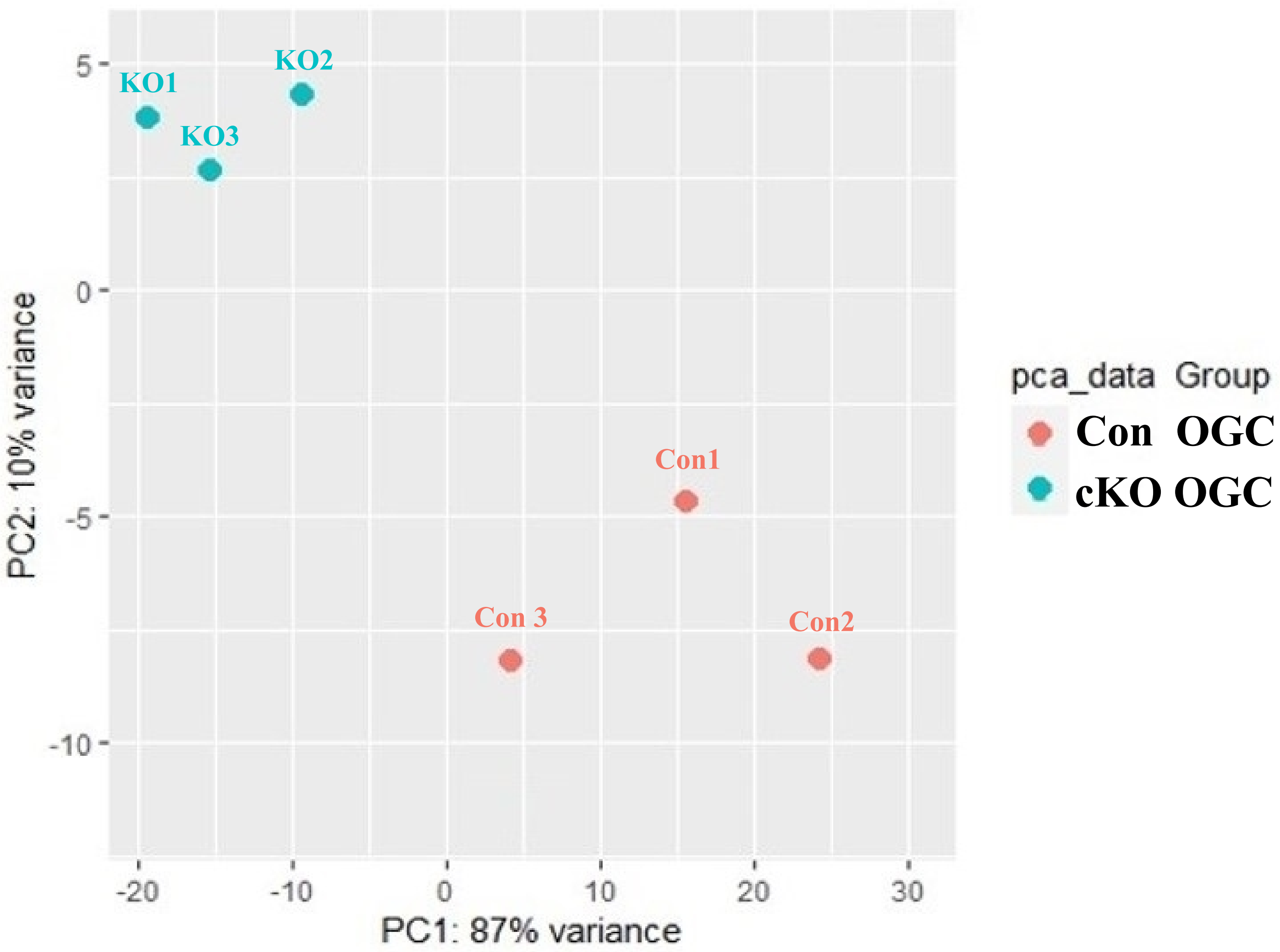

KO1
KO2
KO3
Con1
Con 3
Con2
5
0
-5
-10
-20
-10
0
10
20
30
PC1: 87% variance
PC2: 10% variance
pca_data Group
Con OGC
cKO OGC

Fig.5g

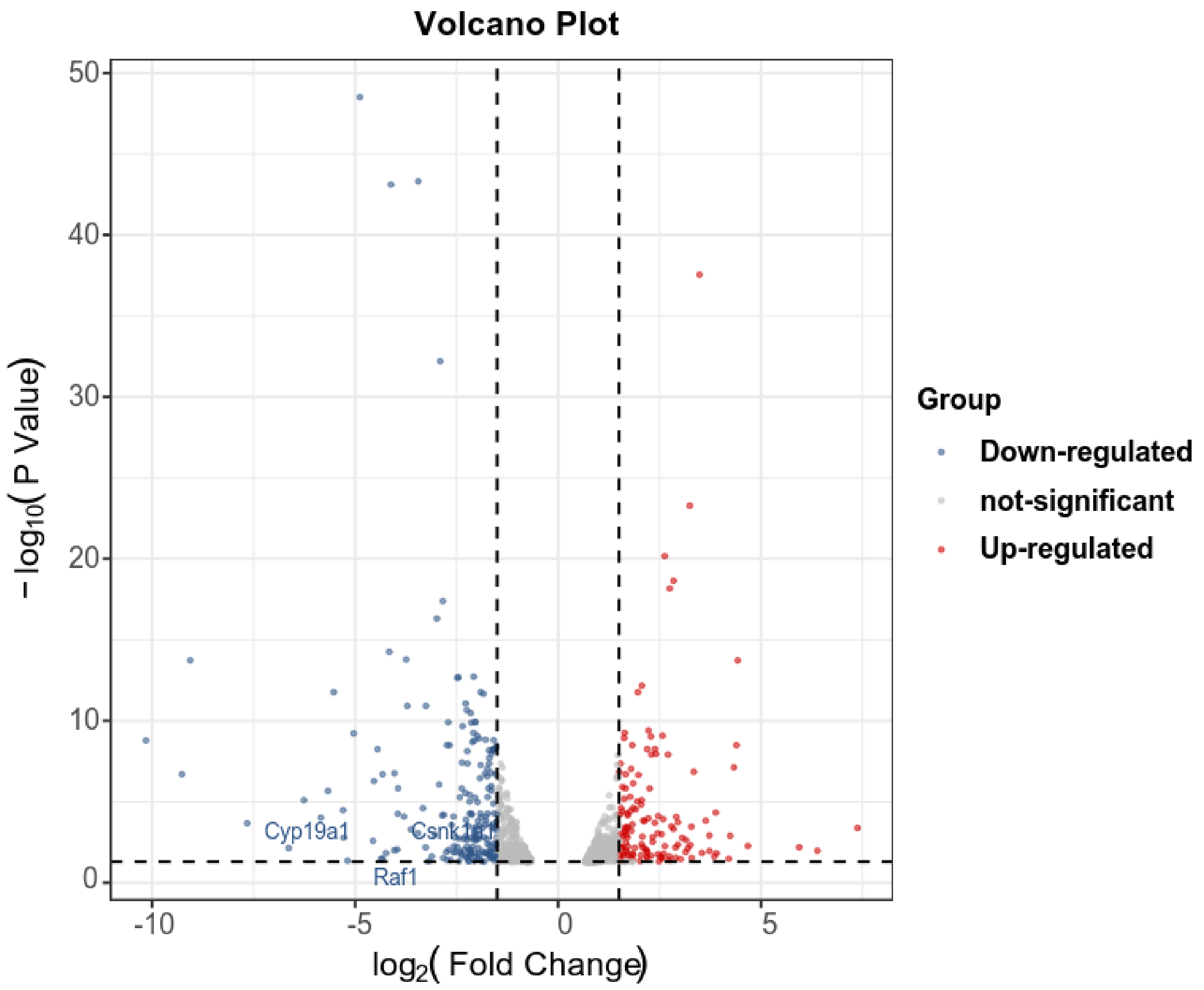
Volcano Plot
50
40
30
20
10
0
-10
-5
0
5
log2(Fold Change)
−log10(P Value)
Cyp19a1
Csnk1a1
Raf1
Group
Down-regulated
not-significant
Up-regulated

Fig.5h-5j

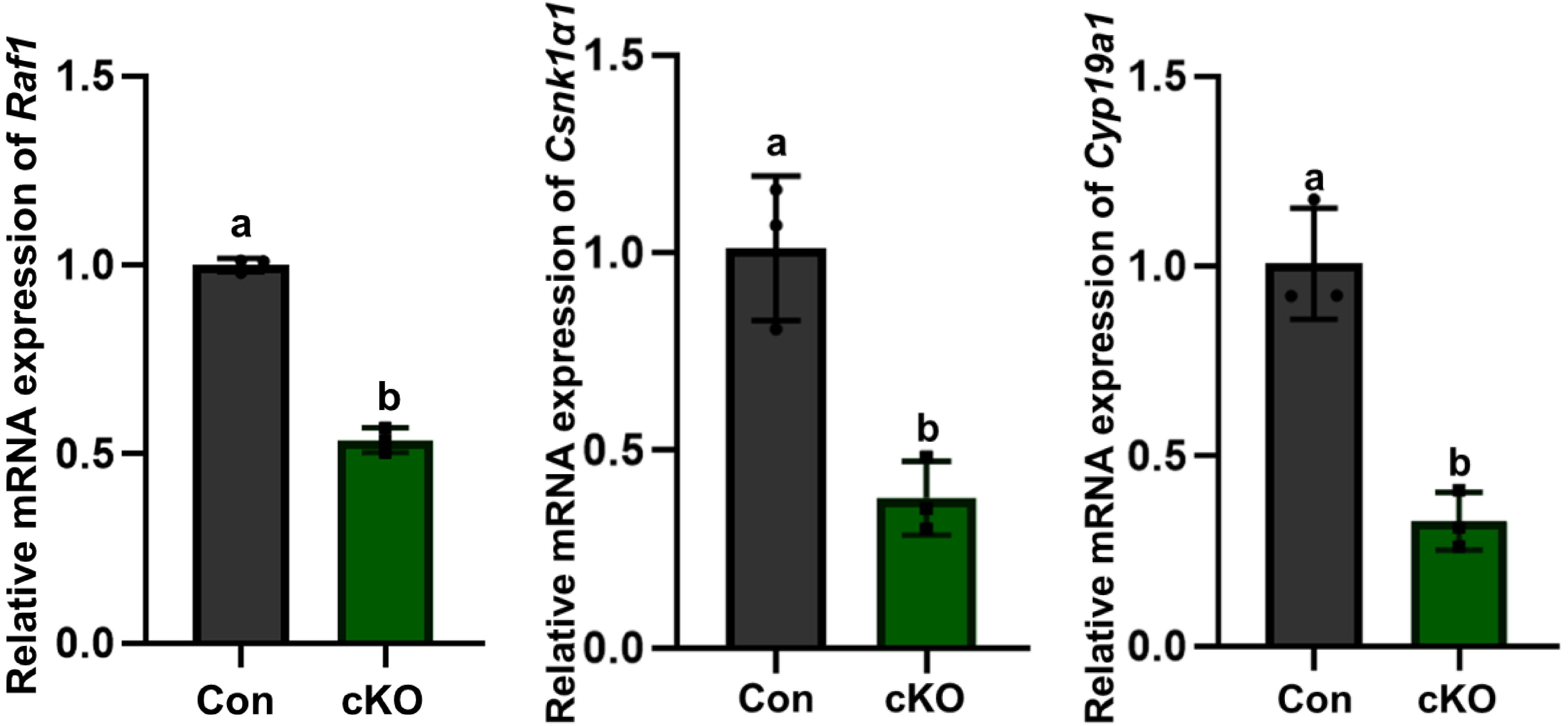
Relative mRNA expression of Raf1
1.5
1.0
0.5
0.0
a
b
Con
cKO
Relative mRNA expression of Csnk1α1
1.5
1.0
0.5
0.0
a
b
Con
cKO
Relative mRNA expression of Cyp19a1
1.5
1.0
0.5
0.0
a
b
Con
cKO

Fig.5k

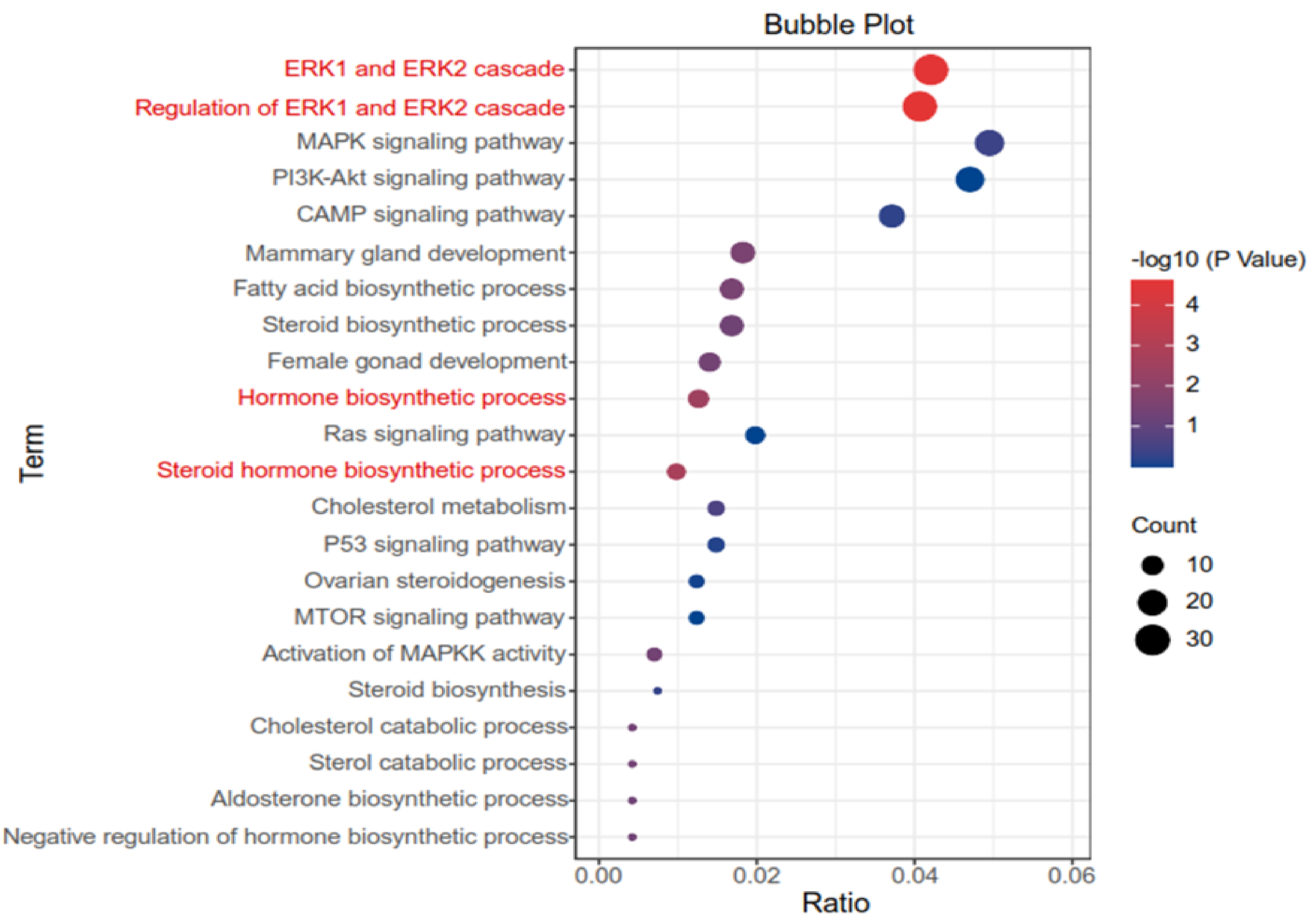
Bubble Plot
ERK1 and ERK2 cascade
Regulation of ERK1 and ERK2 cascade
MAPK signaling pathway
PI3K-Akt signaling pathway
CAMP signaling pathway
Mammary gland development
Fatty acid biosynthetic process
Steroid biosynthetic process
Female gonad development
Hormone biosynthetic process
Ras signaling pathway
Steroid hormone biosynthetic process
Cholesterol metabolism
P53 signaling pathway
Ovarian steroidogenesis
MTOR signaling pathway
Activation of MAPKK activity
Steroid biosynthesis
Cholesterol catabolic process
Sterol catabolic process
Aldosterone biosynthetic process
Negative regulation of hormone biosynthetic process
Term
0.00
0.02
0.04
0.06
Ratio
-log10 (P Value)
4
3
2
1
Count
10
20
30

Fig.5I

Cell samples

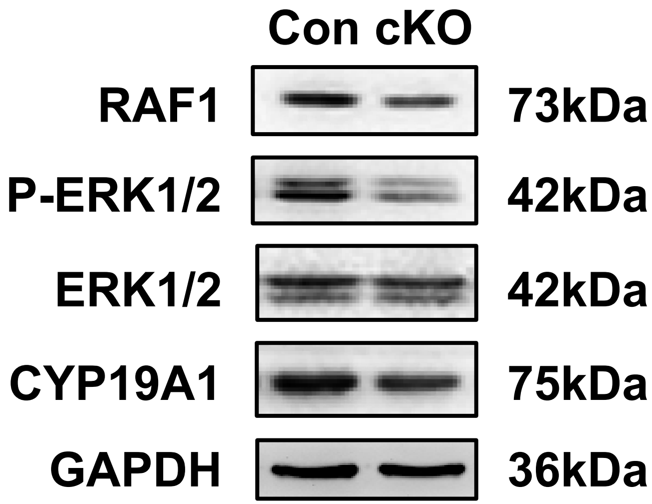

Fig.5l

Cell samples

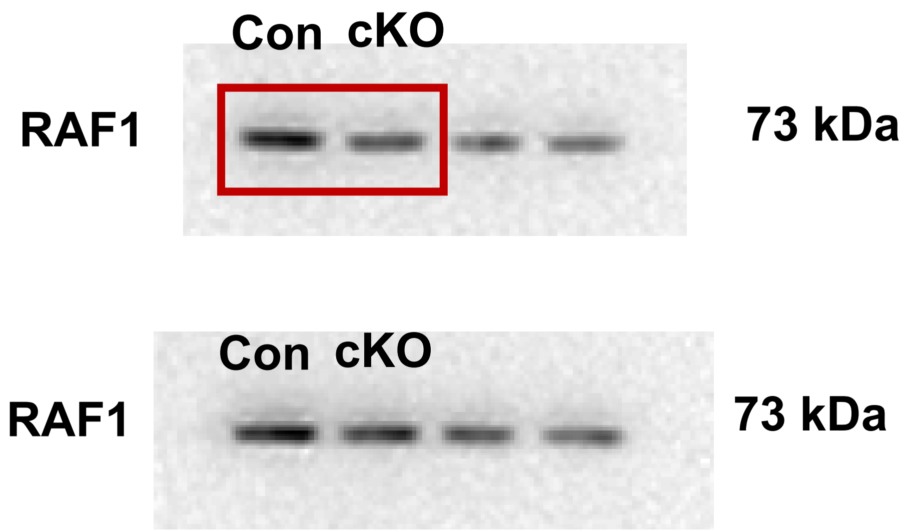

Fig.5I

Cell samples

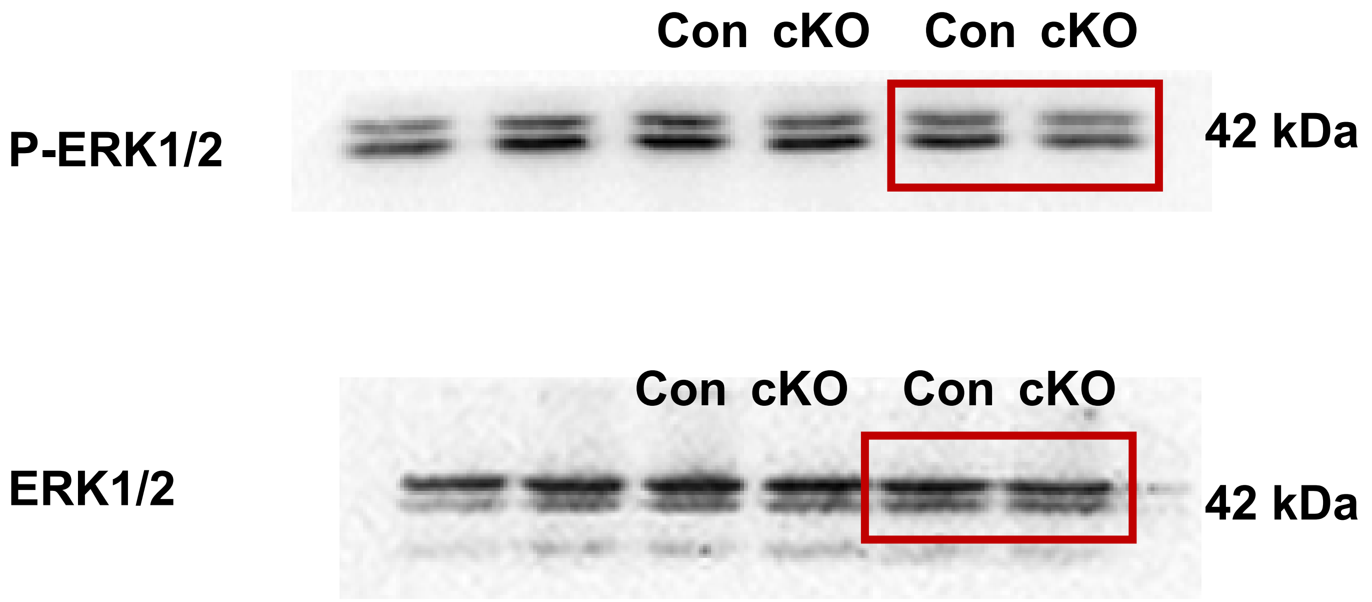

Fig.5I

Cell samples

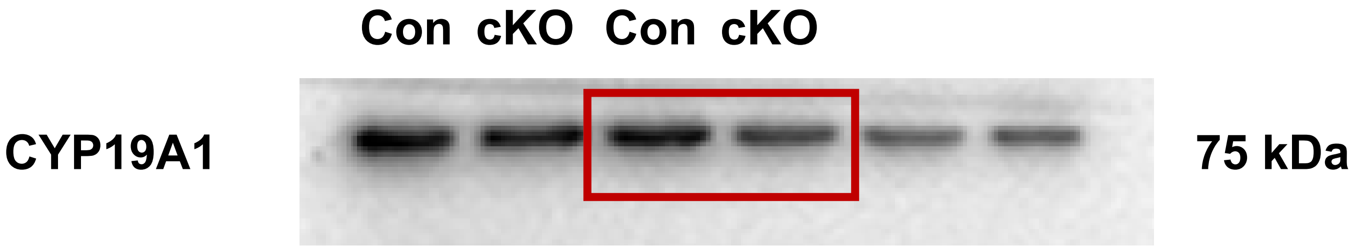

Fig.5I

Cell samples

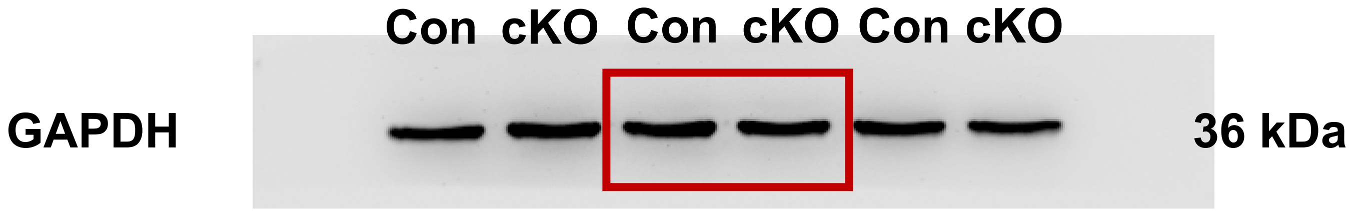

Fig.5m-5o

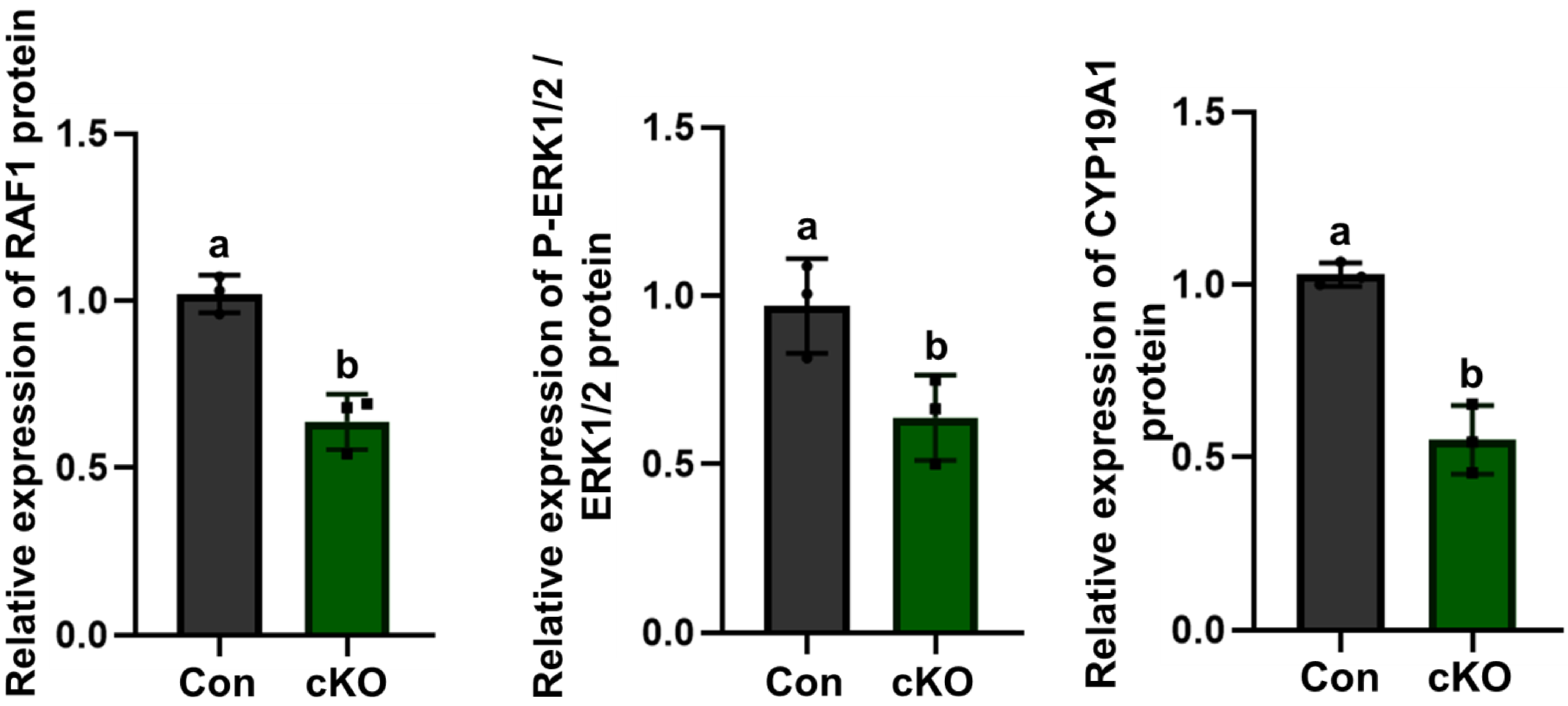

Relative expression of RAF1 protein
1.5
1.0
0.5
0.0
a
b
Con
cKO
Relative expression of P-ERK1/2 / ERK1/2 protein
1.5
1.0
0.5
0.0
a
b
Con
cKO
Relative expression of CYP19A1 protein
1.5
1.0
0.5
0.0
a
b
Con
cKO

Fig.6

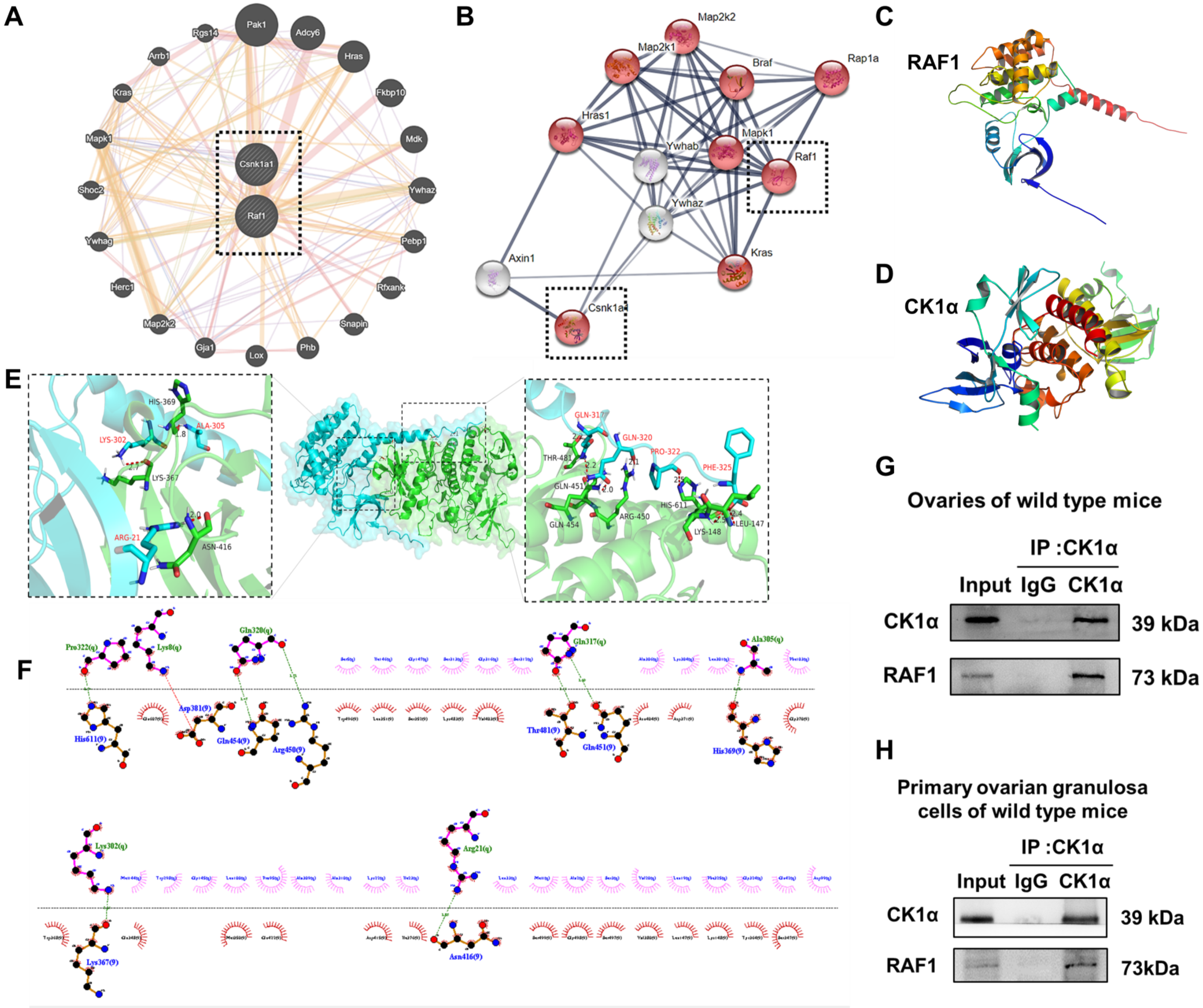
A
Pak1
Adcy6
Rgs14
Arrb1
Hras
Kras
Fkbp10
Mapk1
Mdk
Csnk1a1
Shoc2
Ywhaz
Raf1
Ywhag
Pebp1
Herc1
Rfxank
Map2k2
Snapin
Gja1
Lox
Phb
B
Map2k2
Map2k1
Braf
Rap1a
Hras1
Mapk1
Ywhab
Raf1
Ywhaz
Axin1
Kras
Csnk1a
C
RAF1
D
CK1α
E
HIS-369
ALA-305
LYS-302
LYS-367
ARG-21
ASN-416
GLN-317
GLN-320
PRO-322
PHE-325
THR-481
GLN-451
ARG-450
HIS-611
GLN 454
LYS-148
LEU-147
F
G
Ovaries of wild type mice
IP :CK1α
Input IgG CK1α
CK1α
39 kDa
RAF1
73 kDa
H
Primary ovarian granulosa cells of wild type mice
IP :CK1α
Input IgG CK1α
CK1α
39 kDa
RAF1
73kDa

Fig.6a

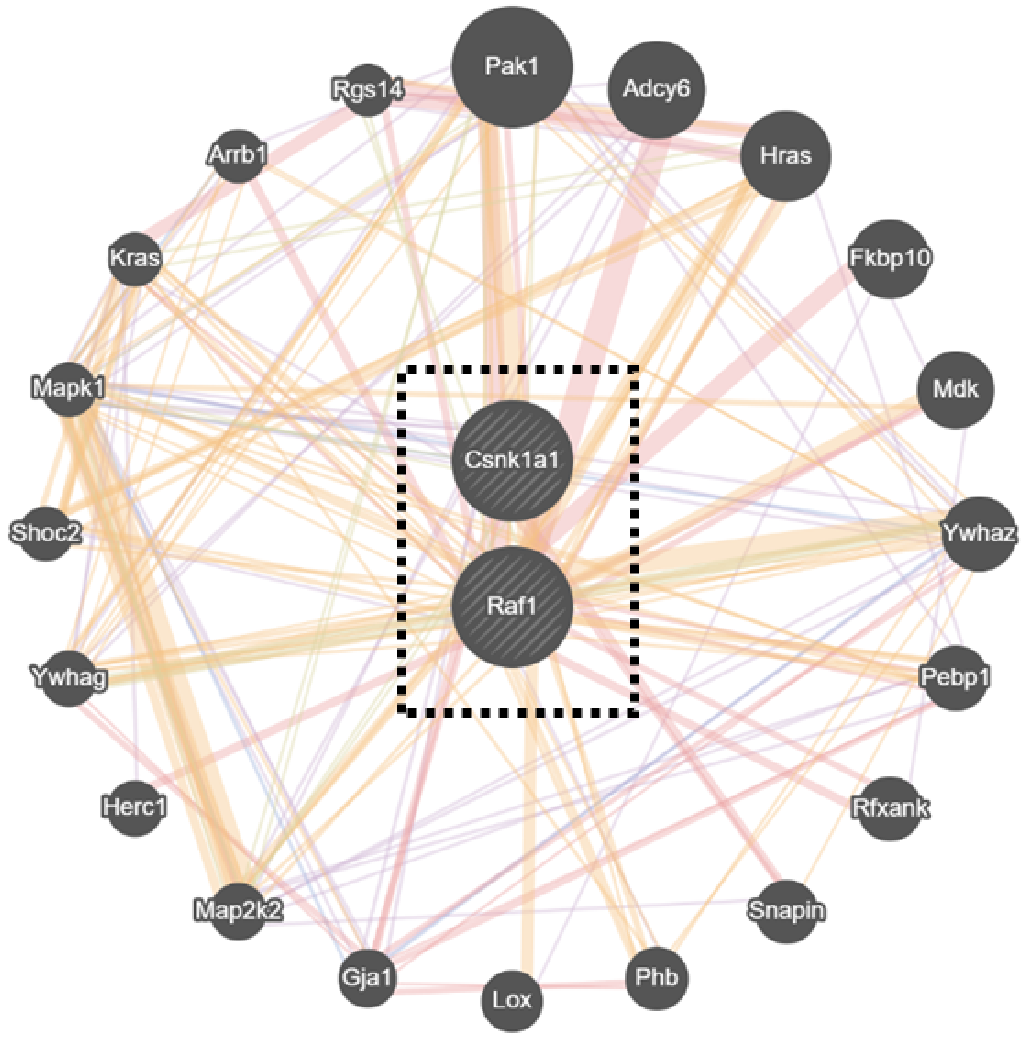

https://apps.pathwaycommons.org/interactions?source=CSNK1A1

Fig.6b

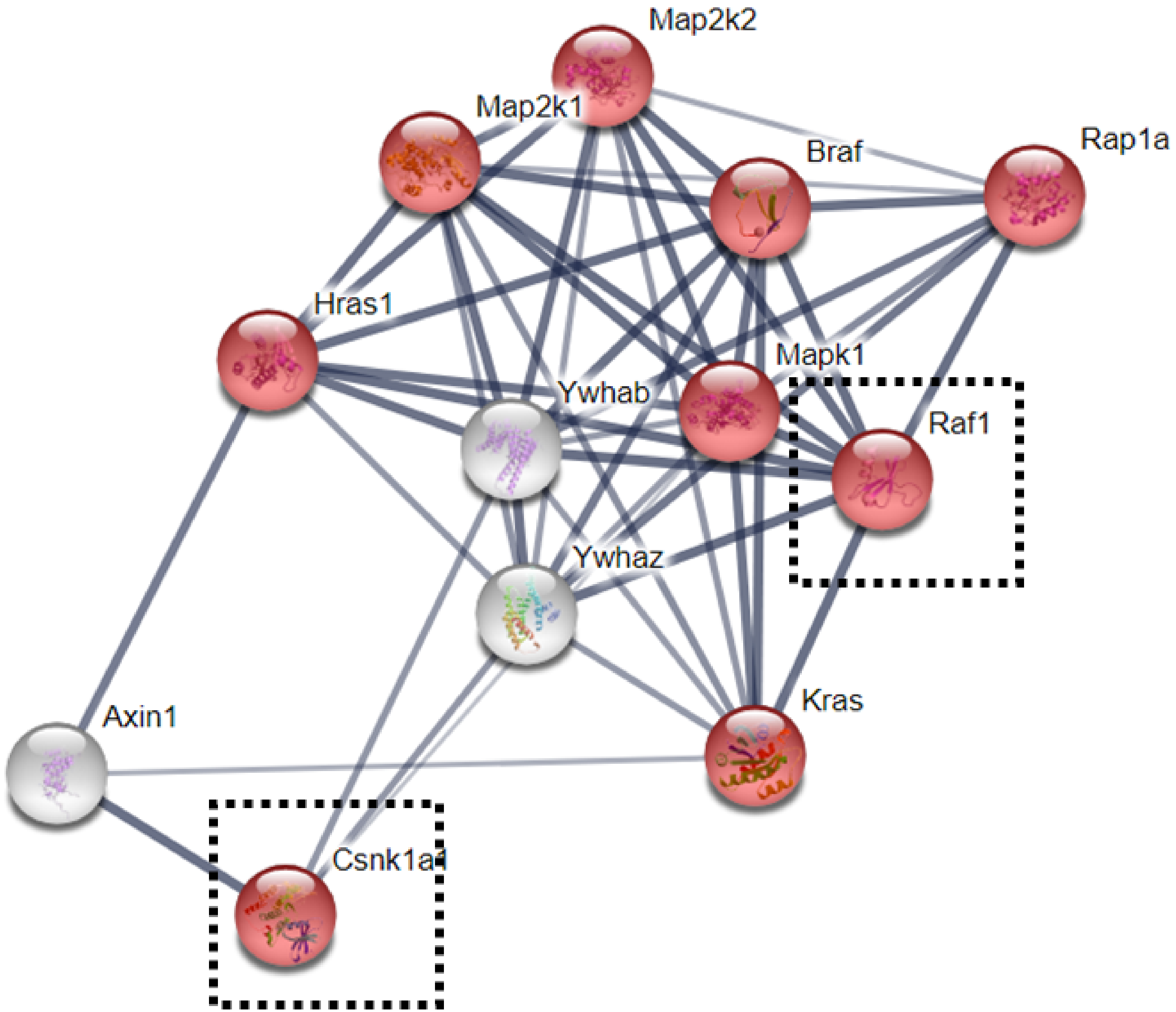

http://stitch.embl.de/cgi/network.pl?taskId=wJA6cX8ZUKx3

Fig.6c,d

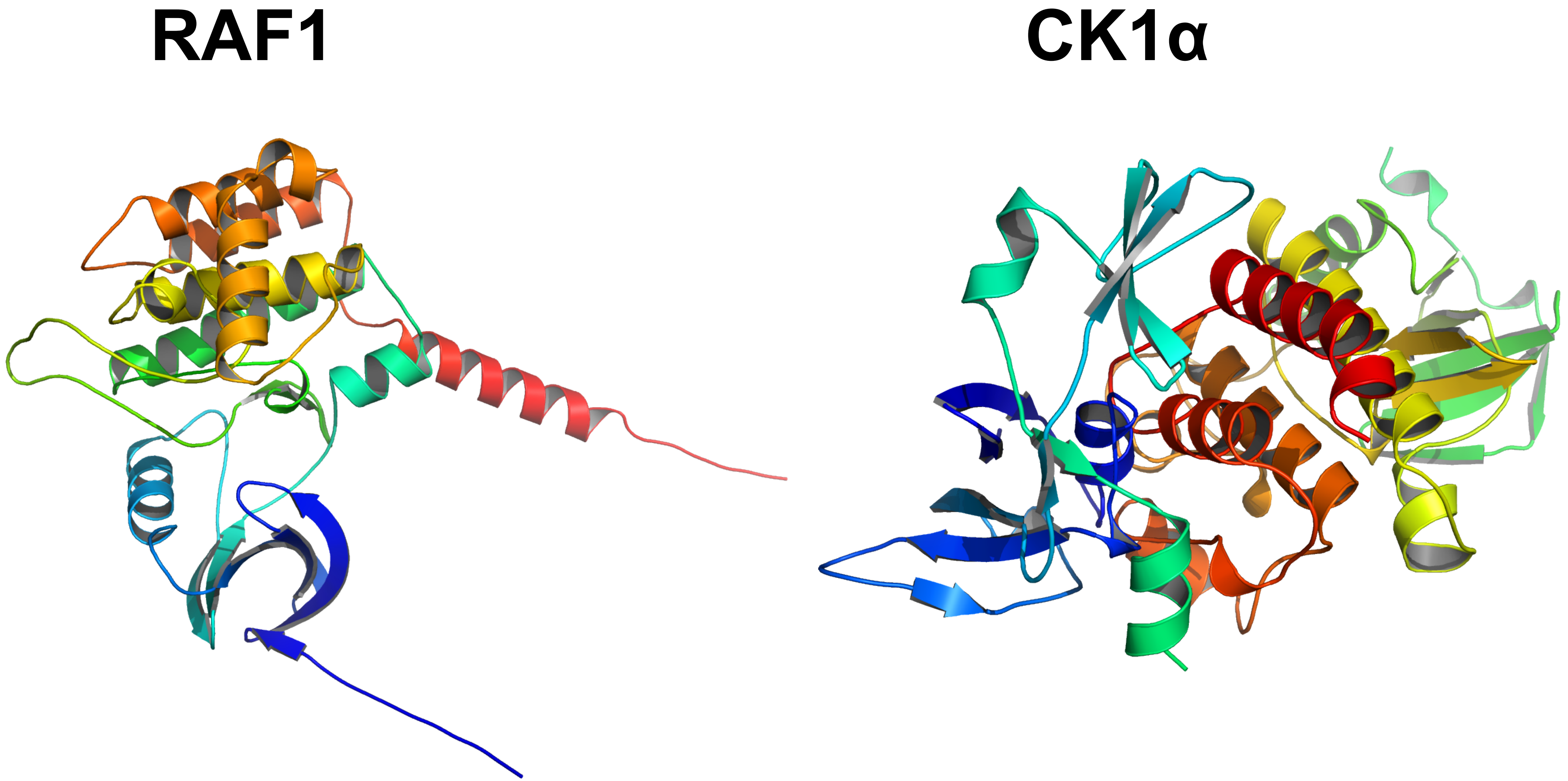

Fig.6e

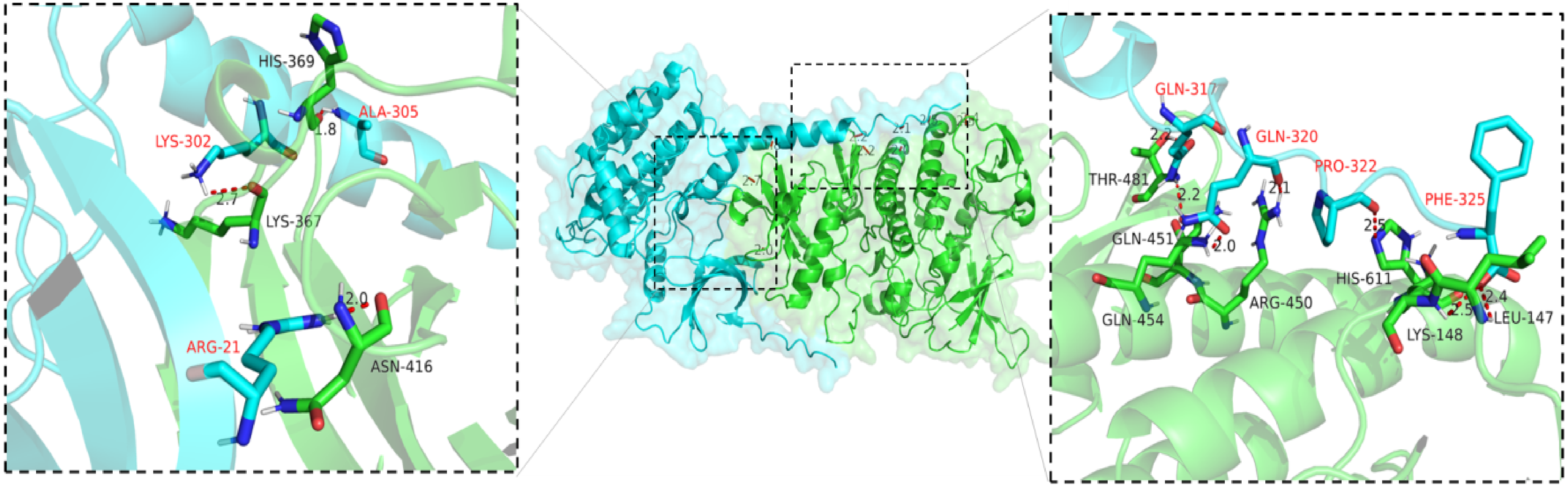
HIS-369
ALA-305
LYS-302
1.8
2.7
LYS-367
2.0
ARG-21
ASN-416
GLN-317
GLN-320
PRO-322
PHE-325
THR-481
2.2
2.2
2.1
GLN-451
2.0
2.5
HIS-611
ARG-450
2.4
2.5
LEU-147
GLN-454
LYS-148

Fig.6f

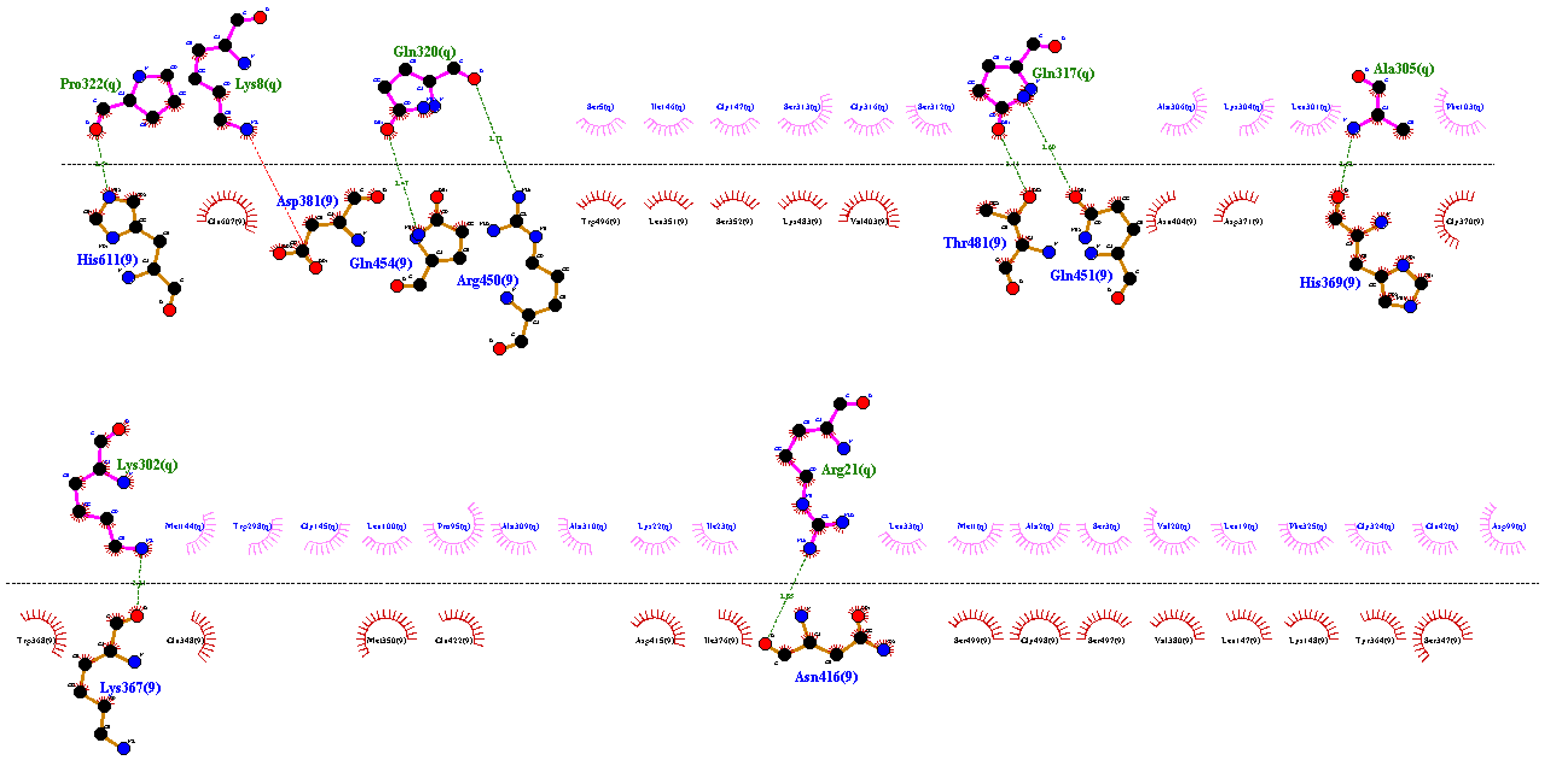
Pro322(q)
Lys8(q)
Gln320(q)
Gln317(q)
Ala305(q)
His611(9)
Asp381(9)
Gln454(9)
Arg450(9)
Thr481(9)
Gln451(9)
His369(9)
Lys302(q)
Arg21(q)
Lys367(9)
Asn416(9)

Fig.6g,h

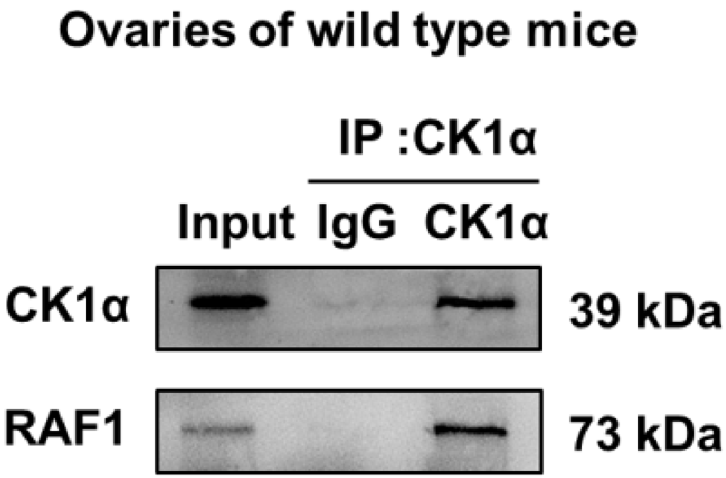
Ovaries of wild type mice
IP :CK1α
Input
IgG
CK1α
CK1α
39 kDa
RAF1
73 kDa

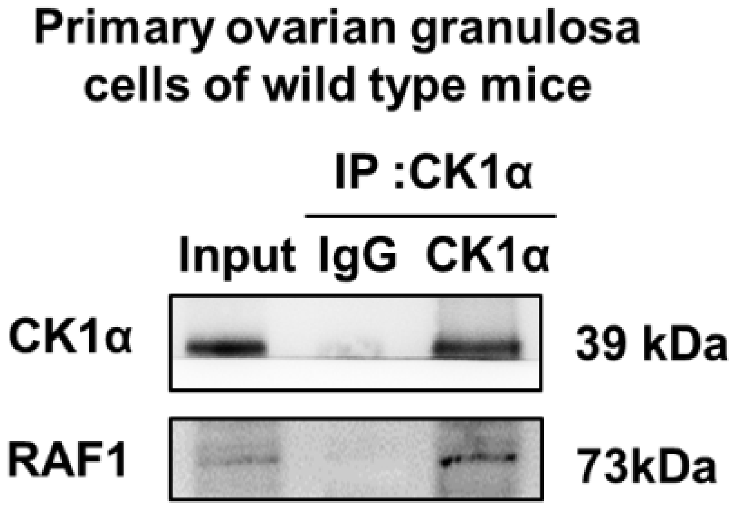
Primary ovarian granulosa cells of wild type mice
IP :CK1α
Input
IgG
CK1α
CK1α
39 kDa
RAF1
73kDa

Ovaries of wild type mice

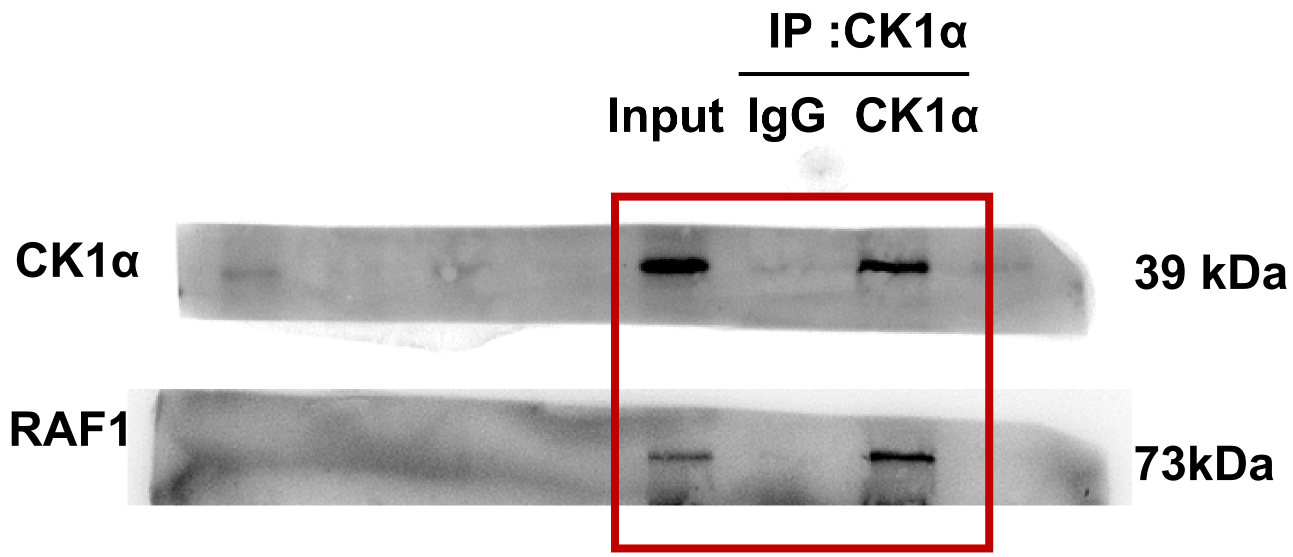

Primary ovarian granulosa cells of wild type mice

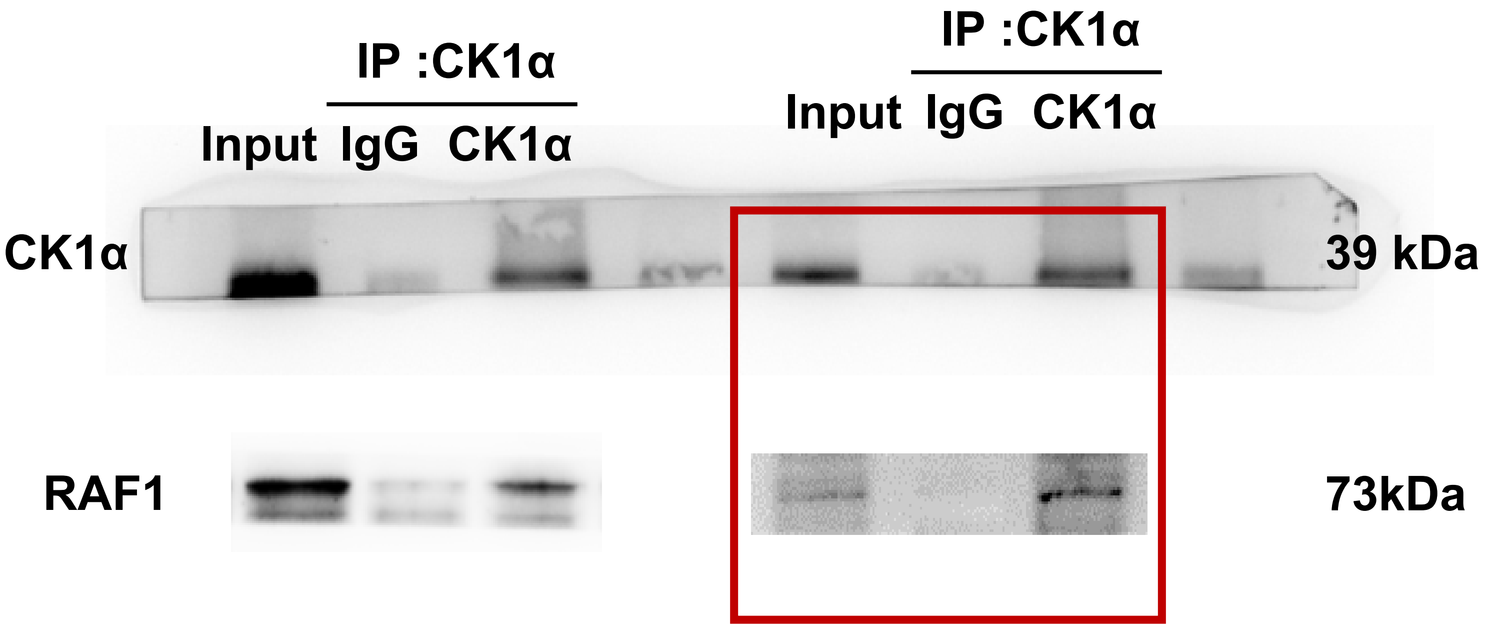

Supplement: Supplementary file 14 — Additional file 14. The raw data [file 12915_2024_1957_MOESM14_ESM.pdf]
